# Supplementary material for: Underlying Mechanisms of Reductive Amination on Pd-Catalysts: The Unique Role of Hydroxyl Group in Generating Sterically Hindered Amine
Source: Int J Mol Sci. 2022 Jul 10;23(14):7621. doi: 10.3390/ijms23147621 (PMC9320161; doi:10.3390/ijms23147621)
Supplement: Supplementary file 1 [file ijms-23-07621-s001.zip › ijms-1790296-supplementary.pdf]

Supporting Information

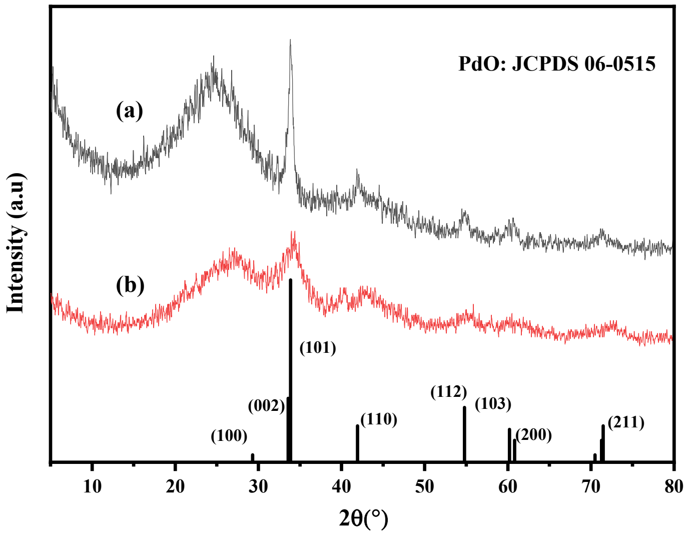

Figure S1. XRD patterns of the selected catalysts. (**a**) PdO/ACs treated by HNO_3, (**b**) commercially 10 wt% $Pd(OH)_2$/ACs.

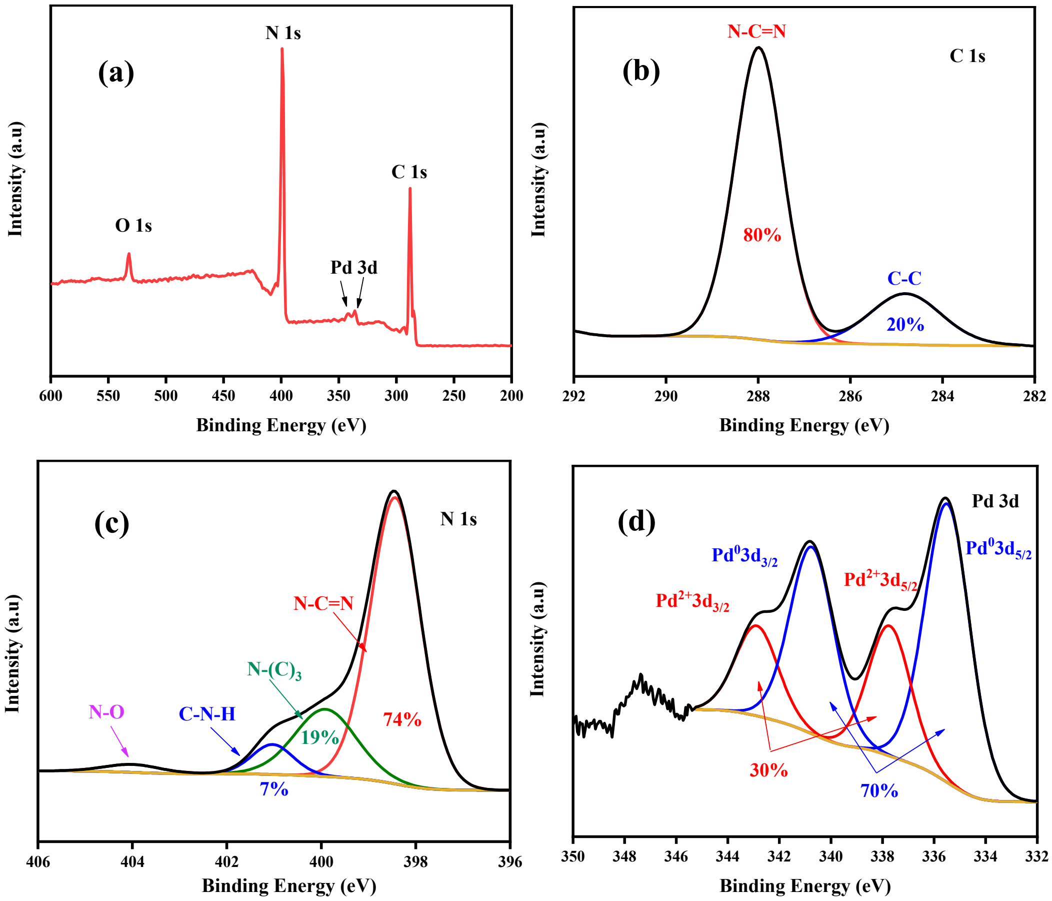

Figure S2. (**a**) (**b**) XPS survey spectrum and core level spectra of C1s, (**c**) N 1s, (**d**) Pd $3d_{5/2}$ and $3d_{3/2}$ doublet region of 1.23 wt% Pd/g-C_3N_4.

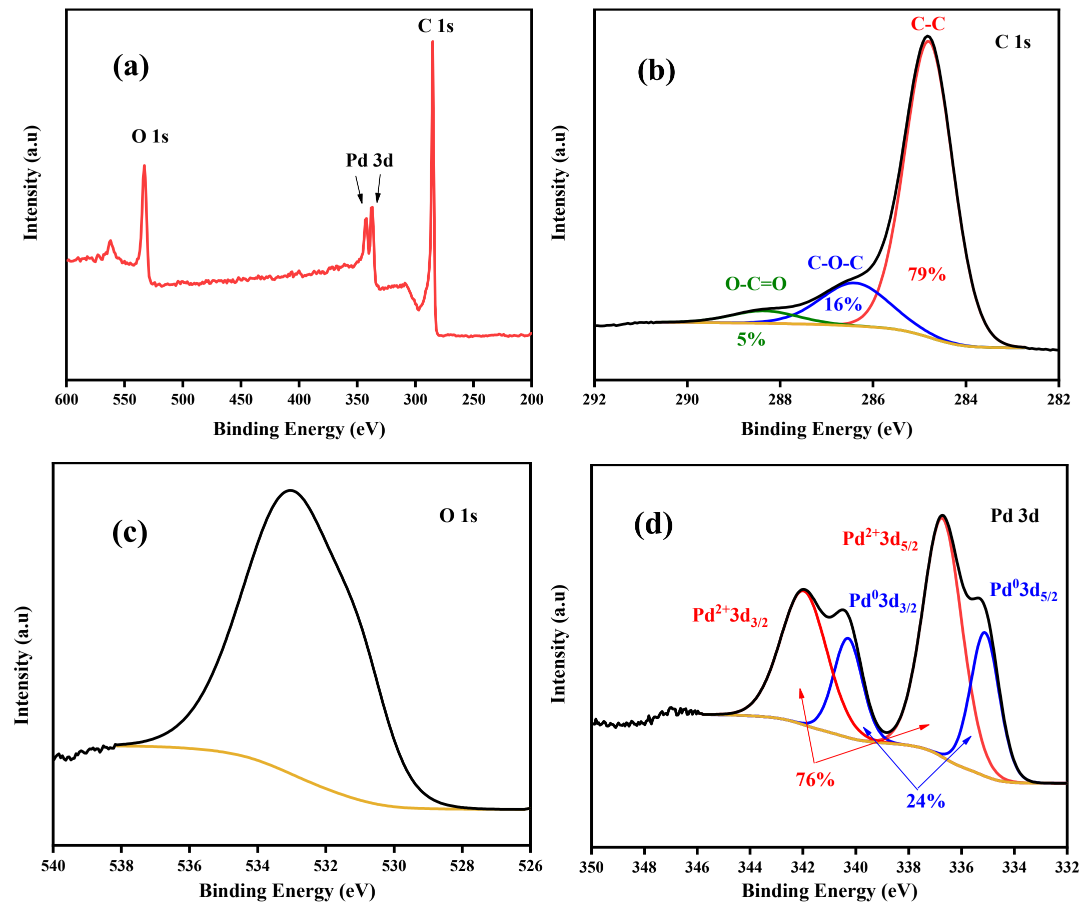

Figure S3. (**a**) (**b**) XPS survey spectrum and core level spectra of C1s, (**c**) O 1s, (**d**) Pd $3d_{5/2}$ and $3d_{3/2}$ doublet region of 1.35 wt% $Pd(OH)_2$/ACs that support treated by nitric acid.

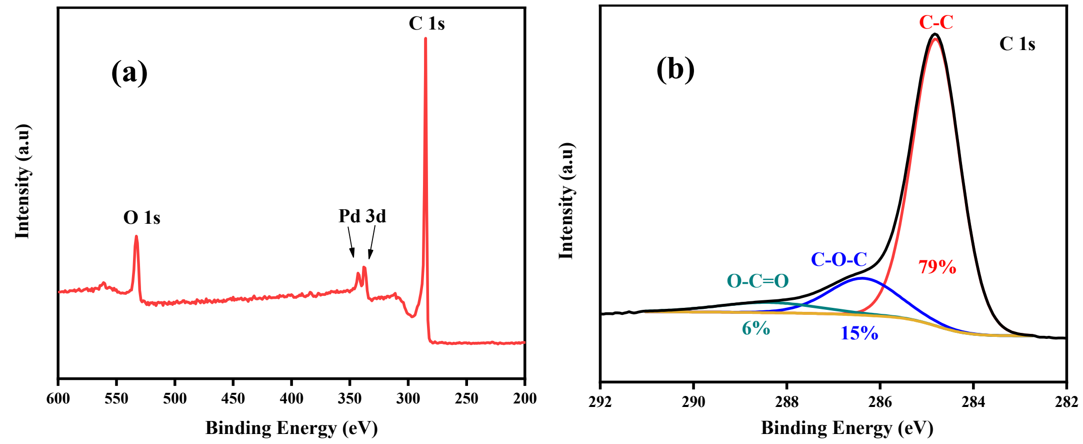

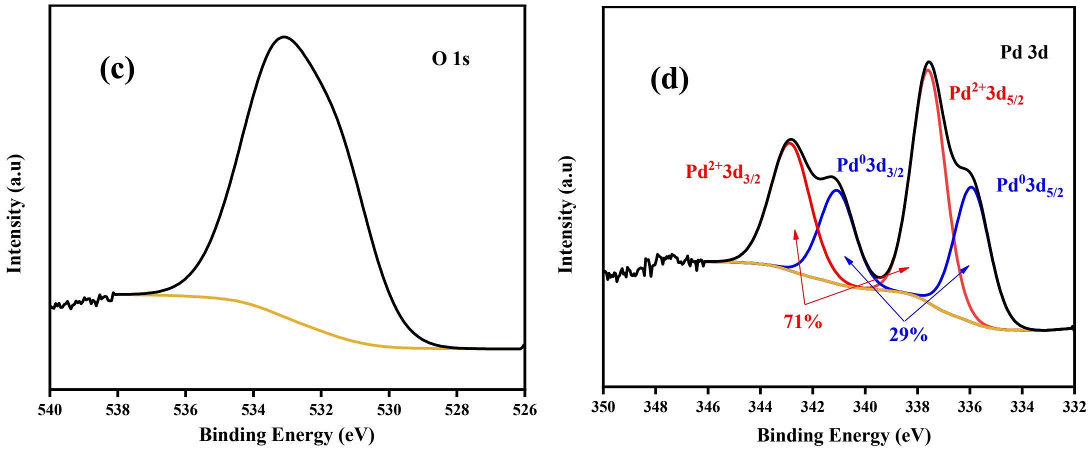

Figure S4. (**a**) (**b**) XPS survey spectrum and core level spectra of C1s, (**c**) O 1s, (**d**) Pd $3d_{5/2}$ and $3d_{3/2}$ doublet region of 1.19 wt% $Pd(OH)_2$/ACs that support by water.

Noticed that the broad O1s region can be overlapped by Pd 3p3 peaks (see the "Handbook of The Elements and Native Oxides, BE Lookup Table for Signals from Elements and Common Chemical Species"), so O1s spectrums (Figure S3c and S4c) are presented without peak splitting.

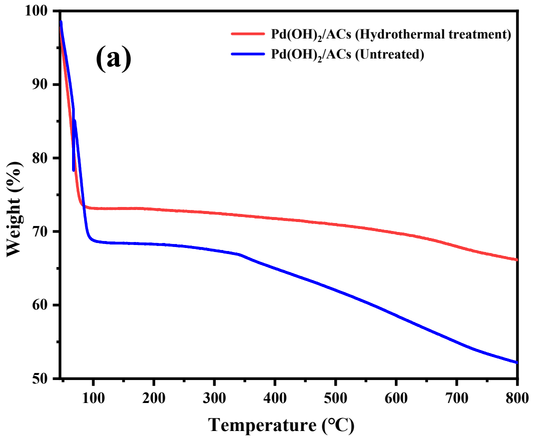

Figure S5. Thermogravimetric plots for $Pd(OH)_2$/ACs

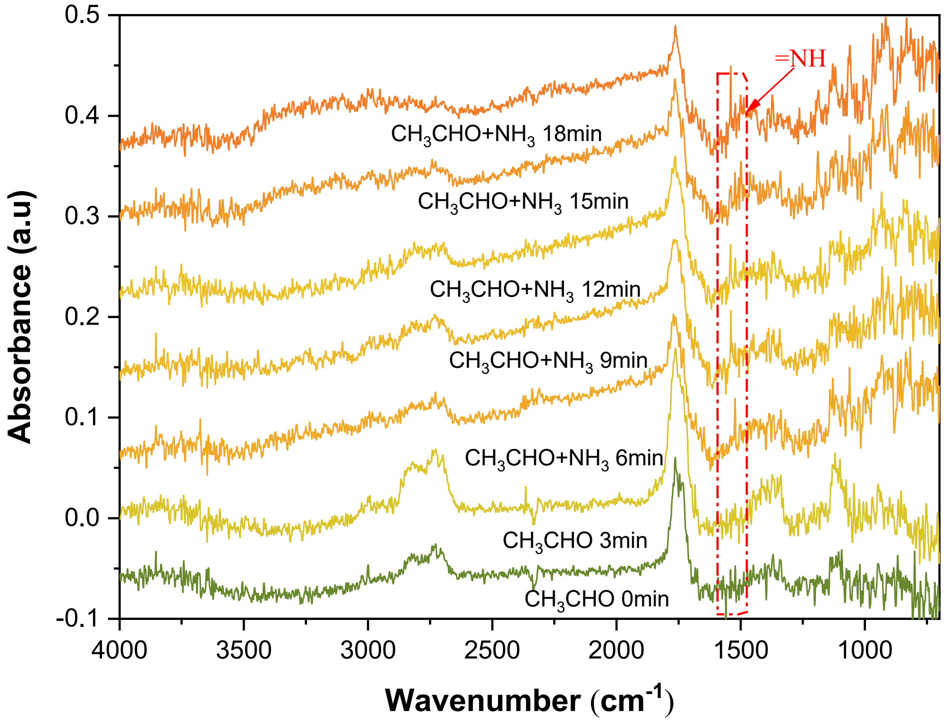

Figure S6. ATR-FTIR spectra recorded during generation of imine on activated carbon.

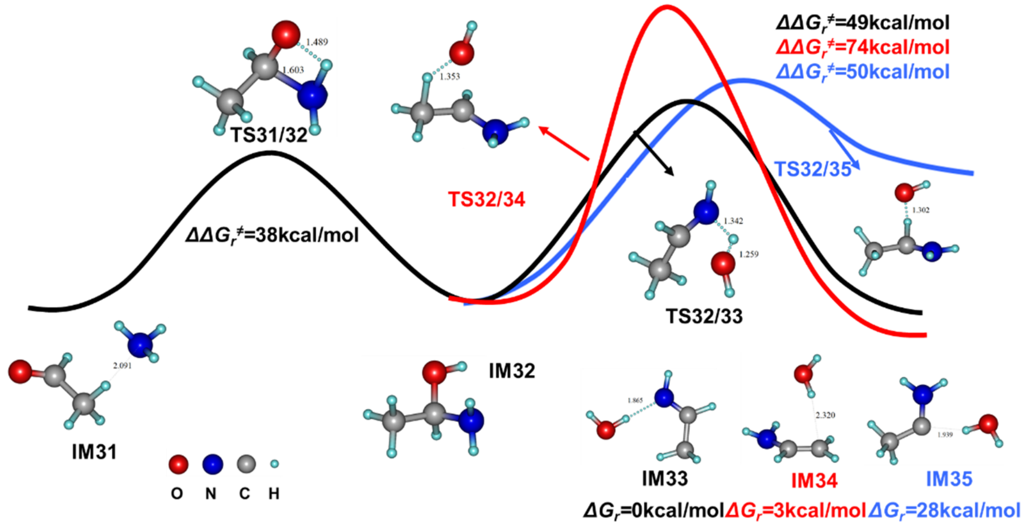

Figure S7. Simplified PES and selected structural information of alternative pathways for the generation of ethylamine.

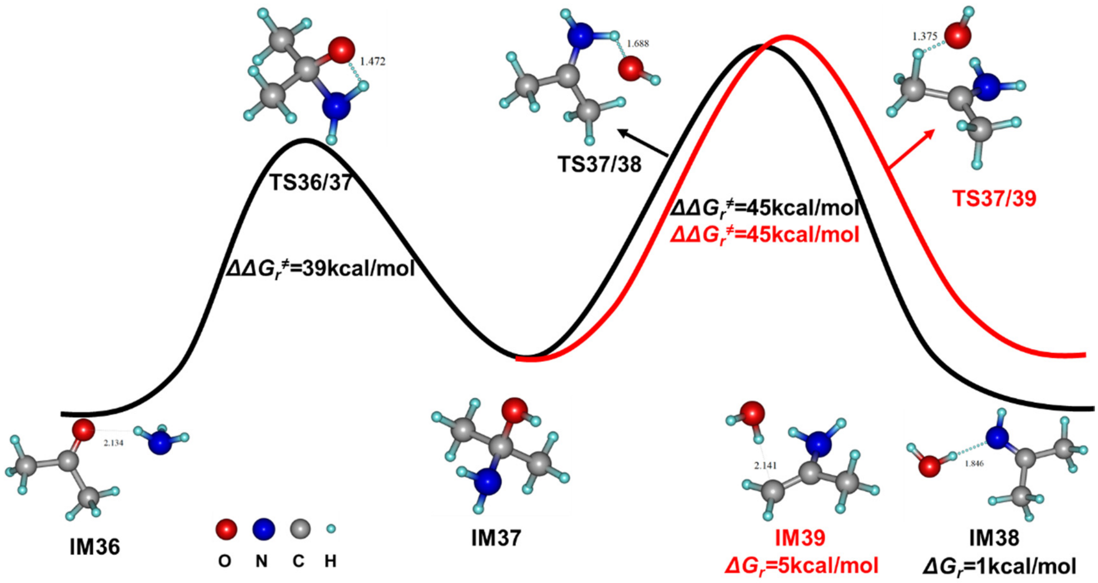

Figure S8. Simplified PES and selected structural information of alternative pathways for the generation of isopropylimine.

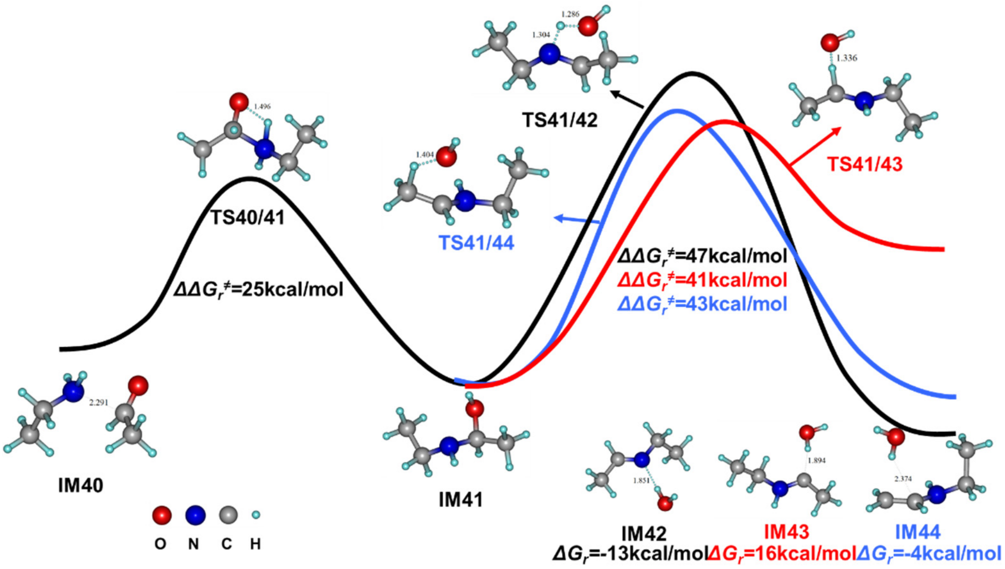

Figure S9. Simplified PES and selected structural information of alternative pathways for the generation of ethanamine.

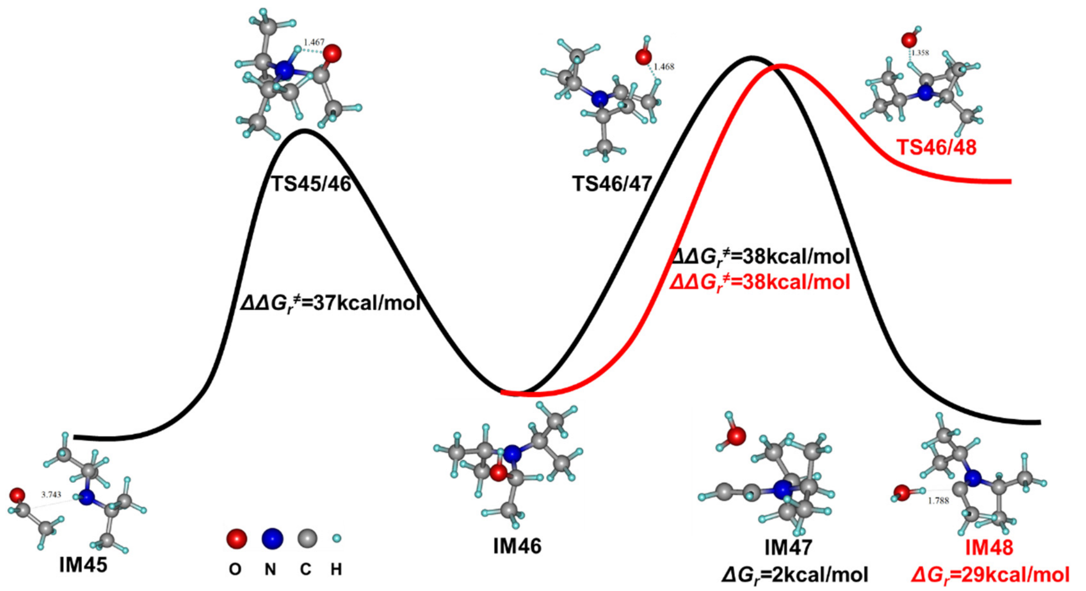

Figure S10. Simplified PES and selected structural information of alternative pathways for the generation of diisopropylethylimine.

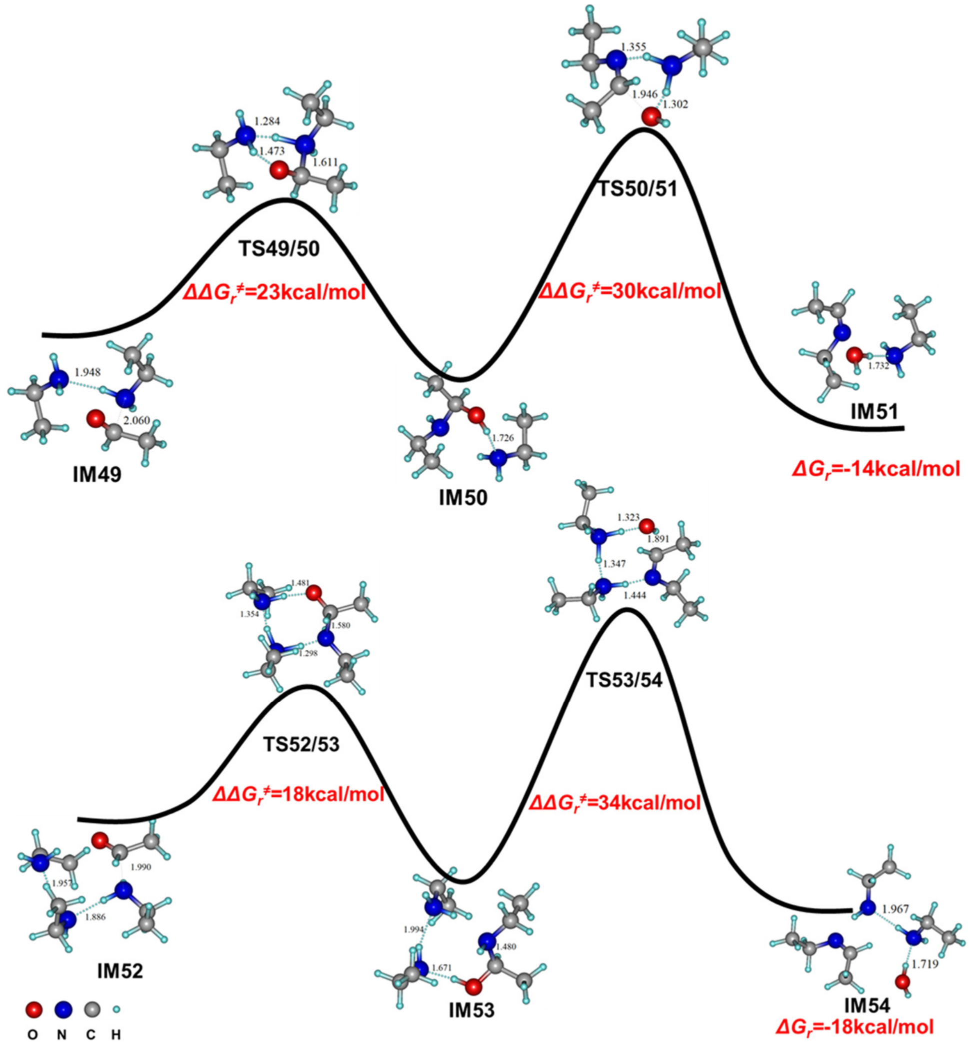

Figure S11. Simplified PES and selected structural information of alternative pathways for the generation of ethanamine catalyzed by amine itself.

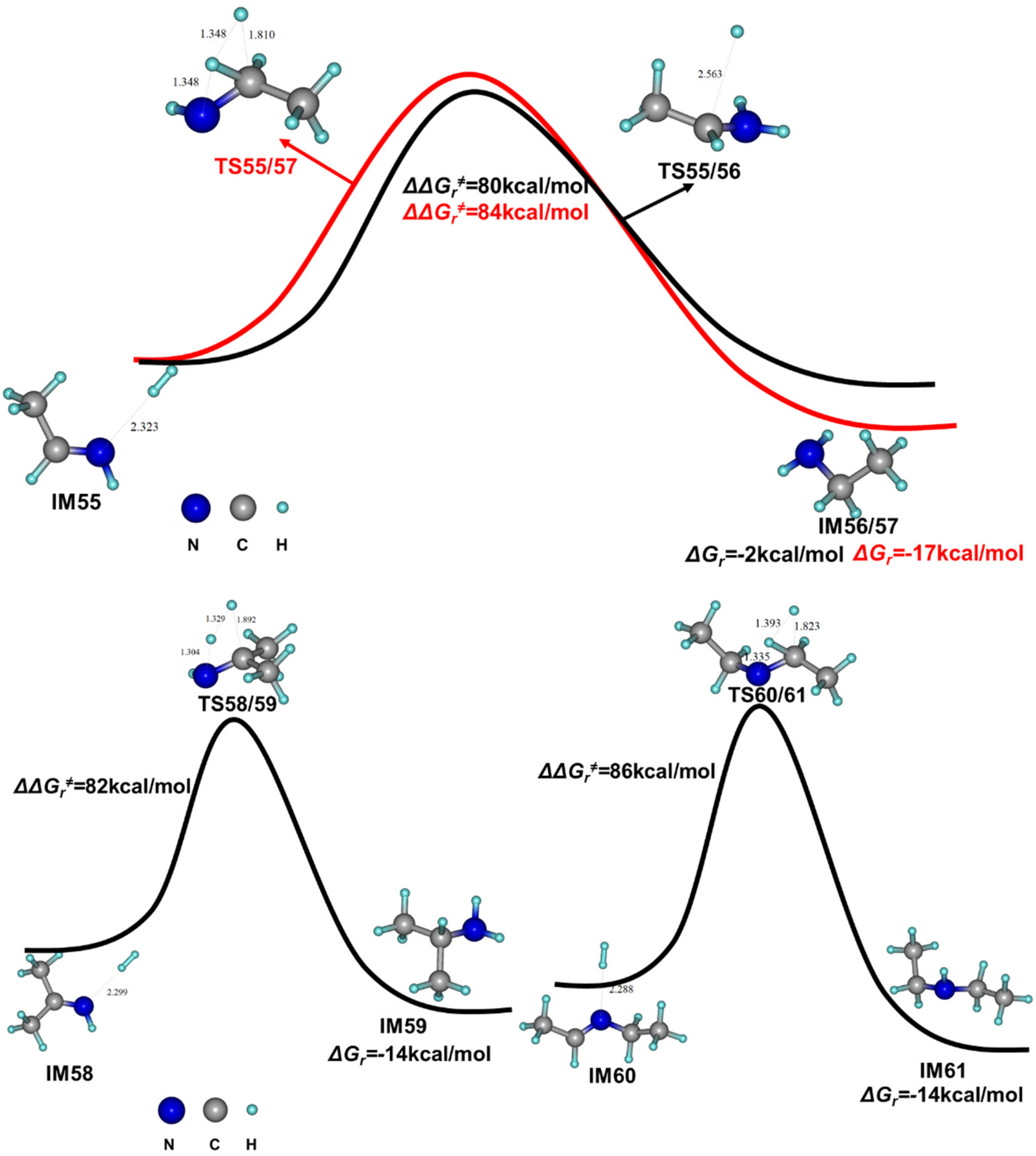

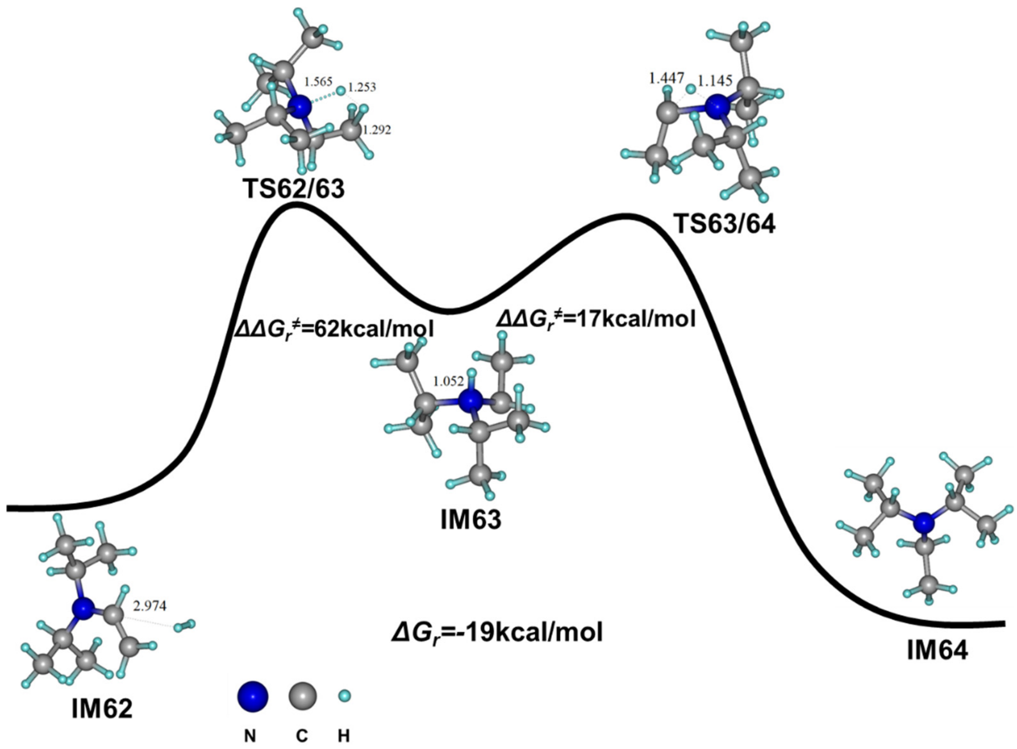

Figure S12. Simplified PES and selected structural information of alternative pathways for the hydrogenation of imine.

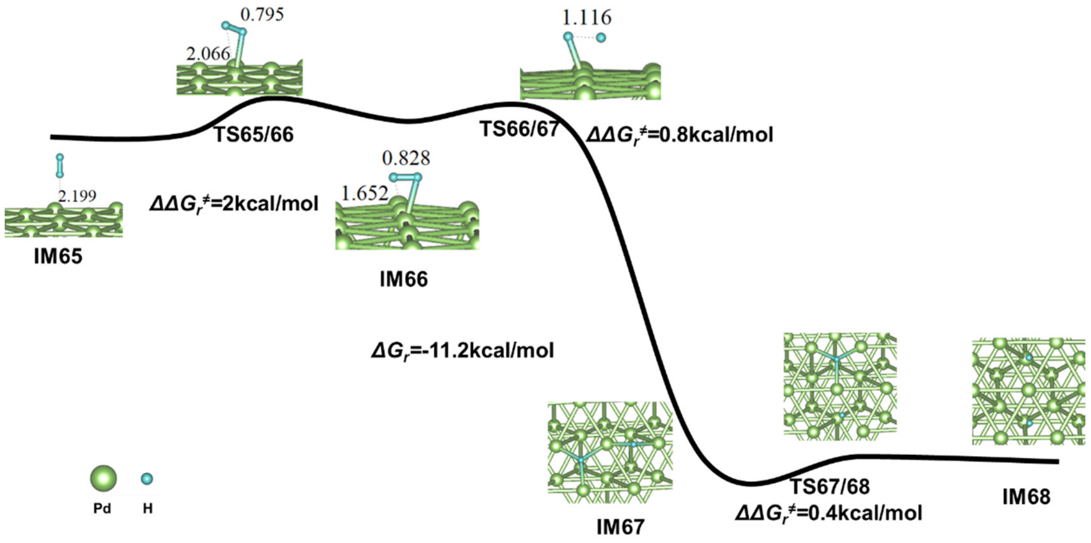

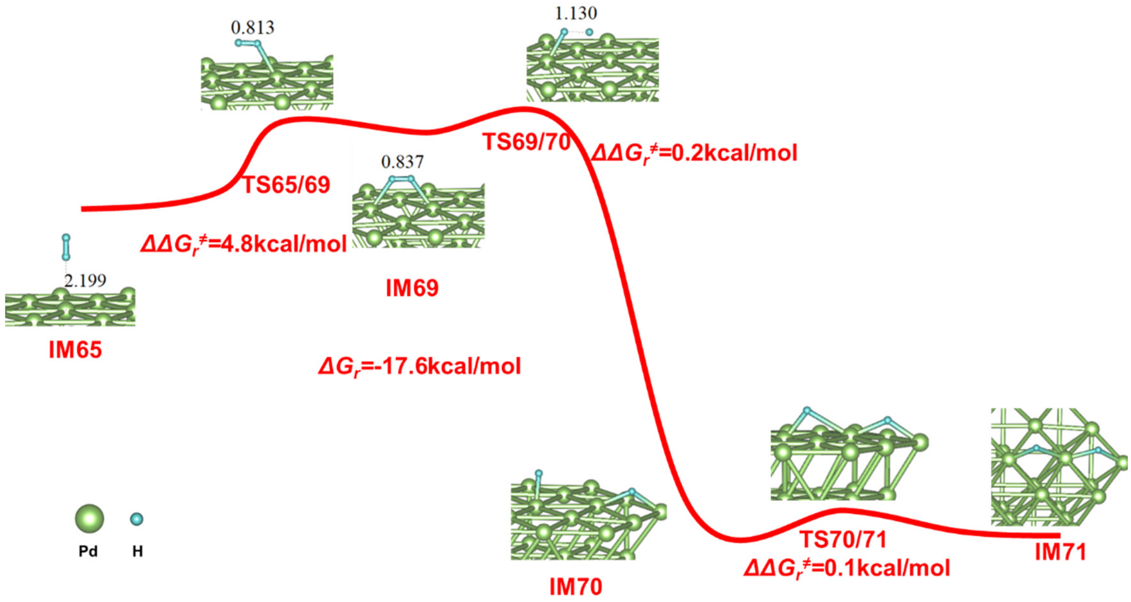

Figure S13. Simplified PES and selected structural information of alternative pathways for the dissociation of hydrogen on Pd(111) plane.

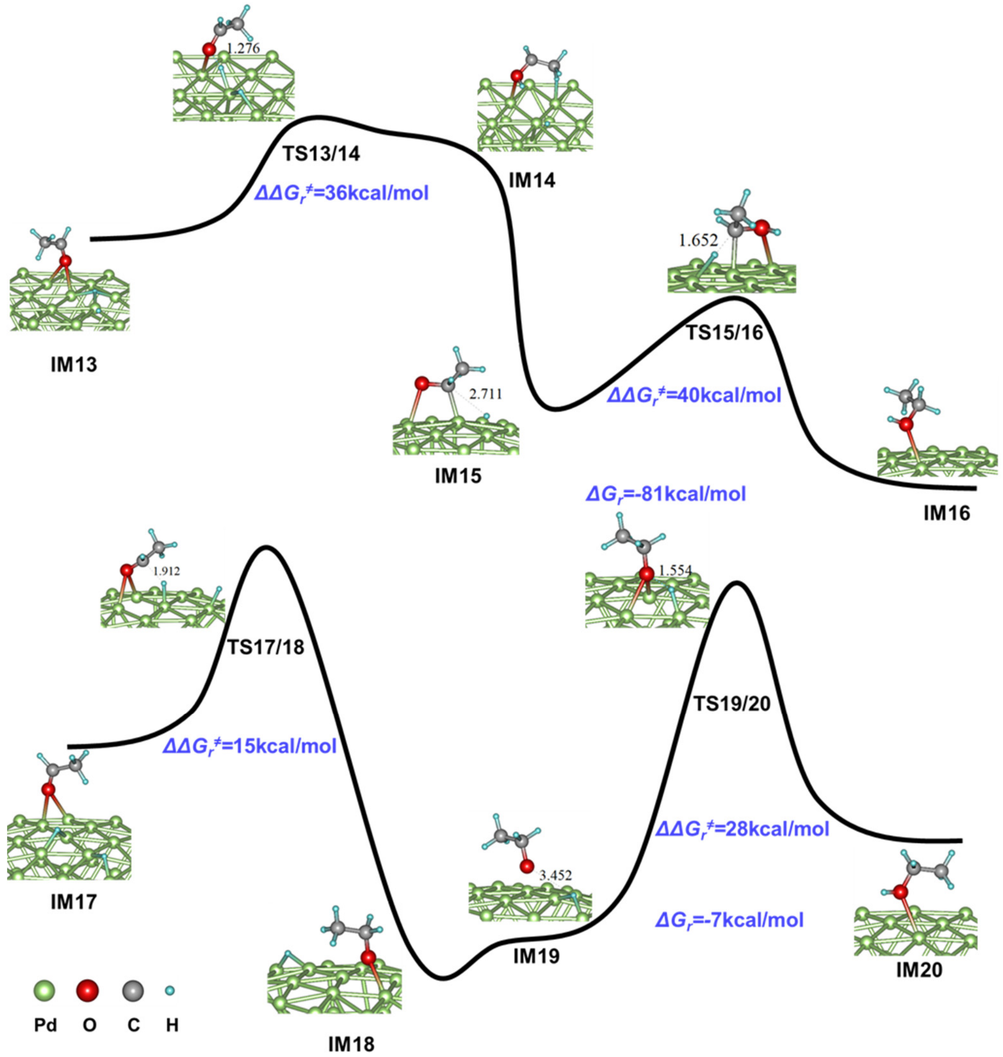

Figure S14. Simplified PES and selected structural information of alternative pathways

for the generation of ethanol mediated by the Pd(111) plane.

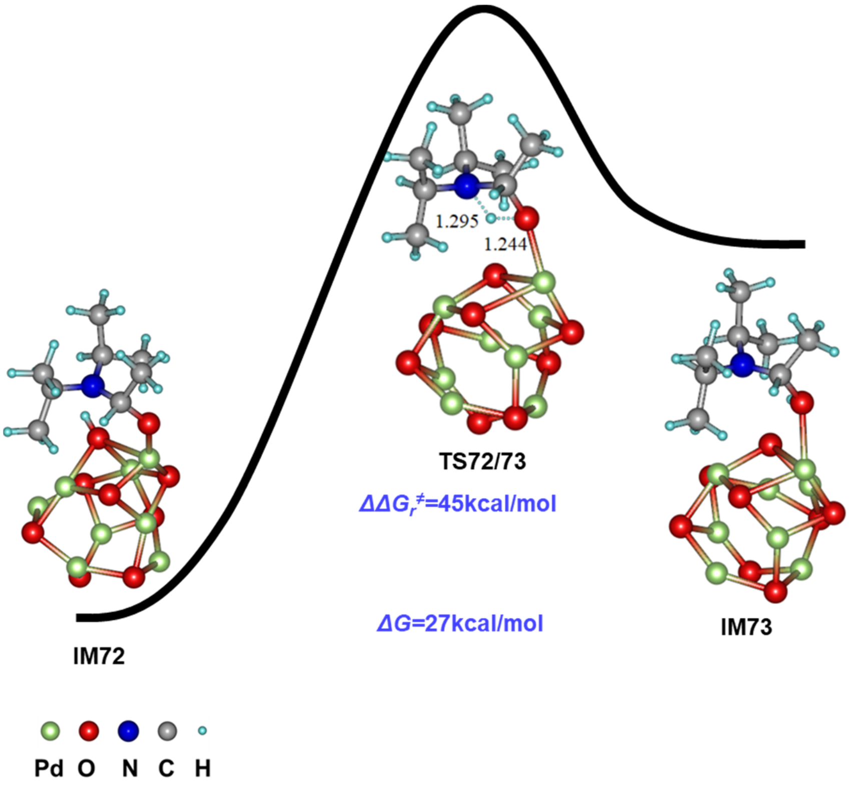

Figure S15. Simplified PES and selected structural information of alternative pathways for the hydrogen transfer process on Pd_8O_8 cluster.

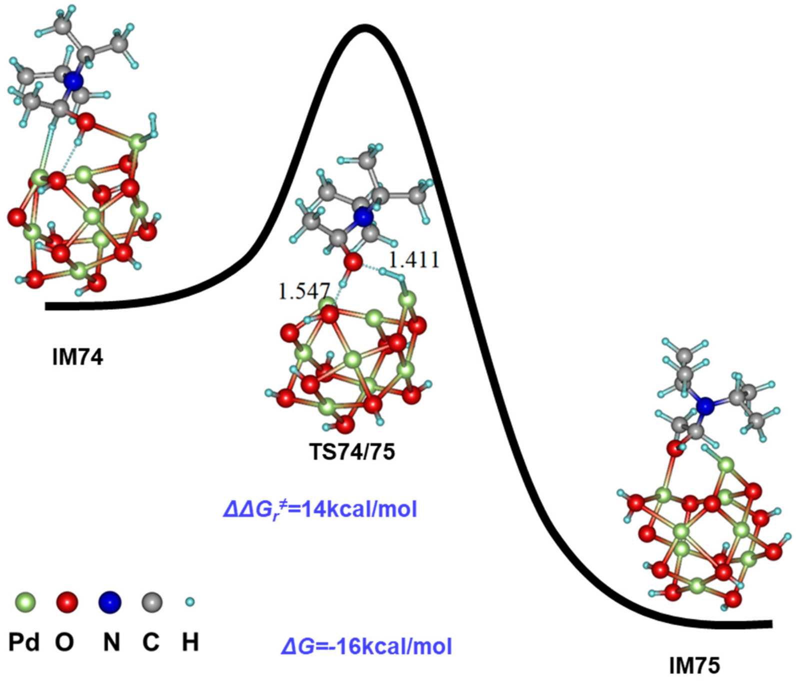

Figure S16. Simplified PES and selected structural information of alternative pathways for formation of water by hydrogen transfer process on $Pd_8O_{12}H_8$ cluster.

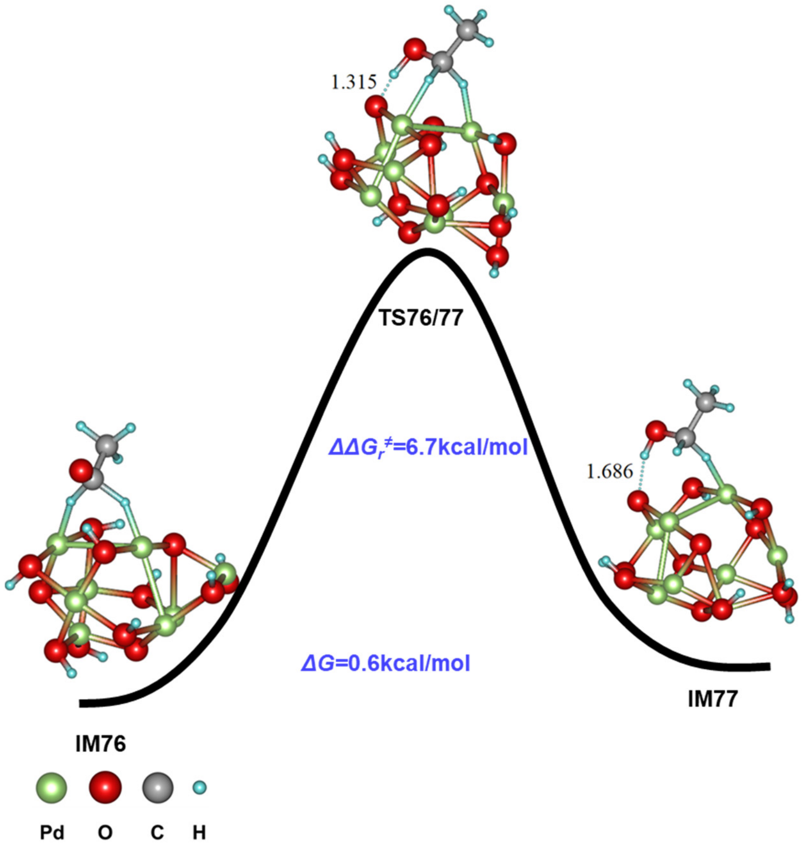

Figure S17. Simplified PES and selected structural information of alternative pathways for the generation of ethanol by hydrogen transfer process on $Pd_8O_{12}H_8$ cluster.

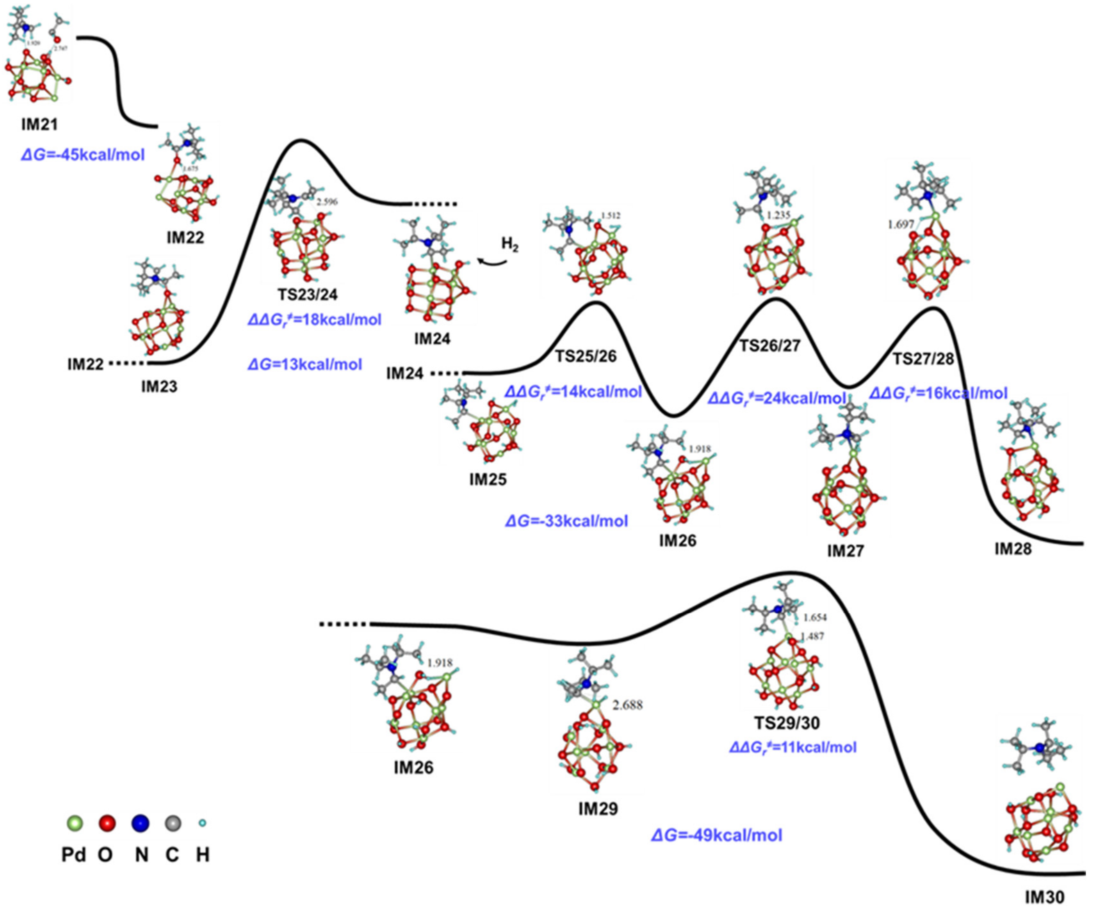

Figure S18. Simplified PES and selected structural information of alternative pathways for the generation of N,N-Diisopropylethylamine mediated by the $Pd_8O_{12}H_8$ cluster.

Cartesian coordinates for important structures of transition state as mentioned in the paper

TS1/2

Pd	-1	0.88543600	1.15465600	-3.36146400
Pd	-1	-3.29093400	-3.41120500	1.35833900
Pd	-1	-1.76063400	0.41217300	-3.21192100
Pd	-1	4.64460800	-1.18628100	0.90928000
Pd	-1	0.20169700	-1.51122500	-3.33540400
Pd	-1	-1.24447900	4.58434700	1.27973500
Pd	-1	-2.44290000	-2.25273600	-3.18551100
Pd	-1	-3.88957400	3.84306000	1.42987000
Pd	-1	3.96295000	-3.85225000	0.93483200
Pd	-1	-1.20311100	-1.12970500	-1.00196900
Pd	-1	-3.84844600	-1.86995800	-0.85144300
Pd	-1	-1.88554700	-3.79400800	-0.97553100
Pd	-1	2.84794100	-0.76934100	-3.48535900

Pd	-1	1.40049300	5.32589400	1.12968700
Pd	-1	-1.32817600	-5.33478700	1.23490000
Pd	-1	4.08699100	0.35439100	-1.30162900
Pd	-1	1.44333900	-0.38836100	-1.15262000
Pd	-1	3.40550600	-2.31165700	-1.27578700
Pd	-1	0.75424700	-3.04491200	-1.12607600
Pd	0	2.01863700	-1.95844900	1.20088200
Pd	0	-0.64916200	-2.72000400	1.35686000
Pd	-1	1.31763800	-4.59409700	1.08519700
Pd	-1	-1.07881900	3.07723600	-3.23791200
Pd	-1	5.32620700	1.47865400	0.88162100
Pd	-1	-5.25399400	-1.48709800	1.48221300
Pd	-1	0.16096000	4.20179000	-1.05396100
Pd	-1	-2.48409600	3.46033500	-0.90392200
Pd	-1	-0.52119000	1.53606800	-1.02817700
Pd	-1	-3.16621200	0.79510400	-0.87780800
Pd	0	-1.95529700	1.93748700	1.45154700
Pd	-1	-4.57172300	1.17789200	1.45599800
Pd	0	-2.65866900	-0.75772600	1.48324500
Pd	-1	2.12357000	2.27806900	-1.17793700
Pd	-1	3.36339100	3.40214500	1.00571000
Pd	0	0.71863700	2.69184300	1.30295300
Pd	0	2.73009800	0.73797100	1.16312400
Pd	0	-0.02509900	-0.04639200	1.30591700
C	0	3.58197100	-0.41068500	4.40324600
C	0	2.48979600	-0.70916200	5.42752900
H	0	3.33358600	-0.87154200	3.44009300
H	0	3.72135100	0.65806900	4.24726900
H	0	4.52299700	-0.83550400	4.75184400
C	0	2.82237200	-0.10052900	6.79061600
H	0	2.43432600	-1.80035800	5.53648100
H	0	2.12218200	-0.44284800	7.55097000
H	0	3.82367300	-0.41604000	7.08330400
H	0	2.80284400	0.98821200	6.75657400
C	0	1.07773500	1.17785200	4.52122400
C	0	0.02714500	-1.03259000	5.54948200
H	0	0.47604400	-1.96398700	5.91902200
C	0	-1.00230700	-1.41998200	4.49333400
H	0	-1.46159200	-0.54550200	4.02973200
H	0	-1.78671200	-2.02218100	4.95072300
C	0	-0.63098500	-0.33023900	6.74117100
H	0	-1.44942600	0.31282300	6.42321200
H	0	0.08575100	0.26560500	7.30209200
H	0	-1.04251500	-1.08913300	7.40711600

H	0	-0.52423900	-2.01385700	3.70492600
N	0	1.15338300	-0.28932300	4.92219900
C	0	-0.18212300	1.97589300	4.80020800
H	0	-0.28611100	2.22871200	5.85280600
H	0	-1.07287600	1.46486300	4.43966200
H	0	-0.07761700	2.90031900	4.22629000
O	0	1.23495300	0.93530800	3.15175300
H	0	1.20559600	-0.22093800	3.65515100
H	0	1.94073200	1.71228900	4.94834500

TS3/4

Pd	-0.72192400	0.08488900	-3.43795100
Pd	4.42169000	-0.14647400	2.04618900
Pd	1.62182800	-1.39751400	-2.90958400
Pd	-2.25056400	4.07741400	0.58227600
Pd	1.69168300	1.42701200	-2.85913400
Pd	-2.46505200	-3.95221500	0.43796500
Pd	3.89380500	-0.04942000	-2.45613700
Pd	-0.17065600	-5.26091500	0.92191000
Pd	0.11373400	5.24140300	1.10522300
Pd	1.83625900	-0.03279000	-0.45988000
Pd	4.07590200	-1.53971900	-0.29968800
Pd	4.15215900	1.35010300	-0.24732500
Pd	-0.56154700	2.75926600	-3.43469500
Pd	-4.64601300	-2.45784400	-0.12365600
Pd	4.35197600	2.52724100	2.04722300
Pd	-2.60100400	2.68050100	-1.78471600
Pd	-0.37590900	1.34817100	-0.93780700
Pd	-0.12120800	4.04160700	-1.18232600
Pd	2.06219100	2.78790000	-0.64340700
Pd	-0.13595400	2.68935500	1.50659700
Pd	2.07640000	1.27294200	2.06709400
Pd	2.27575600	3.93793000	1.59242500
Pd	-0.69284900	-2.59377700	-3.53382100
Pd	-4.51418300	2.71876200	-0.02790900
Pd	4.20773500	-2.81148600	1.94963100
Pd	-2.73455200	-2.46274000	-1.88720900
Pd	-0.33149500	-3.97354100	-1.32441700
Pd	-0.43885800	-1.28258300	-0.99135200
Pd	1.91483200	-2.85513500	-0.74299100
Pd	-0.28343700	-2.71508000	1.41345900
Pd	2.05779100	-4.09232500	1.45266200
Pd	2.00244900	-1.44156500	2.01980900
Pd	-2.79097400	0.11323000	-1.81771400

Pd	-4.65310300	0.13143000	-0.08166400
Pd	-2.51159100	-1.27830400	0.83936200
Pd	-2.45180000	1.39879900	0.92228700
Pd	-0.29822100	-0.01848700	1.71357500
C	-4.86564300	1.86575100	3.57934800
C	-5.33470700	0.50387100	4.08707900
H	-4.33172300	2.40604200	4.36024600
H	-4.20797000	1.75397500	2.70678700
H	-5.72961700	2.45533300	3.27262500
C	-6.00437000	-0.31896400	2.98802300
H	-6.08406100	0.66941300	4.87195100
H	-6.15079200	-1.34674000	3.31789200
H	-6.97418800	0.11976100	2.75610600
H	-5.40850600	-0.32673900	2.06373500
C	-3.23755500	-0.73234900	3.97697100
C	-4.15286100	-0.12880200	6.20315500
H	-5.00130900	0.50287800	6.49674100
C	-2.87929300	0.58413200	6.65642400
H	-1.98635200	-0.00106300	6.44825100
H	-2.93700000	0.75695700	7.73125600
C	-4.34283300	-1.48349100	6.89078000
H	-5.16152000	-2.02698300	6.42033400
H	-4.58938500	-1.31868500	7.93972400
H	-3.44306800	-2.09361200	6.84590600
H	-2.78811000	1.54506800	6.15183500
N	-4.23967000	-0.25596400	4.73283600
C	-2.42081900	-1.91933000	4.39867100
H	-3.09143500	-2.76760600	4.53244300
H	-1.86188600	-1.74973000	5.31446200
H	-1.71450900	-2.15980600	3.59495000
H	-3.47607000	-0.76724400	2.90219600
H	1.03258500	-0.06271000	2.77872600
O	-1.95783100	0.50272800	3.88751400
H	-2.34083600	1.30648500	3.49522400
H	-1.22902800	0.25216000	3.10800300

TS5/6

Pd	1.56142100	-0.16569700	-3.28567300
Pd	-4.65605900	1.01000600	1.24599600
Pd	-0.46576200	1.69512400	-3.20623700
Pd	1.42103500	-4.57040000	1.00720200
Pd	-1.06580600	-0.98660400	-3.33306700
Pd	3.21971900	3.47526100	1.38672500
Pd	-3.09075800	0.87380500	-3.25287900

Pd	1.19457100	5.33565500	1.46655600
Pd	-1.20468900	-5.39319400	0.95952600
Pd	-1.54585800	0.41972600	-1.02261700
Pd	-3.57298300	2.28290100	-0.94014800
Pd	-4.17168700	-0.39770900	-1.06764800
Pd	0.96126400	-2.84749100	-3.41251000
Pd	5.24518800	1.61542000	1.30700300
Pd	-5.25566900	-1.67221300	1.11933000
Pd	2.50330900	-3.29780800	-1.17969900
Pd	0.47634000	-1.43964600	-1.10056300
Pd	-0.12214700	-4.12091900	-1.22700000
Pd	-2.14461200	-2.25172400	-1.14690500
Pd	-0.60650700	-2.77330000	1.22193200
Pd	-2.67113900	-0.86895500	1.34986700
Pd	-3.23049200	-3.53312500	1.03987600
Pd	2.15962900	2.51675700	-3.15910300
Pd	4.04566700	-3.74833900	1.05277500
Pd	-4.05583400	3.69233500	1.37254000
Pd	3.70266400	2.06566500	-0.92603300
Pd	1.67738600	3.92618500	-0.84607900
Pd	1.07593800	1.24570400	-0.97243700
Pd	-0.94815900	3.10452600	-0.89297100
Pd	0.61023900	2.69968200	1.47961800
Pd	-1.43063800	4.51378600	1.41969700
Pd	-2.06568900	1.87452300	1.40109900
Pd	3.10235300	-0.61562900	-1.05311200
Pd	4.64527400	-1.06656400	1.17980500
Pd	2.64695100	0.80028900	1.39516400
Pd	2.04644600	-1.91506600	1.23849900
Pd	0.01287200	-0.02213000	1.52480500
C	2.07078400	-2.20968200	4.33433400
C	1.53436800	-1.00106500	5.10347400
H	1.51809000	-3.11576400	4.57646100
H	1.98261300	-2.03014900	3.24884800
H	3.12196900	-2.36543700	4.57458600
C	2.39961500	0.22734200	4.83882200
H	1.60870200	-1.22254500	6.18016300
H	1.97838900	1.11639200	5.30634400
H	3.38981700	0.05002300	5.25895000
H	2.52142400	0.41081700	3.76316900
C	-0.35064600	0.17625500	3.95127200
C	-0.76069200	-1.79577400	5.47572500
H	-0.08198300	-2.55808800	5.88077300
C	-1.69237700	-2.50943800	4.50149900

H	-2.46730700	-1.84977700	4.11068400
H	-2.17525300	-3.34005100	5.01617000
C	-1.52543100	-1.21298200	6.67352100
H	-0.87143400	-0.55932700	7.24985500
H	-1.85852100	-2.02900900	7.31558200
H	-2.40149700	-0.64837400	6.36123800
H	-1.11826200	-2.91037800	3.65898500
N	0.10148900	-0.78614100	4.84192800
C	-1.59908200	0.92917400	4.36718200
H	-1.44344700	1.38097300	5.35097100
H	-2.49078200	0.30695000	4.40141600
H	-1.78019700	1.73801500	3.64762500
H	0.44055900	0.89690700	3.73552700
H	-1.21635700	-0.58970800	2.40078300

TS7/8

Pd	-1	1.52392300	-1.05773100	-3.17605800
Pd	-1	-3.59898800	3.15942400	0.88803900
Pd	-1	0.65786900	1.55222500	-3.25188400
Pd	-1	-1.00071700	-4.67060100	1.11524600
Pd	-1	-1.16223300	-0.50573800	-3.40882100
Pd	-1	4.45615400	1.50306700	1.58642400
Pd	-1	-2.02766300	2.10433700	-3.48438600
Pd	-1	3.59021900	4.11309800	1.51091300
Pd	-1	-3.68589900	-4.11846100	0.88273900
Pd	-1	-1.03872900	1.05011300	-1.14343300
Pd	-1	-1.90180900	3.66179100	-1.21973500
Pd	-1	-3.72260600	1.60292200	-1.37651600
Pd	-1	-0.29507700	-3.11584900	-3.33318200
Pd	-1	5.32222800	-1.10717400	1.66266500
Pd	-1	-5.41783300	1.10161400	0.73150600
Pd	-1	0.69455700	-4.16939500	-0.99248000
Pd	-1	-0.17102900	-1.56042900	-1.06724800
Pd	-1	-1.99030600	-3.61716200	-1.22523100
Pd	-1	-2.85778000	-1.00702900	-1.30088100
Pd	0	-1.88258300	-2.09755100	1.16719200
Pd	0	-2.80075800	0.51001900	1.13599100
Pd	-1	-4.55181600	-1.50818500	0.80719600
Pd	-1	3.34251700	1.00032400	-3.01899600
Pd	-1	1.68428000	-5.22293500	1.34820100
Pd	-1	-1.78004000	5.21721800	1.04541900
Pd	-1	4.33235800	-0.05380500	-0.67833900
Pd	-1	3.46727900	2.55718600	-0.75312900
Pd	-1	1.64760200	0.49906800	-0.91131300

Pd	-1	0.78189500	3.10981600	-0.98613200
Pd	0	1.79564800	2.07270500	1.48109200
Pd	-1	0.90520200	4.66494100	1.27826400
Pd	0	-0.87837600	2.67704500	1.31561100
Pd	-1	2.51306600	-2.11169600	-0.83585600
Pd	-1	3.50321600	-3.16496900	1.50538100
Pd	0	2.66125100	-0.56735800	1.58307100
Pd	0	0.82242000	-2.64828400	1.42922300
Pd	0	-0.06293000	-0.00900200	1.31849700
C	0	-2.07319100	-1.26564000	4.61279600
H	0	-1.46856200	-1.74308000	3.82412300
C	0	-2.10357100	1.09905600	5.32674000
C	0	-0.12324800	0.21081700	4.26289100
N	0	-1.44055900	0.04498700	4.85890000
O	0	-2.58554200	2.15125500	2.84106900
H	0	-3.02296900	2.63858900	3.53708600
H	0	-1.58034500	2.04147400	5.34876200
H	0	-0.25487400	0.09198000	3.13389000
C	0	-3.44986200	1.10204700	5.96259100
H	0	-3.65945200	0.18447200	6.51035900
H	0	-3.50154200	1.94013000	6.65948500
H	0	-4.23769000	1.24754800	5.21897600
C	0	-2.01863100	-2.13832700	5.86639100
C	0	0.85511100	-0.87076400	4.72044800
C	0	0.48872500	1.58674400	4.49055300
C	0	-3.49323200	-1.15653600	4.06036600
H	0	-2.40477900	-3.12855600	5.62624200
H	0	-0.99572200	-2.23898900	6.22483000
H	0	-2.62390100	-1.71247300	6.66580600
H	0	-3.69768400	-2.05042300	3.46293800
H	0	-4.24982500	-1.09597800	4.83958800
H	0	-3.57201300	-0.28334100	3.40643400
H	0	1.81346600	-0.69631000	4.22323700
H	0	1.00679700	-0.82795300	5.79924500
H	0	0.52176400	-1.87006600	4.44293900
H	0	1.48171600	1.59249300	4.03100700
H	0	-0.10437600	2.36122000	4.00060200
H	0	0.59813900	1.81616400	5.55088500

TS9/10

Pd	-1	-1.15412700	1.46478200	-3.18540000
Pd	-1	1.85695300	-4.40646400	0.93907700
Pd	-1	-1.52262600	-1.26110700	-3.12623500
Pd	-1	2.96260300	3.77066300	0.76164600

Pd	-1	0.99898000	-0.22149900	-3.48707900
Pd	-1	-4.60172800	0.65169500	1.84415600
Pd	-1	0.63021000	-2.94722000	-3.42778100
Pd	-1	-4.97026500	-2.07403400	1.90330600
Pd	-1	5.11549600	2.08452900	0.45995400
Pd	-1	0.35144600	-1.47214800	-1.12248200
Pd	-1	-0.01721400	-4.19660500	-1.06390900
Pd	-1	2.50419900	-3.15716400	-1.42465300
Pd	-1	1.36729600	2.50429000	-3.54626400
Pd	-1	-4.23323900	3.37736600	1.78490700
Pd	-1	4.37842900	-3.36679400	0.57836200
Pd	-1	1.08846100	3.98051600	-1.24156000
Pd	-1	0.71985600	1.25510500	-1.18234900
Pd	-1	3.24138000	2.29450900	-1.54321400
Pd	-1	2.87297800	-0.43136300	-1.48390900
Pd	0	2.62646200	1.06925400	0.96463100
Pd	0	2.31865800	-1.67613600	1.02731000
Pd	-1	4.74692500	-0.64107100	0.51917300
Pd	-1	-3.67548900	0.42508900	-2.82463000
Pd	-1	0.80970300	5.45668100	1.06324400
Pd	-1	-0.66451000	-5.44612100	1.30003800
Pd	-1	-3.95446600	1.90112000	-0.51983600
Pd	-1	-4.32289600	-0.82456900	-0.46075400
Pd	-1	-1.80134600	0.21555300	-0.82164900
Pd	-1	-2.16990300	-2.51061800	-0.76238000
Pd	0	-2.48564200	-1.04456600	1.69475500
Pd	-1	-2.81738300	-3.76000700	1.60164100
Pd	0	-0.37173000	-2.77998100	1.45646100
Pd	-1	-1.43302200	2.94094400	-0.88068200
Pd	-1	-1.71177100	4.41708300	1.42416600
Pd	0	-2.11205500	1.72643500	1.64189900
Pd	0	0.44523400	2.79278300	1.25770100
Pd	0	0.05985200	0.00201600	1.32357300
H	0	0.88750500	0.21217400	2.76389700
H	0	0.11219100	-0.08396700	2.91334000
C	0	4.18883600	-1.51066100	3.89299200
C	0	4.35081300	-0.17635800	4.61986900
H	0	3.61505500	-2.21888000	4.48820000
H	0	3.65779100	-1.35867700	2.94581300
H	0	5.16882100	-1.93889800	3.68245500
C	0	5.28762800	0.76057000	3.86200900
H	0	4.80469100	-0.36415100	5.60441600
H	0	5.24881300	1.76911300	4.27212800
H	0	6.30574300	0.38328500	3.95693400

H	0	5.04328500	0.79975300	2.79201700
C	0	2.47079900	1.30037000	4.03016600
C	0	2.32114200	-0.20483800	6.02358800
H	0	2.98956300	-0.99169900	6.39736600
C	0	1.01967700	-0.88547600	5.60063000
H	0	0.23844700	-0.16619600	5.36365900
H	0	0.66973200	-1.51186600	6.42128800
C	0	2.11881300	0.79012000	7.17151400
H	0	1.32323100	1.50101200	6.95919500
H	0	3.04221100	1.33930700	7.35321500
H	0	1.85368000	0.24110100	8.07515300
H	0	1.19232100	-1.51673000	4.72948200
N	0	3.03192600	0.41980800	4.89709900
C	0	1.41898300	2.28543600	4.43165000
H	0	1.84188900	3.03923800	5.10488200
H	0	0.55673400	1.83448900	4.91794700
H	0	1.06624500	2.80411900	3.53004200
O	0	1.48612400	-3.02720000	2.80951200
H	0	1.59106900	-3.50026000	3.63301300
H	0	3.13584200	1.65625000	3.24835900

TS11/12

Pd	-1	-1.62417300	-0.01285000	3.07108400
Pd	-1	4.76703600	0.03771100	-1.36668300
Pd	-1	0.74721700	1.38143100	3.09152800
Pd	-1	-2.34696200	-4.14535700	-1.42804900
Pd	-1	0.76933500	-1.36952100	3.09144100
Pd	-1	-2.41268500	4.10755200	-1.42814500
Pd	-1	3.14073300	0.02491000	3.11177600
Pd	-1	-0.04119900	5.50212700	-1.40783000
Pd	-1	0.04627500	-5.50200800	-1.40782800
Pd	-1	1.57068600	0.01301800	0.85244100
Pd	-1	3.94287900	1.40704200	0.87238000
Pd	-1	3.96481600	-1.34432500	0.87246600
Pd	-1	-1.60216000	-2.76397600	3.07112700
Pd	-1	-4.78404400	2.71309700	-1.44854000
Pd	-1	4.78894700	-2.71335500	-1.36680400
Pd	-1	-3.17123700	-2.77642700	0.81120500
Pd	-1	-0.79951800	-1.38167900	0.83160500
Pd	-1	-0.77771900	-4.13297900	0.83168500
Pd	-1	1.59359000	-2.73871700	0.85187200
Pd	0	0.01136600	-2.78122100	-1.55224800
Pd	0	2.40975400	-1.43712500	-1.53944800
Pd	-1	2.41766200	-4.10777900	-1.38762700

Pd	-1	-1.64599700	2.73824400	3.07103300
Pd	-1	-4.74030800	-2.78887000	-1.44857200
Pd	-1	4.74542400	2.78893200	-1.36712400
Pd	-1	-3.21509600	2.72560400	0.81118500
Pd	-1	-0.84358500	4.12018700	0.83157400
Pd	-1	-0.82120500	1.36916300	0.83150300
Pd	-1	1.54963300	2.76343200	0.85204000
Pd	0	-0.03286900	2.78114100	-1.55245400
Pd	-1	2.35218700	4.14546200	-1.38745400
Pd	0	2.38679400	1.47538400	-1.53979500
Pd	-1	-3.19310200	-0.02532800	0.81119600
Pd	-1	-4.76217100	-0.03779300	-1.44848600
Pd	0	-2.42383200	1.37232800	-1.58576000
Pd	0	-2.40184500	-1.41074200	-1.58629300
Pd	0	-0.03524000	-0.00034100	-1.57799400
H	0	0.80584200	0.00710000	-2.85201300
O	0	2.32632200	0.01790100	-3.36995300
H	0	2.23186900	0.01655000	-4.32069400

TS13/14

Pd	-1	-1.70623900	-0.04360400	3.08184400
Pd	-1	4.77257500	0.14830000	-1.22502000
Pd	-1	0.63177500	1.40514600	3.14824800
Pd	-1	-2.24033100	-4.19892400	-1.42255100
Pd	-1	0.71773100	-1.34473400	3.15396900
Pd	-1	-2.49691400	4.05044300	-1.44037200
Pd	-1	3.05511000	0.10451800	3.22002900
Pd	-1	-0.15923300	5.49948800	-1.37455500
Pd	-1	0.18267000	-5.49969500	-1.35054900
Pd	-1	1.53382400	0.05159600	0.92883900
Pd	-1	3.87109600	1.50129800	0.99484100
Pd	-1	3.95664800	-1.24837100	1.00069800
Pd	-1	-1.61990300	-2.79349900	3.08812500
Pd	-1	-4.83463800	2.60133200	-1.50658800
Pd	-1	4.85801600	-2.60147800	-1.21868300
Pd	-1	-3.14188800	-2.84573900	0.79699800
Pd	-1	-0.80305600	-1.39692200	0.86327000
Pd	-1	-0.71894000	-4.14666400	0.86877400
Pd	-1	1.61921200	-2.69765200	0.93476600
Pd	0	0.08969100	-2.80989500	-1.51120400
Pd	0	2.46756400	-1.32504000	-1.41226200
Pd	-1	2.52044000	-4.05066800	-1.28471100
Pd	-1	-1.79145300	2.70608900	3.07591400
Pd	-1	-4.66354600	-2.89816800	-1.49446100

Pd	-1	4.68689500	2.89815200	-1.23065500
Pd	-1	-3.31271100	2.65400200	0.78456800
Pd	-1	-0.97544700	4.10278700	0.85069500
Pd	-1	-0.89251100	1.35401500	0.85517300
Pd	-1	1.44796900	2.80203700	0.92275300
Pd	0	-0.06945000	2.81127400	-1.51960800
Pd	-1	2.26382000	4.19865200	-1.30274000
Pd	0	2.38246400	1.49052100	-1.43838200
Pd	-1	-3.22734200	-0.09542400	0.79086100
Pd	-1	-4.74893000	-0.14847000	-1.50023000
Pd	0	-2.48280600	1.34467300	-1.63585400
Pd	0	-2.36668700	-1.48735800	-1.57973500
Pd	0	0.02708700	0.01501400	-1.57470000
C	0	2.03494400	-0.43206400	-4.64583700
C	0	3.02069800	0.65154300	-4.43323400
H	0	1.51502700	-0.50532800	-5.60482700
H	0	2.59223800	1.36241700	-3.68718600
H	0	3.93559200	0.26072200	-3.97170900
H	0	3.27010600	1.18796300	-5.34588800
O	0	1.57378500	-1.07388300	-3.63336400
H	0	0.72393900	-0.45737100	-2.90751400
H	0	-1.25888200	0.50236800	-2.46656600

TS15/16

Pd	-1.38929100	0.83119600	3.11521100
Pd	4.06094100	-2.49513200	-1.33354600
Pd	1.36080300	0.75374400	3.13919600
Pd	-4.18890600	-2.26424400	-1.40385100
Pd	-0.08049000	-1.58969700	3.12461400
Pd	0.13589200	4.76515800	-1.36079300
Pd	2.66934400	-1.66637500	3.14827000
Pd	2.88586100	4.68814500	-1.33738400
Pd	-2.88071900	-4.68430200	-1.39454800
Pd	1.33596500	-0.83296400	0.89123900
Pd	4.08595800	-0.90920600	0.91484400
Pd	2.64444500	-3.25225000	0.90040800
Pd	-2.83026800	-1.51247700	3.10117900
Pd	-2.61415800	4.84214600	-1.38459700
Pd	2.61928200	-4.83821600	-1.34750100
Pd	-4.16385500	-0.67811400	0.84423200
Pd	-1.41304200	-0.75595700	0.86806600
Pd	-2.85580000	-3.09826800	0.85330800
Pd	-0.10545300	-3.17556900	0.87687900
Pd	-1.48148800	-2.39785800	-1.51343600

Pd	1.32542500	-2.46169300	-1.51914400
Pd	-0.13068400	-4.76137900	-1.37112300
Pd	0.05251700	3.17407700	3.12956600
Pd	-5.49727900	0.15591800	-1.41305600
Pd	5.50245200	-0.15186800	-1.31888400
Pd	-1.28038200	4.00817100	0.87238500
Pd	1.46910200	3.93116700	0.89610900
Pd	0.02587700	1.59033400	0.88035900
Pd	2.77764800	1.51089700	0.90545900
Pd	1.48098200	2.40028400	-1.49703300
Pd	4.19405300	2.26800800	-1.32827100
Pd	2.82622600	-0.06231000	-1.47068200
Pd	-2.72190800	1.66542300	0.85839100
Pd	-4.05560700	2.49889400	-1.39853600
Pd	-1.33355600	2.47270700	-1.55273300
Pd	-2.81170500	0.07692700	-1.53615000
Pd	0.02036000	-0.02029600	-1.56538800
C	0.15264600	-0.16669900	-3.72846100
C	0.58937400	0.97745600	-4.62875200
H	-0.73310600	-0.67118900	-4.12590800
H	1.40989200	1.53474000	-4.16849600
H	0.93688300	0.57801400	-5.58466000
H	-0.23915700	1.66199400	-4.80549200
O	1.17521600	-1.15280900	-3.68942000
H	2.03746800	-0.71966900	-3.54917500
H	-0.63877600	0.82319700	-2.66828500

TS17/18

Pd	-0.25872900	-1.48175400	2.95882400
Pd	0.99585600	4.41882400	-1.38800400
Pd	-1.13249900	1.20245600	2.98229200
Pd	3.40323000	-3.16760200	-1.20506400
Pd	1.62597500	0.57389800	2.91580700
Pd	-4.49469600	-1.38113900	-1.03624800
Pd	0.75040200	3.11194300	2.97121200
Pd	-5.16428000	1.23049100	-1.03166400
Pd	5.16176600	-1.11181000	-1.21277900
Pd	0.38473400	1.55717900	0.62840100
Pd	-0.55531500	4.09816000	0.88589700
Pd	2.29702700	3.42895100	0.85482500
Pd	2.33176500	-2.00637200	3.05153900
Pd	-3.54274700	-3.90827700	-0.99825800
Pd	3.59137100	3.79568300	-1.35337500
Pd	1.85054000	-3.61679900	1.01922400

Pd	1.21959900	-0.98799900	0.56151800
Pd	3.84608700	-1.50024100	0.98783000
Pd	3.17648500	0.99872000	0.87027600
Pd	2.64611700	-0.62537000	-1.70079400
Pd	1.84162600	1.91959000	-1.76448500
Pd	4.48201600	1.38759700	-1.27108000
Pd	-2.81924300	-0.87228600	3.15028300
Pd	1.48713100	-5.04403100	-1.12345500
Pd	-1.63018100	4.94260400	-1.32873000
Pd	-3.15592600	-2.45414700	1.10571800
Pd	-4.02751600	0.29456800	1.11105300
Pd	-1.41899300	-0.39249000	0.65568100
Pd	-2.35780200	2.24571100	0.91238800
Pd	-2.70635700	0.57379100	-1.61426000
Pd	-3.46556200	3.14843300	-1.18496400
Pd	-0.84565600	2.50150900	-1.68171900
Pd	-0.66532700	-3.08277000	0.91510400
Pd	-1.04573600	-4.55798200	-1.12435900
Pd	-1.88367800	-2.01895500	-1.59777100
Pd	0.77885300	-2.61773700	-1.66279900
Pd	0.01410500	-0.02996300	-1.81515500
C	-1.50852700	-0.58658300	-4.42190400
C	-0.69461500	-1.72842500	-4.97154900
H	-1.46154300	0.34686100	-5.00183500
H	-0.55608400	-2.49479800	-4.20444200
H	-1.26038700	-2.16751000	-5.79713400
H	0.27136500	-1.39751300	-5.34517900
O	-2.46762100	-0.79538400	-3.65210600
H	-0.09826100	0.13112900	-3.34882600
H	3.20034900	-0.49473000	-3.09886400

TS19/20

Pd	0.33712800	-1.55140500	3.00920100
Pd	-0.80347600	4.51461100	-1.33415800
Pd	-1.49294700	0.60222700	3.02623200
Pd	4.36191600	-1.58309800	-1.27450800
Pd	1.28428100	1.10645100	2.98740000
Pd	-3.53839000	-2.99443900	-1.14683300
Pd	-0.52052800	3.10883900	3.01413100
Pd	-5.20606600	-0.88664100	-1.10552200
Pd	5.18630200	0.97710900	-1.26828800
Pd	-0.25027200	1.53363000	0.67739800
Pd	-2.07303700	3.53175600	0.93244500
Pd	0.78951600	4.03639300	0.91481800

Pd	2.95890700	-0.98902300	3.01480000
Pd	-1.71015700	-4.95526900	-1.12029500
Pd	1.86138400	4.88785900	-1.28396900
Pd	3.11252500	-2.63459900	0.96740300
Pd	1.45863800	-0.49887000	0.63096000
Pd	4.10392600	0.10223200	0.91675500
Pd	2.50268000	2.11387000	0.87606300
Pd	2.67107200	0.45934300	-1.69977100
Pd	0.92802100	2.48593900	-1.67480500
Pd	3.56636500	2.97076400	-1.26764900
Pd	-2.31702600	-1.95051000	3.10841800
Pd	3.34792000	-4.07342300	-1.17395800
Pd	-3.45414400	3.97208800	-1.22572000
Pd	-1.95699800	-3.54685400	1.04560100
Pd	-3.83588000	-1.31917200	1.04623900
Pd	-1.16399700	-0.96240400	0.69731600
Pd	-3.03747100	1.12796700	0.96935900
Pd	-2.67618000	-0.50792400	-1.61316000
Pd	-4.39813800	1.55894800	-1.13109500
Pd	-1.75116400	2.03689100	-1.60678700
Pd	0.59071400	-3.16898800	0.94810800
Pd	0.82590300	-4.57621800	-1.16119700
Pd	-0.89306300	-2.53552500	-1.59938900
Pd	1.75418600	-2.08810100	-1.66280500
Pd	0.07152600	-0.04777800	-1.84542600
C	-1.32405900	-0.13300500	-4.97120800
C	-0.56699900	-1.45480100	-5.13869400
H	-2.30827200	-0.20944100	-5.46568600
H	0.42675400	-1.36487100	-4.69276100
H	-1.10620300	-2.25115800	-4.61935600
H	-0.46568900	-1.71793300	-6.19173800
O	-1.47707600	0.13020200	-3.60781400
H	-0.75702100	0.67272600	-5.46930200
H	-1.78947400	1.54869200	-3.05564600

IM22

Pd	2.72097100	-0.19032200	-0.53508000
Pd	1.98146200	1.35294000	1.70770700
Pd	1.17043300	-3.16075500	-0.42919500
Pd	0.97738600	2.45036100	-0.97929400
Pd	-0.90966600	-1.94090700	-0.05303000
O	3.84616300	0.92341800	0.77558000
O	2.72271600	1.54133200	-1.73086300
O	0.09155200	1.70223500	2.45441900

O	-0.76462700	3.01440600	-0.10800100
O	2.75987500	-1.55694600	0.72204400
O	1.53904900	-1.16020200	-1.59439800
O	-0.73933900	-0.82056200	1.49498300
O	-1.24535000	0.27792700	-1.04814100
Pd	1.00899600	-1.32056300	1.17710000
Pd	-0.70855500	0.98358400	0.68906500
Pd	0.36745000	0.16238400	-2.06351500
H	4.38080400	0.35376500	1.32724700
H	3.45691600	2.08580300	-1.44453800
H	-2.71283700	-0.32243900	-0.50845700
O	2.03209400	-0.30556200	2.92200900
O	1.91462400	3.13195600	0.68707800
O	-1.25760100	-2.94403700	-1.61500300
O	-0.21595500	1.96004200	-2.79912600
H	2.87963600	-0.74730000	2.89415200
H	1.23521500	3.64321600	1.12959300
H	-1.01147800	-2.45672300	-2.40261400
H	-1.14631200	2.10457100	-2.63144900
C	-3.91699100	2.08349000	-1.13761200
C	-5.25510400	1.35744000	-0.91676000
H	-3.56896900	2.52137500	-0.20465500
H	-3.15794900	1.39479700	-1.49973500
H	-4.05269200	2.87684100	-1.86997600
C	-5.77852600	0.81357700	-2.24975100
H	-5.98939400	2.10010000	-0.57860400
H	-6.63529600	0.16662900	-2.08352700
H	-6.09271500	1.64685900	-2.87343400
H	-5.01187200	0.26454300	-2.78879400
C	-4.60445400	-0.91199800	-0.16338200
C	-5.22805600	0.88696400	1.49049600
H	-5.45496000	1.95315100	1.38197700
C	-3.90240800	0.78295600	2.25546600
H	-3.57266900	-0.24661700	2.33778900
H	-4.04384200	1.19096600	3.25333200
C	-6.37543100	0.29432800	2.32507200
H	-6.12832700	-0.68723400	2.71070900
H	-7.27290600	0.21730000	1.71791100
H	-6.58477300	0.94729900	3.16896600
H	-3.10498900	1.34471700	1.75332100
N	-5.23220500	0.34581000	0.13237700
C	-5.18719100	-2.09342200	0.61283700
H	-6.27097400	-2.07837100	0.57084500
H	-4.84981600	-2.06741500	1.64172100

H	-4.81307000	-3.00722200	0.16073100
O	-3.19537700	-0.95156200	0.06776800
H	-4.78662800	-1.10437900	-1.22668000
H	-1.43750100	3.15800500	-0.77241600
H	-0.03074000	1.05826200	3.15209600

TS23/24

Pd	-0.12287700	-0.72342900	-2.16912700
Pd	-0.77502500	2.28533000	-1.19060700
Pd	1.10843300	-2.15745900	0.00488700
Pd	-3.15542700	0.27421000	-0.51239800
Pd	-0.03787600	-0.75528400	2.25329200
O	-0.18357400	1.20376000	-2.82880000
O	-2.34922700	-0.98132100	-2.05156500
O	-1.27342700	3.20406800	0.51503900
O	-3.64802700	1.37957300	1.08491900
O	1.70401200	-0.58111400	-1.19972600
O	-0.11542400	-2.59087500	-1.45615200
O	0.33098600	1.22552800	2.05895600
O	-1.81801600	-0.17059400	2.62375600
Pd	1.07736400	0.64613500	0.33540000
Pd	-1.65562200	1.51355800	1.67712000
Pd	-1.74334900	-2.24011300	-0.39183000
H	0.70891500	1.48315300	-3.05287600
H	-2.90190400	-1.28479900	-2.77274600
H	2.17071600	-2.18155400	2.40494700
O	1.21161300	2.53726500	-0.74060200
O	-2.76996900	1.98723200	-1.48897800
O	-0.58336100	-2.78217000	1.39047200
O	-3.37106000	-1.47094300	0.52859400
H	1.68042400	2.41455100	-1.57024000
H	-3.15729100	2.65440500	-0.90429100
H	-0.77445500	-3.57558500	1.89076600
H	-3.11774400	-1.26253000	1.44916900
C	3.69929200	1.74972000	2.08693900
C	4.28431500	1.70599100	0.67975000
H	2.78141300	1.16333300	2.14906400
H	4.40808900	1.39902000	2.83703100
H	3.44699000	2.78570300	2.31048000
C	5.64133200	2.41024900	0.64808900
H	3.58201200	2.21706800	0.01013700
H	5.99306900	2.55783900	-0.37092200
H	5.54056100	3.38769300	1.11819900
H	6.38179000	1.83064000	1.19910400

C	4.38855700	-0.70086800	0.93558600
C	4.64654700	0.11789000	-1.30230200
H	4.11470500	-0.81136900	-1.53953200
C	6.14315200	-0.05512700	-1.58090700
H	6.61972000	-0.67837600	-0.82634400
H	6.64998900	0.90875800	-1.59963400
C	4.05056600	1.19332200	-2.19832600
H	4.46435700	2.18280200	-2.01530600
H	2.97049100	1.21072900	-2.07563500
H	4.26375100	0.91657800	-3.23085400
H	6.26892600	-0.52720800	-2.55460600
N	4.43264400	0.32803500	0.15152100
C	4.49519300	-2.10579500	0.48314800
H	4.51880900	-2.76637800	1.34666400
H	5.36847400	-2.28757900	-0.14104500
H	3.60173200	-2.35148800	-0.10906800
O	2.05287300	-1.47767000	1.76086700
H	4.25886600	-0.53269800	1.99591700
H	-4.02684100	0.77925000	1.73915600
H	-0.43559000	3.54197500	0.85578900

TS25/26

Pd	1.20771700	-1.19899500	1.86677600
Pd	1.12902400	1.90806200	1.68863800
Pd	-1.03133100	-2.63415500	0.57744200
Pd	3.13442600	0.67799000	-0.43253800
Pd	-0.69085800	-0.54124800	-1.91772200
O	1.51522500	0.43059900	3.03311000
O	3.13752100	-1.05883500	0.82780900
O	0.65614500	3.24867400	0.25576900
O	2.71347600	2.19303100	-1.67738300
O	-0.86222600	-1.14129300	1.91130300
O	0.96253700	-2.79174600	0.69637500
O	-1.28189400	1.28835900	-1.17009700
O	0.66190700	0.54771600	-2.69686000
Pd	-1.21517100	0.31884300	0.54740200
Pd	0.66086800	1.96007800	-1.38978100
Pd	1.92790900	-1.90996500	-0.77838400
H	0.84402600	0.46567800	3.72191000
H	3.94963000	-1.45495000	1.14664800
H	-1.48283300	-3.09302700	-0.91376500
O	-0.81679300	1.69702500	2.19134800
O	3.04857200	2.02871100	1.05502000
O	0.52711500	-2.23028100	-2.25719800

O	3.07081600	-0.70663500	-1.93351000
H	-0.84606800	1.09173800	2.94300600
H	3.06045300	2.86726700	0.57141600
H	0.97651100	-2.04832500	-3.09097800
H	2.47581800	-0.32164400	-2.60455100
C	-4.17317700	-1.44014200	1.27898900
C	-5.03049300	-0.28838400	0.76299700
H	-3.60329000	-1.87213600	0.45589400
H	-3.48424900	-1.11170100	2.05680000
H	-4.82202900	-2.21112600	1.69349700
C	-5.86870900	0.32989400	1.88616500
H	-5.71382100	-0.67763900	0.00169000
H	-6.44296400	1.17549500	1.50950900
H	-6.55950800	-0.41923100	2.27180200
H	-5.23652300	0.67275900	2.70459100
C	-3.29865500	1.41213000	0.82482200
C	-4.53342000	1.16386400	-1.26527100
H	-3.64041000	1.68343900	-1.63186500
C	-5.73327600	2.11384700	-1.28178100
H	-5.89512300	2.46594700	-2.30004200
H	-5.56097100	2.97576100	-0.63944900
C	-4.78460800	-0.03436400	-2.17624200
H	-5.78854100	-0.44137800	-2.05378800
H	-4.05651400	-0.81671700	-1.97626800
H	-4.67217700	0.28933300	-3.21059600
H	-6.63393900	1.60119200	-0.94376000
N	-4.22388500	0.76416400	0.11942800
C	-2.97434400	2.84591100	0.53565200
H	-3.88645400	3.43354100	0.66189500
H	-2.60127900	3.00779000	-0.47451200
H	-2.23511900	3.20702000	1.24629900
O	-2.28505900	-1.93534800	-1.46279300
H	-3.25080200	1.13399900	1.87474000
H	2.84358200	1.87309800	-2.57848200
H	-0.24512500	3.53923200	0.42758300
H	-2.45884800	-2.43929300	-2.26092300
H	-1.10375900	-3.80974300	-0.46375600

TS26/27

Pd	-0.37624600	2.12520300	0.52634600
Pd	-1.96080100	0.14330800	2.29302800
Pd	2.30258600	1.86205800	-0.52650700
Pd	-3.31673000	0.42637100	-0.57986600
Pd	0.13043000	-1.62835200	-1.29704300

O	-1.32289400	2.07240500	2.33592900
O	-2.20763600	2.24690300	-0.68738800
O	-2.41994800	-1.81019700	2.07907500
O	-3.91387700	-1.50331900	-0.47618100
O	1.32546800	1.26317400	1.26475900
O	0.50323100	2.14566000	-1.26610200
O	-0.04787000	-2.37071300	0.54714600
O	-1.57996300	-2.30673100	-1.76788700
Pd	0.64476800	-0.58936700	1.11915500
Pd	-2.02077700	-2.13639400	0.09541300
Pd	-0.96700600	1.10161800	-2.08809900
H	-0.63996500	2.14324700	3.01182200
H	-2.62188500	3.07482900	-0.93590400
H	2.08024700	-0.09029500	-1.36379000
O	-0.16367600	-0.39594600	3.05559600
O	-3.72543600	0.57475500	1.37575300
O	0.08892300	-0.41822800	-2.98093800
O	-2.74375700	0.23004300	-2.52170200
H	0.29344700	0.39298600	3.37316000
H	-4.25518100	-0.21602100	1.55058800
H	-0.49920700	-0.83932400	-3.61888100
H	-2.59257200	-0.71957900	-2.67630700
C	4.45076500	1.53618000	1.07167200
C	5.48309900	0.41521600	0.94679500
H	3.92366100	1.66912200	0.10512200
H	3.71953600	1.33330800	1.85181400
H	4.95895700	2.47077100	1.30408000
C	6.05798100	0.07193600	2.32641400
H	6.29941500	0.76605900	0.30491900
H	6.81508700	-0.70536100	2.23215600
H	6.51383600	0.95923400	2.76407900
H	5.27121900	-0.28395400	2.99052700
C	3.73687400	-1.33092600	0.77698100
C	5.55977100	-1.31563100	-0.88615300
H	5.01353300	-2.22805200	-1.15049800
C	7.02651900	-1.68260800	-0.65616100
H	7.41375500	-2.18633700	-1.54129400
H	7.11110600	-2.35604400	0.19609600
C	5.38755200	-0.31963000	-2.03641100
H	5.92488900	0.60919000	-1.84922000
H	4.32935400	-0.09526700	-2.17070800
H	5.76937300	-0.76339100	-2.95521500
H	7.63512700	-0.79865500	-0.46868500
N	4.94976300	-0.80406100	0.34037200

C	3.61453900	-2.83704300	0.92768500
H	4.30838400	-3.19173700	1.69317100
H	3.81914100	-3.36804900	-0.00051500
H	2.59551600	-3.07176900	1.23712500
O	2.05388700	-1.02555600	-1.07717600
H	3.37106000	-0.80726900	1.66282400
H	-4.03853400	-1.84228200	-1.37086100
H	-1.73410800	-2.27725400	2.57403900
H	2.87815600	-1.10495500	-0.16142900
H	2.94783700	2.30785600	-1.79449800

TS27/28

Pd	-0.47281900	0.76358100	1.46413300
Pd	1.69646600	2.41255400	0.00625400
Pd	-2.88637700	-0.64317100	0.54309900
Pd	3.01709000	-0.04637000	1.46820100
Pd	1.07703400	-1.82008700	-1.60064200
O	0.26263600	2.66308700	1.43021100
O	1.18479500	-0.06942600	2.60028800
O	2.99895000	2.02522300	-1.46973100
O	4.41648500	-0.13582700	0.01560300
O	-1.69368100	1.14721300	-0.11722100
O	-1.13766200	-1.15998500	1.46276200
O	1.19609200	-0.03230400	-2.52019300
O	2.97013100	-1.95121300	-1.47200000
Pd	-0.33715700	0.70710600	-1.44560800
Pd	2.95391900	-0.02004600	-1.47527700
Pd	0.75481200	-1.84877500	1.40866000
H	-0.43043800	3.21199500	1.04721700
H	1.19225500	-0.09766700	3.55838500
H	-0.82617600	-1.52593500	-0.52482800
O	0.27514100	2.66358700	-1.40947800
O	3.15189300	1.94677400	1.34439600
O	0.67333600	-3.23377400	-0.10527200
O	2.76932600	-2.05991800	1.49328500
H	-0.48301300	3.12762700	-1.03580100
H	3.96594800	2.08179000	0.83845800
H	1.40697400	-3.84824900	0.00384200
H	3.08719100	-2.40194800	0.63872500
C	-4.42530700	1.34824800	1.84270300
C	-5.50811100	0.79447000	0.91285900
H	-3.81648700	0.53452800	2.25335100
H	-3.77530400	2.04834800	1.31765800
H	-4.89154000	1.86671500	2.68041000

C	-6.45391300	1.93537800	0.53321600
H	-6.08099900	0.04813700	1.47750200
H	-7.26194300	1.60411400	-0.11341700
H	-6.89811600	2.32125500	1.45177800
H	-5.91825000	2.75101900	0.05029700
C	-4.42950000	0.93131300	-1.35661700
C	-5.65746100	-1.13354900	-0.68039400
H	-5.22090400	-1.41857700	-1.64664200
C	-7.16201300	-0.92078000	-0.86790600
H	-7.57830100	-1.83332100	-1.29645300
H	-7.38409000	-0.10186900	-1.54589600
C	-5.45511600	-2.29948400	0.29132800
H	-5.92487300	-2.10175800	1.25473300
H	-4.38765100	-2.48142700	0.45425500
H	-5.89370000	-3.20426700	-0.12861700
H	-7.66048300	-0.73854000	0.08322200
N	-4.87318000	0.05563800	-0.22664100
C	-5.43579200	1.31209300	-2.43906700
H	-6.30394100	1.84160400	-2.05584800
H	-5.76801900	0.43762200	-2.99660000
H	-4.91901300	1.97415000	-3.13544700
O	-0.95171300	-1.26692100	-1.45313400
H	-4.00524100	1.83171500	-0.90610400
H	4.75211800	-1.03830900	-0.05755400
H	2.53468500	2.29920200	-2.27231800
H	-3.61083000	0.40602700	-1.85641600
H	-2.48665100	-1.30211400	-0.72996800

TS29/30

Pd	0.65426800	0.43455100	-1.27816100
Pd	-1.73972400	2.37428900	-0.47123900
Pd	2.82371300	-0.46232300	-0.31786100
Pd	-2.83746600	-0.38246600	-1.65647200
Pd	-1.08754000	-1.46823000	1.83697500
O	-0.04023600	2.31745500	-1.62326800
O	-0.87683300	-0.57162800	-2.52188300
O	-3.30005600	2.26844100	0.77924900
O	-4.45988700	-0.22864100	-0.48695700
O	1.46981200	1.19588200	0.37280400
O	1.21668900	-1.43943200	-1.07123100
O	-1.69590600	0.34341500	2.48521800
O	-2.94861100	-1.65970200	1.46340100
Pd	-0.14006300	0.99638200	1.54707000
Pd	-3.24605400	0.22717700	1.16231500

Pd	-0.63190200	-2.15057700	-1.05488800
H	0.58155600	2.90388900	-1.17563300
H	-0.77666300	-0.72900100	-3.46225800
H	1.50644300	-0.93617000	1.29443800
O	-0.61123000	3.01745300	1.08430700
O	-2.94841200	1.59864600	-1.91512700
O	-0.58654500	-3.17946500	0.70204200
O	-2.62031200	-2.38290400	-1.27710200
H	0.23914500	3.35035700	0.77361500
H	-3.83132000	1.81261700	-1.58015100
H	-1.34938400	-3.76836400	0.73094100
H	-3.00455100	-2.51499400	-0.38914500
C	4.48731800	2.09718000	-0.82221000
C	5.71778900	1.60326600	-0.05619800
H	4.23002400	1.39619300	-1.61981700
H	3.62880800	2.21894000	-0.16087200
H	4.70560600	3.06449900	-1.27495000
C	5.93942400	2.42772600	1.21547300
H	6.59556800	1.72952400	-0.70093700
H	6.81640100	2.06079100	1.74781100
H	6.09720500	3.47279600	0.95068200
H	5.07595700	2.36299600	1.87675000
C	4.54720700	-0.31551700	0.98378900
C	6.55998500	-0.74583100	-0.38874700
H	6.30460600	-1.75257400	-0.03742600
C	8.01295400	-0.46345200	-0.00139500
H	8.65525900	-1.24638100	-0.40377600
H	8.10908100	-0.45171800	1.08389400
C	6.35577800	-0.71285700	-1.90694000
H	6.66160200	0.24193900	-2.33272700
H	5.30423000	-0.88266400	-2.14327600
H	6.94812500	-1.50304100	-2.36757200
H	8.35094800	0.49544300	-0.39309400
N	5.65584500	0.17990800	0.29443800
C	4.84436000	-1.31255500	2.09155400
H	5.47465900	-0.82708800	2.83997000
H	5.35450000	-2.20698600	1.73783600
H	3.90871400	-1.61092100	2.56435100
O	1.17546000	-1.50727600	2.01487900
H	3.98038000	0.51742500	1.41356100
H	-4.78945800	-1.11598700	-0.30009900
H	-2.99357800	2.70715900	1.58362700
H	1.18973900	-2.39435100	1.62258900
H	3.87441600	-1.46434500	0.00203600

TS31/32

C	0.09386200	0.19491700	0.39272100
O	-0.37627900	1.27918600	-0.17022700
C	1.42598200	-0.36088500	-0.10866700
H	1.63040700	-1.33960800	0.31980100
H	1.41752400	-0.41665000	-1.19449600
H	2.21195600	0.32701400	0.18902400
N	-1.08118300	-0.78839300	-0.07731200
H	-1.38095400	0.24213100	-0.53225400
H	-1.68301200	-1.18450800	0.64444500
H	-0.81427900	-1.52318700	-0.73339900
H	0.07780300	0.17588100	1.50555200

TS32/33

C	-0.23339200	0.31579800	0.44436900
O	1.14717000	-0.89832000	0.03834300
C	-1.42808700	-0.36134600	-0.15096600
H	-1.59925700	-1.33318400	0.30370200
H	-2.29834200	0.26578400	0.04285200
H	-1.30802300	-0.46283100	-1.22576500
N	0.41864400	1.20895700	-0.29260200
H	1.12113600	-1.80998700	-0.27940500
H	1.01431400	1.82618500	0.25074600
H	1.16990300	0.15595900	-0.64866100
H	-0.23872100	0.35522400	1.53758100

TS32/34

C	0.46291600	0.16309800	0.47030500
O	-1.42644400	-0.68650500	-0.06844500
C	-0.14355500	1.28993000	-0.12669400
H	-0.44904900	2.08717900	0.53207000
H	0.26425900	1.61717100	-1.07296400
H	-1.20656800	0.61396000	-0.36951400
N	1.38730000	-0.58809500	-0.15347700
H	-2.11687100	-1.36321200	-0.09359300
H	1.40952900	-0.51499200	-1.16034600
H	1.56018600	-1.51444700	0.20311100
H	0.32279800	-0.03512300	1.52146500

TS32/35

C	0.30040200	-0.09580100	0.47278600
O	-1.75285700	0.26713000	-0.11494000
C	0.95206600	1.12010300	-0.08897200

H	0.19508200	1.87791500	-0.26680500
H	1.64950000	1.51362400	0.65044300
N	0.66909100	-1.23968200	-0.07802200
H	-2.15950200	-0.58215400	-0.29079300
H	0.28766900	-2.11274500	0.25001500
H	1.19309600	-1.27766400	-0.94857500
H	-0.83763400	0.05224900	0.78536600
H	1.49619200	0.92369300	-1.01686700

TS36/37

C	0.09557300	-0.00000200	0.15402300
C	0.86839400	-1.26408400	-0.24147500
H	1.13602200	-1.25344300	-1.29558700
H	1.77526200	-1.30881200	0.35434500
H	0.27355500	-2.14508300	-0.01365400
O	-0.47021300	0.00002400	1.34264100
C	0.86850200	1.26401200	-0.24148100
H	1.13613400	1.25334300	-1.29559200
H	0.27373800	2.14506400	-0.01366900
H	1.77537300	1.30866800	0.35434000
N	-1.28628100	0.00004900	-0.68672900
H	-1.62583600	0.00006300	0.43113000
H	-1.48672900	-0.83191900	-1.24086900
H	-1.48667100	0.83202800	-1.24087300

TS37/38

C	-0.25755400	0.01066800	0.18655700
C	-1.32334500	-0.77018100	-0.52526000
H	-1.51906500	-0.35788400	-1.51079000
H	-1.02705400	-1.81154700	-0.62637700
H	-2.24184800	-0.72390200	0.06094800
O	1.41455200	-0.29824200	-0.73594000
C	-0.35012200	1.50435600	0.06075700
H	-1.26337100	1.83010000	0.55894500
H	0.49643300	1.97656700	0.54996200
H	-0.39833600	1.81245200	-0.97982900
N	0.37596100	-0.46174200	1.26077500
H	1.95693800	0.26366600	-1.30062700
H	0.18054900	-1.44270500	1.43961400
H	1.45373100	-0.39767200	0.53792300

TS37/39

C	-0.39954100	0.13697600	0.17091800
C	0.43832800	0.37531500	1.29371600

H	0.57460700	1.40765200	1.58484300
H	0.33659300	-0.30915900	2.12191500
H	1.46976300	0.02853800	0.65661300
O	1.56321800	-0.36547500	-0.65686100
C	-1.08806700	-1.17391500	-0.00465500
H	-2.01553900	-1.17679500	0.57082600
H	-1.32764400	-1.35657100	-1.04904600
H	-0.45126700	-1.97465500	0.35590900
N	-0.80133900	1.15384500	-0.61827500
H	2.19910600	-0.71044700	-1.29634500
H	-0.23812200	1.98996900	-0.58638900
H	-1.14818200	0.91809500	-1.53538900

TS40/41

C	2.28519800	0.40995400	0.37375100
C	1.48783100	-0.79731600	-0.09746000
H	2.49471400	1.07146500	-0.46302500
H	1.72542100	0.97004700	1.11662300
H	3.22662900	0.08995600	0.81193200
H	2.04445500	-1.32350900	-0.87912300
C	-0.92897700	0.23072800	0.31520100
N	0.18679500	-0.37611400	-0.62826400
C	-2.15342400	-0.68732200	0.29783100
H	-1.90234200	-1.68188200	0.66178600
H	-2.55929100	-0.74510800	-0.70966500
H	-2.91247400	-0.25568400	0.94357700
O	-1.02876500	1.40212700	-0.27520700
H	0.04007600	0.70424600	-1.05580200
H	-0.50191500	0.23880600	1.34311600
H	1.32506200	-1.49272000	0.73254000
H	-0.20155500	-1.09609400	-1.23839600

TS41/42

C	2.49158300	0.23129000	-0.24094500
C	1.41181700	-0.68677600	0.31765900
H	2.57079900	0.09955000	-1.31696500
H	2.24188100	1.26931900	-0.03287600
H	3.45340500	0.00518600	0.21275200
H	1.69848900	-1.73132100	0.14081900
C	-0.95363700	-0.36967100	0.37517500
N	0.14968000	-0.45397900	-0.35155700
C	-2.28030800	-0.60359100	-0.27086400
H	-2.49300200	-1.67239900	-0.23023600
H	-2.25567700	-0.29353500	-1.31142800

H	-3.07106600	-0.07795800	0.25720600
O	-0.77677700	1.52252600	0.17462900
H	-1.37793200	2.25592300	-0.01285100
H	-0.92250800	-0.55915700	1.45354200
H	1.32409500	-0.54242100	1.40536500
H	-0.01875400	0.81694000	-0.58761300

TS41/43

C	-0.78337000	-0.34430300	-0.39659800
O	-1.91154300	1.48946500	0.02328500
C	-1.78708200	-1.36965600	0.00332600
H	-2.75966300	-0.89306600	0.08401300
H	-1.85120800	-2.12482700	-0.78006600
N	0.31753200	-0.30084500	0.32221100
H	-1.28004600	2.14095600	0.32950300
H	-1.24032100	0.66745000	-0.78791500
H	-1.54452900	-1.86239900	0.94873000
C	1.42961100	0.57858300	0.04685700
H	1.19806400	1.13619000	-0.86245100
H	1.54900600	1.29008800	0.87275200
C	2.72031200	-0.21437300	-0.13509500
H	2.95772400	-0.77330500	0.76769100
H	2.61760000	-0.91437300	-0.96037500
H	3.54479500	0.45949200	-0.34946200
H	0.40137300	-0.83751300	1.18487600

TS41/44

C	2.05529600	0.70484100	-0.21738900
C	1.60819900	-0.67123200	0.26628200
H	2.29845100	0.68302000	-1.27713000
H	1.22996600	1.39875700	-0.06436300
H	2.92339200	1.04885500	0.33627100
H	2.29607000	-1.45245300	-0.07209100
C	-0.82294800	-0.60528800	0.40655500
N	0.28409700	-0.98165800	-0.23076700
C	-2.09841300	-0.54223100	-0.21475200
H	-2.17049100	-0.97569700	-1.20367400
H	-2.05143400	0.69484600	-0.32423100
H	-2.94649200	-0.75345000	0.41945600
O	-1.02544800	1.56930200	0.06738400
H	-0.86824700	2.50777900	0.22995400
H	-0.73319300	-0.45891700	1.47184300
H	1.56832600	-0.68952000	1.35721800
H	0.21575100	-1.00256900	-1.24113800

TS45/46

C	2.48813100	0.28871100	-1.03781400
C	1.50647200	-0.56667400	-0.23825600
H	2.22711400	0.28233400	-2.09367700
H	2.47170000	1.31749000	-0.68876800
H	3.49797900	-0.09879600	-0.92854200
C	1.96427000	-0.69471000	1.21552600
H	1.49475700	-1.57129600	-0.68055600
H	1.19380300	-1.13947700	1.83767000
H	2.84814500	-1.32708600	1.25851300
H	2.22372200	0.27471000	1.63155200
C	-0.18575400	1.32828200	0.41406900
C	-0.89178600	-1.03348000	-0.61806600
H	-0.51003300	-1.61284400	-1.47172900
C	-2.22399500	-0.39988600	-1.04466200
H	-2.96924300	-0.48393200	-0.25859100
H	-2.61434700	-0.90132100	-1.92651600
C	-1.09312400	-2.01060300	0.53926500
H	-1.40447400	-1.49344900	1.44119400
H	-0.18907300	-2.57754000	0.74765700
H	-1.87332700	-2.71718600	0.26477200
H	-2.08762500	0.65486700	-1.27572700
N	0.14646900	-0.00244500	-0.38281800
C	-1.18606400	1.21420400	1.55794600
H	-0.82362100	0.53787000	2.32858900
H	-2.16705600	0.91148700	1.21262500
H	-1.27629500	2.20971000	1.98719400
O	-0.51729500	2.06900200	-0.63628200
H	0.01371100	0.82832200	-1.21232500
H	0.78834000	1.61616900	0.86860000

TS46/47

C	1.89245300	0.10074300	-1.35970000
C	1.50613300	-0.75774500	-0.15477700
H	1.36711900	-0.22510100	-2.25380900
H	1.60162200	1.12959000	-1.15316200
H	2.96179200	0.05510400	-1.54294100
C	2.52285300	-0.60975100	0.97614200
H	1.48574100	-1.81755100	-0.43682500
H	2.13254000	-1.02457900	1.90295300
H	3.42638000	-1.15320400	0.71013700
H	2.79613000	0.42952200	1.13587100
C	-0.05705100	0.66111900	1.00014800

C	-0.92573700	-1.08091400	-0.49516100
H	-0.41791100	-1.73834200	-1.20784600
C	-1.78341200	-0.09329900	-1.29482800
H	-2.76584800	0.03715100	-0.85098200
H	-1.91855000	-0.44573100	-2.31377600
C	-1.74599400	-1.97935000	0.43403600
H	-2.25751000	-1.40180200	1.19747700
H	-1.09903300	-2.70121400	0.92881500
H	-2.48793200	-2.52091700	-0.14728300
H	-1.28329400	0.87713700	-1.31851300
N	0.14172000	-0.41621500	0.25702000
C	-1.28517700	1.28851000	1.37463500
H	-1.24452900	1.77615800	2.34034100
H	-2.20321600	0.74497900	1.21807100
H	-1.20839400	2.16934700	0.55954600
O	-0.19431300	2.42058700	-0.47154800
H	0.09947400	3.08785300	-1.10209800
H	0.83348200	1.09453900	1.42430200

TS46/48

C	1.76964000	-1.01100600	-1.17476300
C	1.18274900	-1.08399700	0.23490400
H	1.10568900	-1.46897100	-1.90411100
H	1.91096700	0.03368300	-1.44085200
H	2.72710300	-1.52289300	-1.20900900
C	2.22049700	-0.65379000	1.26661200
H	0.88600200	-2.11675400	0.46245700
H	1.75815700	-0.51395600	2.24045100
H	2.98340000	-1.42410800	1.34786900
H	2.69782700	0.27328900	0.96796500
C	0.08280600	0.97277800	0.70837200
C	-1.21910400	-0.91201000	-0.28960500
H	-0.87406000	-1.89303500	-0.63093200
C	-1.71705900	-0.14162700	-1.51904100
H	-2.03373200	-0.83628500	-2.29320600
H	-0.91524500	0.47671000	-1.91608000
C	-2.32626000	-1.16412500	0.73466600
H	-2.80870100	-0.24291700	1.04388400
H	-1.91952700	-1.65712600	1.61531500
H	-3.08098900	-1.81181200	0.29437600
H	-2.56516700	0.49576300	-1.28215100
N	-0.03868700	-0.27312000	0.31498900
C	-1.10553100	1.84004800	0.96326800
H	-1.85913500	1.83484200	0.17804800

H	-0.77888100	2.86563900	1.12026700
H	-1.57301700	1.49843300	1.88724800
O	1.17691100	2.04507600	-0.95196400
H	0.84506500	2.94158500	-0.91666800
H	1.02334200	1.54151800	0.29944300

TS49/50

C	2.00350300	1.80344700	-0.01532200
H	2.35321900	1.94273800	1.00760100
H	1.91468700	2.77710900	-0.49553300
H	2.72313800	1.21213300	-0.57842500
C	0.61137900	1.14575200	-0.05717700
H	-0.09293000	1.80775100	0.51201100
O	0.20379200	0.78294200	-1.24554000
N	0.62748900	-0.12502900	0.93213500
H	0.53435100	0.20479300	1.88553600
H	-0.40570800	-0.65179900	0.49099500
N	-1.34331900	-0.94048100	-0.33781800
H	-1.24078900	-1.85354000	-0.75643500
H	-0.87457100	-0.18400100	-0.97744000
C	1.77075900	-1.04264400	0.82147200
C	1.85035900	-1.68115400	-0.55912700
H	1.64367900	-1.81817700	1.58734600
H	2.69826000	-0.50197400	1.04381400
H	2.76783900	-2.26556600	-0.63392900
H	1.00133500	-2.34420100	-0.72617300
H	1.84860100	-0.91863000	-1.33636600
C	-2.73769400	-0.64250800	-0.01527000
C	-2.88195300	0.81735700	0.39205600
H	-3.06620900	-1.29962000	0.79977900
H	-3.36407900	-0.84737600	-0.89398900
H	-3.93075000	1.04716400	0.58018400
H	-2.31181600	1.02071300	1.29903500
H	-2.51591300	1.46602000	-0.40369200

TS50/51

C	2.00456000	-1.90705300	-0.25266700
H	2.73127100	-1.74078400	-1.05041800
H	1.74267200	-2.96330800	-0.22655500
H	2.45922600	-1.63733000	0.69909300
C	0.77090000	-1.08055900	-0.51123600
H	0.02832800	-1.55796000	-1.15127600
O	-0.18418500	-1.56618700	1.11327200
N	0.78299100	0.23125600	-0.57598800

H	-0.38059500	0.63446500	-0.00998700
H	-1.10343300	1.26184900	1.50268100
N	-1.26149900	0.59999200	0.75723000
H	-0.89448700	-0.47707300	1.04635100
H	-0.76125300	-2.29092800	0.85791600
C	1.89125800	0.93820500	0.07481400
C	1.66646800	2.43990600	-0.06638000
H	2.83863200	0.66488100	-0.40611400
H	1.94779800	0.66541100	1.13706400
H	2.50826200	2.98873600	0.35682400
H	1.56543900	2.70476300	-1.11923200
H	0.75636000	2.74161800	0.45425700
C	-2.59466800	0.71969900	0.17496100
C	-2.75948600	-0.27548800	-0.96590800
H	-2.73763300	1.74311800	-0.19514500
H	-3.34531200	0.52859000	0.95261600
H	-3.76309500	-0.19650200	-1.38362800
H	-2.03476800	-0.07343300	-1.75453900
H	-2.60856700	-1.29362500	-0.60629100

TS52/53

C	1.87436400	-2.38937300	-0.86979500
H	2.90933700	-2.05210500	-0.91263700
H	1.63608600	-2.91070900	-1.79569700
H	1.74398600	-3.08485800	-0.04135700
C	0.86853600	-1.22207500	-0.74153300
H	1.17391700	-0.40760700	-1.44816000
O	-0.37881800	-1.61801000	-0.83422600
N	1.05719300	-0.56851700	0.68441100
H	0.30217400	0.48702800	0.68174200
H	0.58579000	-1.22936800	1.29665400
N	-2.21644500	0.15158200	-0.70092000
H	-2.51245100	0.54928800	-1.58201200
H	-1.47142800	-0.61853400	-0.87716000
N	-0.54674600	1.38758100	0.72967600
H	-1.40356100	0.91225500	0.07042400
H	-0.87265400	1.47270500	1.68372100
C	2.40969500	-0.31527100	1.19273100
C	3.16767900	0.69455700	0.34040200
H	2.97193400	-1.25504500	1.24915200
H	2.30570900	0.07442700	2.21286100
H	4.15775200	0.85529000	0.76790300
H	3.28641900	0.33614300	-0.68115500
H	2.64328600	1.64928700	0.31564000

C	-0.12427600	2.69044900	0.21503300
C	0.14491000	2.61244600	-1.28232000
H	0.78123800	3.01439900	0.74479400
H	-0.91424100	3.42729500	0.41292300
H	0.49502400	3.57912800	-1.64441300
H	0.90528900	1.86219900	-1.49748600
H	-0.76451300	2.34598400	-1.82071600
C	-3.34448300	-0.46150900	0.00213200
C	-2.82611300	-1.23533700	1.20828600
H	-3.87574400	-1.14286500	-0.67686700
H	-4.04555300	0.32111400	0.31965000
H	-3.64355600	-1.77240000	1.68987400
H	-2.06668800	-1.95032600	0.88915400
H	-2.37688600	-0.55647900	1.93519600

TS53/54

C	-2.36700500	-1.81670600	1.04012700
H	-3.00216600	-2.07307900	0.19406700
H	-2.98647000	-1.38224300	1.82683300
H	-1.90709900	-2.72803700	1.41821500
C	-1.28739100	-0.82491700	0.66113900
H	-0.47944200	-0.77432600	1.39401000
O	-0.31705300	-1.97300700	-0.48673500
N	-1.51587100	0.30283700	0.03654200
H	-0.35313000	1.11307900	-0.24180200
N	1.74004500	-0.62632200	-0.85084000
H	2.10387600	-0.76443700	-1.78360200
H	0.78164800	-1.26646000	-0.69747600
N	0.63010900	1.60686500	-0.48916400
H	0.50472000	2.18070700	-1.31362000
H	1.33021800	0.47485300	-0.69835100
H	-0.82020400	-1.88596100	-1.30121500
C	-2.66973100	0.44198100	-0.84710600
C	-3.83329800	1.09863400	-0.10025000
H	-2.98503500	-0.52436800	-1.26255200
H	-2.37616000	1.08723600	-1.68482400
H	-4.66836700	1.27337000	-0.77989500
H	-4.17294600	0.46159600	0.71617000
H	-3.51204400	2.05296200	0.31817600
C	1.07549900	2.41604500	0.64005400
C	2.44766100	3.02143500	0.35652000
H	1.12735500	1.77078500	1.52356000
H	0.34833000	3.21573400	0.83956400
H	2.77881000	3.61485800	1.20884600

H	3.17891200	2.23360700	0.17241800
H	2.40421200	3.66514700	-0.52276700
C	2.71906600	-1.00763300	0.16576500
C	2.95667700	-2.51389500	0.12067400
H	2.31868400	-0.72037200	1.14357700
H	3.65583200	-0.46126100	-0.00044800
H	3.64296100	-2.80895300	0.91427900
H	2.01121700	-3.04044200	0.25346900
H	3.38386700	-2.80325000	-0.84006100

TS55/56

C	0.05531400	-0.46461600	0.21030700
C	-1.25197100	0.09116100	-0.13790500
H	-2.04864700	-0.64140600	-0.05768600
H	-1.42455200	0.89038700	0.59593200
H	-1.24576100	0.55822100	-1.12062100
N	1.17550000	0.03697600	-0.25662600
H	2.04851400	-0.19175400	0.19687100
H	0.11864300	-1.30996800	0.87963600
H	1.12608400	0.96580800	-0.66002400
H	0.37716100	1.71060700	1.52786300

TS55/57

C	-0.11992600	0.35721600	-0.24123200
N	-1.19515800	-0.42092700	0.12448000
C	1.23590100	-0.22436500	0.04250900
H	1.46566000	-0.92662200	-0.75817100
H	1.22405200	-0.76778400	0.98234600
H	2.00173100	0.54453600	0.06911100
H	-0.76566900	0.69186900	0.75342700
H	-0.05184300	1.83405400	0.80362800
H	-2.04953500	-0.18529500	-0.37240500
H	-0.15414000	0.95862500	-1.15695200

TS58/59

C	0.00423900	0.09461100	0.11472500
N	0.06834800	1.42387100	-0.19847500
C	-1.33790700	-0.54650600	-0.10256700
H	-1.38556400	-0.87023400	-1.14218300
H	-2.13278400	0.17212200	0.07130900
H	-1.47449800	-1.40900900	0.54209500
H	-0.06141400	1.01209500	1.03146100
H	-0.00912400	0.11007100	2.00611700
H	1.01872100	1.76157700	-0.32470300

C	1.18821300	-0.81767600	-0.07431100
H	1.28285200	-1.01540500	-1.14315700
H	2.10706100	-0.35147400	0.27368300
H	1.04904100	-1.75941700	0.44762300

TS60/61

C	2.26389700	0.45675800	-0.12537600
C	1.24345200	-0.63040400	0.21508600
H	2.36664300	0.54206800	-1.20430100
H	1.94320600	1.41800800	0.27354600
H	3.23281700	0.21197200	0.30190500
H	1.60014000	-1.59073300	-0.16896500
C	-1.01956900	0.17522900	0.34760300
N	-0.02656400	-0.38733200	-0.41775900
C	-2.42712900	-0.00362700	-0.14207600
H	-2.75309500	-1.00311300	0.14397000
H	-2.46568500	0.07442500	-1.22424800
H	-3.09588600	0.72676100	0.30259700
H	-0.91442700	0.14901500	1.44079700
H	1.14391400	-0.71100300	1.31082000
H	-0.32661700	0.91156700	-0.33914800
H	-0.90896900	1.99462300	0.31591700

TS62/63

C	1.85513600	0.02239600	-1.37622900
C	1.40972400	-0.93104700	-0.25116900
H	1.84461000	1.05858400	-1.02282700
H	2.86226900	-0.23592100	-1.72424500
H	1.17656200	-0.03865100	-2.23397800
C	2.47061600	-1.02296600	0.85341800
H	1.29130100	-1.94100500	-0.66695900
H	2.11249200	-1.61685800	1.70051200
H	3.36399000	-1.50775000	0.44669800
H	2.77530800	-0.03509300	1.21509000
C	-0.06948300	0.29593500	1.32883900
C	-1.07485900	-0.90460300	-0.63009000
H	-0.62169600	-1.51166800	-1.42196600
C	-1.72807900	0.31531400	-1.32080700
H	-2.17423200	0.00368100	-2.27179600
H	-0.98571000	1.09473000	-1.51174000
C	-2.10117500	-1.79141300	0.09551200
H	-2.53004100	-1.27466800	0.95816400
H	-1.62635700	-2.71089700	0.45233000
H	-2.91722400	-2.06100400	-0.58478800

H	-2.52634000	0.75050000	-0.71313800
N	0.05970900	-0.56066200	0.26163100
C	-1.15322600	0.98823900	1.77199100
H	-2.13709200	0.92763900	1.32944900
H	-1.06654500	1.56177600	2.68709200
H	0.85304700	0.42854700	1.88262600
H	-0.17582986	-0.55125761	2.35979374
H	-1.19895054	0.04387885	2.76248900

TS63/64

C	1.84732600	-1.15141700	-0.93904100
C	1.47975500	-0.46997800	0.37865200
H	1.73085200	-0.44253700	-1.76067900
H	2.88309300	-1.50498200	-0.89005900
H	1.20511500	-2.01376700	-1.13940900
C	2.52209200	0.56019400	0.82435000
H	1.38524100	-1.22361900	1.17162900
H	2.16708900	1.13692000	1.68737100
H	3.43818000	0.03948300	1.12287900
H	2.77150900	1.25705700	0.02181200
C	-0.02180200	1.19341000	-0.83362100
C	-1.02619200	-0.91176000	0.29458300
H	-0.52008600	-1.83699400	0.59769200
C	-1.63709800	-1.10741800	-1.08806900
H	-2.31132800	-1.97319600	-1.04253000
H	-0.87321800	-1.27626200	-1.84532400
C	-2.06881300	-0.55996400	1.36103700
H	-1.61511000	-0.47182700	2.35586100
H	-2.82657000	-1.34871400	1.40804700
H	-2.56920700	0.38298500	1.12390400
H	-2.20182300	-0.22906400	-1.40043300
N	0.09238900	0.18312000	0.29930700
C	-1.10154000	2.18748100	-0.46129000
H	-2.12732400	1.82268300	-0.62935800
H	-0.98992700	3.08217000	-1.08568300
H	0.95074700	1.69988600	-0.86049700
H	-0.75207812	1.15005075	0.41001142
H	-1.08072600	2.53762300	0.60878600

TS65/66

Pd	-1	-0.04532300	1.58825800	3.06784000
Pd	-1	0.13578600	-4.76293300	-1.42434900
Pd	-1	1.39758700	-0.75413100	3.06800400
Pd	-1	-4.19294100	2.26423500	-1.42484200

Pd	-1	-1.35243700	-0.83253200	3.06797800
Pd	-1	4.05712800	2.49943800	-1.42476600
Pd	-1	0.09047200	-3.17492100	3.06814300
Pd	-1	5.50003800	0.15704900	-1.42460200
Pd	-1	-5.50005400	-0.15655500	-1.42470300
Pd	-1	0.04523200	-1.58733700	0.82174500
Pd	-1	1.48814100	-3.92972600	0.82190900
Pd	-1	-1.26188200	-4.00812700	0.82188400
Pd	-1	-2.79534600	1.50985700	3.06781500
Pd	-1	2.61421900	4.84182800	-1.42493000
Pd	-1	-2.61423600	-4.84133300	-1.42437500
Pd	-1	-2.84058600	3.09744100	0.82141700
Pd	-1	-1.39767700	0.75505300	0.82158100
Pd	-1	-4.14770000	0.67665100	0.82155600
Pd	-1	-2.70479100	-1.66573800	0.82171900
Pd	0	-2.79268900	-0.08091700	-1.56111100
Pd	0	-1.32773000	-2.45760000	-1.56303000
Pd	-1	-4.05714500	-2.49894400	-1.42453900
Pd	-1	2.70470000	1.66665900	3.06786500
Pd	-1	-2.88582600	4.68502500	-1.42498100
Pd	-1	2.88580900	-4.68453100	-1.42432400
Pd	-1	2.65946000	3.25424400	0.82146800
Pd	-1	4.10236900	0.91185500	0.82163100
Pd	-1	1.35234600	0.83345300	0.82160600
Pd	-1	2.79525400	-1.50893600	0.82177000
Pd	0	2.79289900	0.07828700	-1.56118300
Pd	-1	4.19292400	-2.26374100	-1.42446300
Pd	0	1.46569100	-2.37805900	-1.56343700
Pd	-1	-0.09056300	3.17584200	0.82144200
Pd	-1	-0.13580300	4.76342700	-1.42495500
Pd	0	1.32578400	2.45635700	-1.56412800
Pd	0	-1.46354100	2.37671500	-1.56428400
Pd	0	0.00051300	-0.00768900	-1.64147400
H	0	-0.02118200	0.41692200	-3.66280800
H	0	0.01751900	-0.32139400	-3.37044400

TS66/67

Pd	-1	-0.06688700	1.58882800	3.06716300
Pd	-1	0.20008200	-4.76034800	-1.42318400
Pd	-1	1.40781900	-0.73352700	3.06786100
Pd	-1	-4.22313900	2.20783100	-1.42616100
Pd	-1	-1.34128100	-0.84904200	3.06784600
Pd	-1	4.02301800	2.55422700	-1.42614300
Pd	-1	0.13301300	-3.17171900	3.06878500

Pd	-1	5.49743500	0.23155100	-1.42497000
Pd	-1	-5.49739500	-0.23051200	-1.42509000
Pd	-1	0.06604400	-1.58274900	0.82312400
Pd	-1	1.54094900	-3.90837600	0.82275800
Pd	-1	-1.20780400	-4.02375600	0.82278800
Pd	-1	-2.81549300	1.47419400	3.06686800
Pd	-1	2.54854000	4.87694900	-1.42685300
Pd	-1	-2.54859100	-4.87597500	-1.42323000
Pd	-1	-2.88220400	3.05994700	0.81990100
Pd	-1	-1.40823000	0.73520700	0.82092900
Pd	-1	-4.15645700	0.62178300	0.82076400
Pd	-1	-2.68204400	-1.70082900	0.82181100
Pd	0	-2.80876700	-0.12006800	-1.56663500
Pd	0	-1.30158100	-2.48783000	-1.57055900
Pd	-1	-4.02296500	-2.55314900	-1.42395800
Pd	-1	2.68208000	1.70462900	3.06696500
Pd	-1	-2.94889300	4.64601600	-1.42690400
Pd	-1	2.94876500	-4.64487600	-1.42320100
Pd	-1	2.61510200	3.29091200	0.82008800
Pd	-1	4.08967100	0.96820800	0.82087800
Pd	-1	1.34179000	0.85098400	0.82068100
Pd	-1	2.81560600	-1.46961000	0.82181200
Pd	0	2.80917400	0.11593900	-1.56674400
Pd	-1	4.22314000	-2.20671900	-1.42389200
Pd	0	1.50589300	-2.36992500	-1.57013300
Pd	-1	-0.13372300	3.17527600	0.82005000
Pd	-1	-0.20010900	4.76148200	-1.42694300
Pd	0	1.29729000	2.48111300	-1.57073300
Pd	0	-1.50093100	2.36350900	-1.57061000
Pd	0	0.00118700	-0.02180300	-1.61324600
H	0	-0.02134200	0.60803100	-3.00667400
H	0	0.01667400	-0.50558500	-3.05999100

TS67/68

Pd	-1	-1.28568300	0.92720800	3.07407700
Pd	-1	3.86007800	-2.79439900	-1.42216800
Pd	-1	1.45097200	0.64439900	3.07345900
Pd	-1	-4.34955100	-1.94517700	-1.42134700
Pd	-1	-0.16274100	-1.58389300	3.07164000
Pd	-1	0.49085200	4.73984700	-1.41517200
Pd	-1	2.57378300	-1.86717900	3.07124600
Pd	-1	3.22738400	4.45681200	-1.41545500
Pd	-1	-3.22633500	-4.45662700	-1.42365600
Pd	-1	1.28690400	-0.93330500	0.82587700

Pd	-1	4.02367000	-1.21651600	0.82549400
Pd	-1	2.41035300	-3.44493500	0.82342200
Pd	-1	-2.89936700	-1.30091000	3.07190100
Pd	-1	-2.24564500	5.02297000	-1.41477300
Pd	-1	2.24671900	-5.02279600	-1.42430500
Pd	-1	-4.18597400	-0.36749600	0.82643300
Pd	-1	-1.44998200	-0.65007400	0.82591400
Pd	-1	-3.06270300	-2.87887600	0.82415200
Pd	-1	-0.32630700	-3.16177800	0.82380600
Pd	0	-1.65869200	-2.24365700	-1.58092000
Pd	0	1.16992800	-2.53430800	-1.58522900
Pd	-1	-0.48982500	-4.73965300	-1.42395500
Pd	-1	0.32778100	3.15569800	3.07603100
Pd	-1	-5.47257800	0.56629300	-1.41890900
Pd	-1	5.47356600	-0.56612700	-1.42022100
Pd	-1	-0.95904900	4.08937400	0.83066600
Pd	-1	1.77759000	3.80620900	0.83027900
Pd	-1	0.16557100	1.57713400	0.82857200
Pd	-1	2.90048700	1.29495800	0.82793800
Pd	0	1.65460000	2.27744500	-1.60693300
Pd	-1	4.35049100	1.94537300	-1.41767700
Pd	0	2.79088400	-0.28023100	-1.55937600
Pd	-1	-2.57273200	1.86071400	0.82851000
Pd	-1	-3.85911800	2.79465500	-1.41697200
Pd	0	-1.16417300	2.53992800	-1.58498900
Pd	0	-2.79442100	0.29001700	-1.56147600
Pd	0	-0.02113600	0.00304300	-1.74437100
H	0	0.37738700	1.48526000	-2.39689400
H	0	-0.17513600	-1.67571500	-2.27269300

TS65/69

Pd	-1	1.58035900	0.14602600	3.06917500
Pd	-1	-4.74517700	-0.43632700	-1.42502000
Pd	-1	-0.66560800	-1.44343300	3.06765700
Pd	-1	1.99239000	4.33069900	-1.42161700
Pd	-1	-0.91858500	1.29657700	3.06916200
Pd	-1	2.75196800	-3.88769100	-1.42493400
Pd	-1	-3.16439700	-0.29230000	3.06792900
Pd	-1	0.50605500	-5.47676600	-1.42640100
Pd	-1	-0.50667700	5.48109800	-1.42137100
Pd	-1	-1.58241600	-0.14472900	0.82212200
Pd	-1	-3.82825400	-1.73402300	0.82087400
Pd	-1	-4.08150600	1.00525500	0.82211300
Pd	-1	1.32718000	2.88555200	3.07032400

Pd	-1	4.99780400	-2.29869600	-1.42389600
Pd	-1	-4.99840400	2.30306900	-1.42376900
Pd	-1	2.90930800	3.03293500	0.82454000
Pd	-1	0.66345300	1.44427400	0.82327800
Pd	-1	0.41023400	4.18335400	0.82454200
Pd	-1	-1.83559500	2.59449500	0.82333000
Pd	0	-0.25442300	2.76502700	-1.60405000
Pd	0	-2.54242800	1.15428500	-1.55272800
Pd	-1	-2.75259900	3.89212300	-1.42274100
Pd	-1	1.83350700	-2.59356000	3.06770100
Pd	-1	4.49144800	3.18010500	-1.42144500
Pd	-1	-4.49195400	-3.17583100	-1.42625900
Pd	-1	3.41565500	-2.44616900	0.82194800
Pd	-1	1.16993900	-4.03503300	0.82062800
Pd	-1	0.91645900	-1.29752300	0.82107200
Pd	-1	-1.32897700	-2.88467300	0.82080100
Pd	0	0.25371200	-2.77477800	-1.56756300
Pd	-1	-1.99294500	-4.32616300	-1.42609700
Pd	0	-2.27174200	-1.61521900	-1.56509800
Pd	-1	3.16264800	0.29353300	0.82316800
Pd	-1	4.74458700	0.44073900	-1.42282200
Pd	0	2.53738100	-1.17272500	-1.56822400
Pd	0	2.28866700	1.59897600	-1.55151100
Pd	0	0.01397700	-0.04428000	-1.64430000
H	0	-0.16537200	1.59419500	-3.25609900
H	0	-0.06674300	0.78831000	-3.20767300

TS69/70

Pd	-1	1.58613100	0.03353500	3.06934600
Pd	-1	-4.76437300	-0.09964200	-1.42542000
Pd	-1	-0.76655900	-1.39363400	3.06737500
Pd	-1	2.29345900	4.17849300	-1.42067100
Pd	-1	-0.82492100	1.35796800	3.06917500
Pd	-1	2.46999800	-4.07265000	-1.42478500
Pd	-1	-3.17745500	-0.06802900	3.06769900
Pd	-1	0.11727700	-5.49892300	-1.42661100
Pd	-1	-0.11803000	5.50273100	-1.42081600
Pd	-1	-1.58918000	-0.03251200	0.82202400
Pd	-1	-3.94162600	-1.45897500	0.82059700
Pd	-1	-4.00041100	1.29124200	0.82202800
Pd	-1	1.52707500	2.78392800	3.07060900
Pd	-1	4.82261500	-2.64646400	-1.42372300
Pd	-1	-4.82316600	2.65079800	-1.42412500
Pd	-1	3.11601000	2.81873900	0.82528300

Pd	-1	0.76330400	1.39333500	0.82361000
Pd	-1	0.70589300	4.14583100	0.82311000
Pd	-1	-1.64752000	2.71708200	0.82373200
Pd	0	-0.05507300	2.76597000	-1.59689600
Pd	0	-2.46005700	1.32867500	-1.55897900
Pd	-1	-2.47063000	4.07670400	-1.42240000
Pd	-1	1.64522200	-2.71716600	3.06756600
Pd	-1	4.70507400	2.85438100	-1.42107700
Pd	-1	-4.70538400	-2.85030100	-1.42667500
Pd	-1	3.23392400	-2.68170300	0.82205400
Pd	-1	0.88176200	-4.10786000	0.82035900
Pd	-1	0.82297000	-1.36099400	0.81994900
Pd	-1	-1.52982400	-2.78388500	0.82048700
Pd	0	0.05530400	-2.77842800	-1.56800900
Pd	-1	-2.29405500	-4.17437100	-1.42628900
Pd	0	-2.38137900	-1.44830600	-1.56740600
Pd	-1	3.17516900	0.06888500	0.82351200
Pd	-1	4.76372400	0.10402000	-1.42252800
Pd	0	2.44965200	-1.34743600	-1.56996100
Pd	0	2.40303600	1.43123500	-1.56048100
Pd	0	0.01492400	-0.04388300	-1.64275600
H	0	-0.09762300	1.98102900	-2.97326100
H	0	-0.03490500	0.85300800	-2.95645200

TS70/71

Pd	-1	-1.58378900	0.02210400	3.06981400
Pd	-1	4.76552900	-0.07475100	-1.42710600
Pd	-1	0.81879300	1.36384500	3.06662900
Pd	-1	-2.44038900	-4.09823000	-1.41797500
Pd	-1	0.77774000	-1.38915600	3.07009500
Pd	-1	-2.32239600	4.15414300	-1.42589600
Pd	-1	3.18024200	-0.04704000	3.06668900
Pd	-1	0.07990100	5.49550500	-1.42967600
Pd	-1	-0.07821200	-5.50798300	-1.41754400
Pd	-1	1.59013900	-0.02785100	0.82207200
Pd	-1	3.99287200	1.31428000	0.81851900
Pd	-1	3.95281500	-1.43525100	0.82184600
Pd	-1	-1.62394300	-2.72924300	3.07266500
Pd	-1	-4.72434500	2.81294400	-1.42330700
Pd	-1	4.72630700	-2.82539100	-1.42444800
Pd	-1	-3.21346600	-2.70875100	0.82751100
Pd	-1	-0.80940600	-1.36926700	0.82536400
Pd	-1	-0.85060800	-4.11834400	0.82787900
Pd	-1	1.55214500	-2.77707900	0.82371100

Pd	0	-0.04626500	-2.66432800	-1.67946300
Pd	0	2.41162000	-1.40422600	-1.57880400
Pd	-1	2.32405500	-4.16681600	-1.42134600
Pd	-1	-1.54389300	2.77264400	3.06695900
Pd	-1	-4.80320300	-2.68850200	-1.41748100
Pd	-1	4.80484000	2.67636500	-1.42976900
Pd	-1	-3.13455300	2.79252300	0.82176500
Pd	-1	-0.73307700	4.13390700	0.81827900
Pd	-1	-0.77281400	1.38749300	0.81875900
Pd	-1	1.62973000	2.72473100	0.81827300
Pd	0	0.04528000	2.79116900	-1.57015900
Pd	-1	2.44231700	4.08553800	-1.42924800
Pd	0	2.42489600	1.36728500	-1.57000000
Pd	-1	-3.17366800	0.04216200	0.82473200
Pd	-1	-4.76364300	0.06201700	-1.42059600
Pd	0	-2.40561000	1.43825400	-1.56780800
Pd	0	-2.46113600	-1.34357100	-1.57406000
Pd	0	-0.04032800	0.04969400	-1.54605100
H	0	0.05903700	-4.19643900	-2.33753000
H	0	0.19485800	-0.90143500	-2.76049200

TS72/73

Pd	0.46821600	-0.39670600	1.41999700
Pd	-2.05450400	-2.25772400	0.41165200
Pd	1.51444100	-0.07816400	-1.92109900
Pd	-2.02084300	-0.10694300	1.69777900
Pd	-0.20699800	1.53943900	-0.97285100
O	-1.04410600	-1.71965000	2.13707400
O	-0.67134700	0.97557300	2.57211800
O	-2.56395500	-1.57290300	-1.44626500
O	-3.47402600	0.25950100	0.68936200
O	0.75169000	-1.65724200	-0.16566200
O	1.14508100	1.15501000	0.34377600
O	-0.18660200	0.29226400	-2.64279200
O	-1.98910600	2.33132100	-0.83000800
Pd	-0.63641200	-1.27693800	-1.41854400
Pd	-2.83732700	0.47439300	-1.19427300
Pd	-1.41507700	2.25975900	1.12628100
C	3.38393300	-2.18957200	1.41060700
C	4.62176000	-1.30280600	1.49768800
H	2.51227400	-1.63752200	1.77608800
H	3.18160500	-2.50370100	0.38735500
H	3.51549700	-3.07376600	2.03288800
C	5.89563300	-2.10614700	1.22265000

H	4.68394500	-0.89803500	2.51528700
H	6.75717100	-1.45286800	1.09682800
H	6.08259700	-2.76317300	2.07191900
H	5.79498000	-2.72620200	0.33222400
C	4.72240500	-0.40253000	-0.87513000
C	4.84234300	1.16321900	1.21597400
H	4.36552800	1.12374900	2.20558000
C	4.21133800	2.33873700	0.47312400
H	4.57526100	2.42913700	-0.54742800
H	4.44424400	3.25893300	1.00893600
C	6.34308700	1.35566600	1.43678300
H	6.88782400	1.45885000	0.50133400
H	6.76407900	0.52560100	2.00175700
H	6.48955000	2.26741000	2.01652500
H	3.12793600	2.22012300	0.44518000
N	4.46757900	-0.13435400	0.58817600
C	5.92794200	0.19823600	-1.57090500
H	6.85018300	-0.18419000	-1.13822200
H	5.92199800	1.28382600	-1.53433000
H	5.87793400	-0.10536600	-2.61683200
O	3.49937500	0.12780600	-1.32041200
H	3.39043600	0.12543800	-0.08137700
H	4.74841600	-1.49734300	-1.00457200

TS74/75

Pd	1.12225000	-2.09494000	-0.89076500
Pd	2.99385700	-0.87168600	1.29453400
Pd	-1.80144200	-1.87397800	-0.39170500
Pd	2.86763500	0.98795500	-1.30089000
Pd	-0.92386700	1.30127500	0.34608000
O	2.75010400	-2.58440200	0.20791700
O	2.14931300	-0.77418800	-2.29390900
O	3.01775000	0.85702300	2.31752700
O	3.15891900	2.60260800	-0.13768500
O	-0.21448400	-2.51502700	0.60351400
O	-0.51971200	-1.51018500	-1.91121600
O	0.03327100	1.06415100	2.09076900
O	0.32357900	2.73461700	0.09166700
Pd	0.06260500	-0.82292900	1.59842100
Pd	1.68328300	1.95981400	1.20470100
Pd	0.23683100	0.26787400	-2.23290500
H	2.48638500	-3.28136000	0.81878300
H	2.53867800	-1.07365600	-3.11730500
H	-2.96515700	1.00434800	-1.91053400

O	1.71495000	-1.61177700	2.68259300
O	4.15896800	0.02849400	-0.11226900
O	-1.47564000	1.38145100	-1.73155100
O	1.41472600	1.89940400	-2.42114500
H	1.62398800	-2.56758300	2.60142900
H	4.57287500	0.74752000	0.38659900
H	-1.26030900	2.28594600	-1.98816000
H	1.05079600	2.60320700	-1.85543500
C	-3.51636100	-0.30328600	2.06721400
C	-4.88910000	0.26530100	1.69479400
H	-3.36513100	-1.26444500	1.56451000
H	-2.71473200	0.38084900	1.76708500
H	-3.44401200	-0.45683100	3.14317500
C	-5.08988500	1.63894400	2.34565500
H	-5.64673500	-0.41647800	2.09992000
H	-6.06335500	2.04990500	2.08350100
H	-5.04225000	1.52883000	3.42924900
H	-4.31452400	2.34097600	2.04028500
C	-4.37975100	1.27064300	-0.53685400
C	-6.30844600	-0.26914600	-0.31335700
H	-6.26986400	-0.06442100	-1.39087200
C	-7.57044500	0.38153000	0.26717500
H	-8.44257900	0.01093900	-0.27213900
H	-7.53480300	1.46432800	0.16103900
C	-6.35823300	-1.78899700	-0.12677900
H	-6.45091500	-2.05705900	0.92576200
H	-5.44840600	-2.24077300	-0.52106500
H	-7.21432900	-2.19716800	-0.66380900
H	-7.69199700	0.13759900	1.32237000
N	-5.05596400	0.25452500	0.24070300
C	-5.21329600	2.49560300	-0.92643200
H	-5.61935200	2.98269700	-0.04343700
H	-6.02595000	2.20914300	-1.59050000
H	-4.56806600	3.19760900	-1.45372900
O	-3.91680600	0.66675600	-1.74837700
H	-3.49838700	1.60419900	0.04255300
H	2.77913900	3.37473100	-0.57288800
H	2.57129500	0.64553000	3.14796700
H	-3.43898500	-0.61786600	-1.41457500
H	-3.11099100	-1.42301400	-1.21956600

TS76/77

Pd	0.06073500	-1.75338600	0.64316200
Pd	-1.73901700	-1.32434200	-1.56218700

Pd	3.12455400	-1.08406800	0.80810400
Pd	-2.28561500	0.26174300	1.06183700
Pd	2.14000600	1.25982300	-0.07496500
O	-1.30584800	-2.72310000	-0.28227600
O	-1.00950800	1.25931700	2.33220100
O	-1.82745100	0.33518500	-2.71368200
O	-2.26629100	1.95111200	0.05253800
O	2.47576900	-2.49019200	-0.58899300
O	1.35230200	-0.70806400	1.55278500
O	0.99951600	1.01389600	-1.65809800
O	-0.36712500	3.82182700	-0.68149400
Pd	1.45260400	-0.86571000	-1.43412200
Pd	-0.85812400	1.56561600	-1.31351000
Pd	-0.58657000	2.66319400	0.86439300
H	-2.21690700	-3.02853000	0.61515500
H	-0.22141500	0.75160100	2.56948700
H	-1.25830400	0.17978300	-3.47736300
H	0.55832300	4.08632900	-0.74534600
O	-0.05133200	-1.85105500	-2.57807500
O	-3.29759000	-0.64785300	-0.40949700
O	3.64901200	0.77771900	1.33018700
O	1.38410400	2.96047300	1.19160400
H	0.05781200	-2.80355100	-2.47002700
H	-3.72516400	0.04962000	-0.92572000
H	3.36509800	1.01865800	2.21712500
H	1.70683800	2.90245600	2.09426800
H	-0.98129300	-2.15541200	2.08625000
H	2.17119700	-3.39271600	-0.48231100
C	-2.11839500	-2.18489100	2.33392400
C	-2.25315300	-2.36736800	3.83540600
H	-1.86431700	-3.34650600	4.11222500
H	-1.70606000	-1.59293400	4.36999400
H	-3.30701600	-2.32039500	4.10697800
O	-2.74560800	-3.09733400	1.59865000
H	-2.46341500	-1.10234600	2.08173000

NMR

N,N-Diisopropylethylamine : ^{1}H NMR (400 MHz, Chloroform-*d*) δ 3.06 – 2.96 (m, *J* = 6.5 Hz, 2H), 2.46 (q, *J* = 7.2 Hz, 2H), 1.00 (d, *J* = 6.6 Hz, 15H); ^{13}C NMR (101 MHz, Chloroform-*d*) δ 48.53, 39.12, 20.62, 17.05.

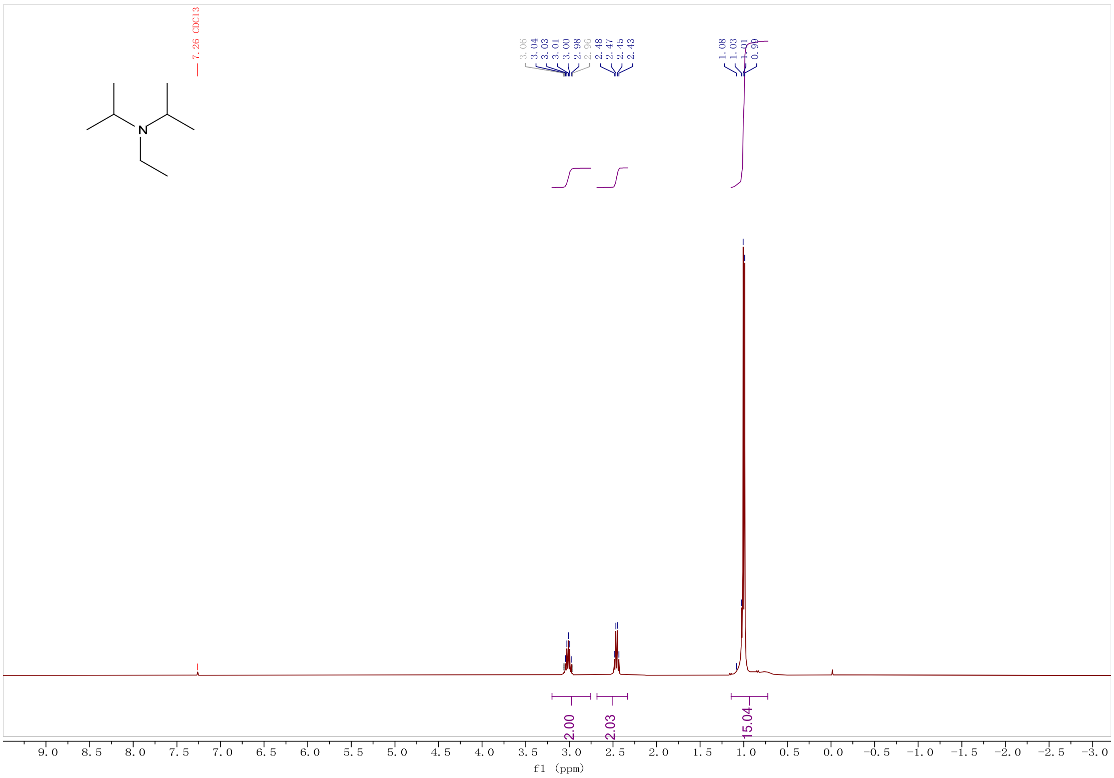

Figure S19. The ^{1}H-NMR spectrum of N,N-Diisopropylethylamine

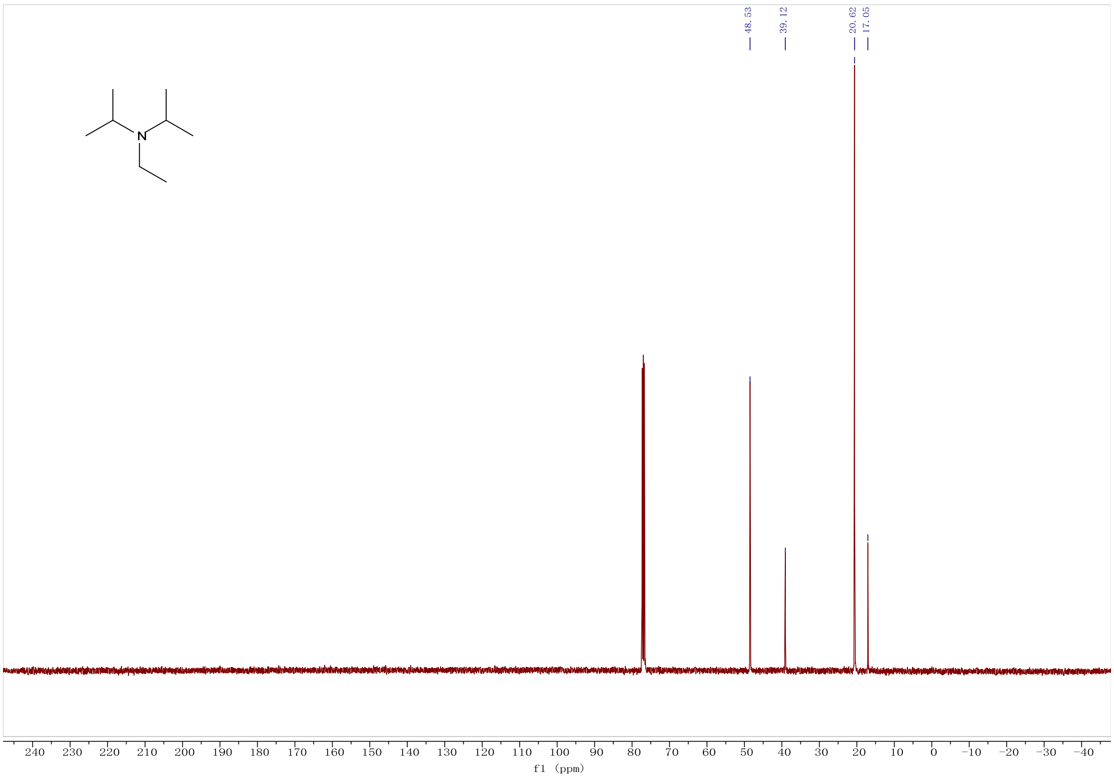

Figure S20. The ^{13}C-NMR spectrum of N,N-Diisopropylethylamine

1,2,2,6,6-pentamethylenepiperidine:^{1}H NMR (400 MHz, Chloroform-*d*) δ 2.22 (s, 3H), 1.51 (tdt, *J* = 8.2, 4.6, 2.1 Hz, 2H), 1.46 – 1.34 (m, 4H), 1.03 (s, 12H); ^{13}C NMR (101 MHz, Chloroform-*d*) δ 53.77, 41.20, 28.50 (d, *J* = 2.1 Hz), 26.30, 17.90.

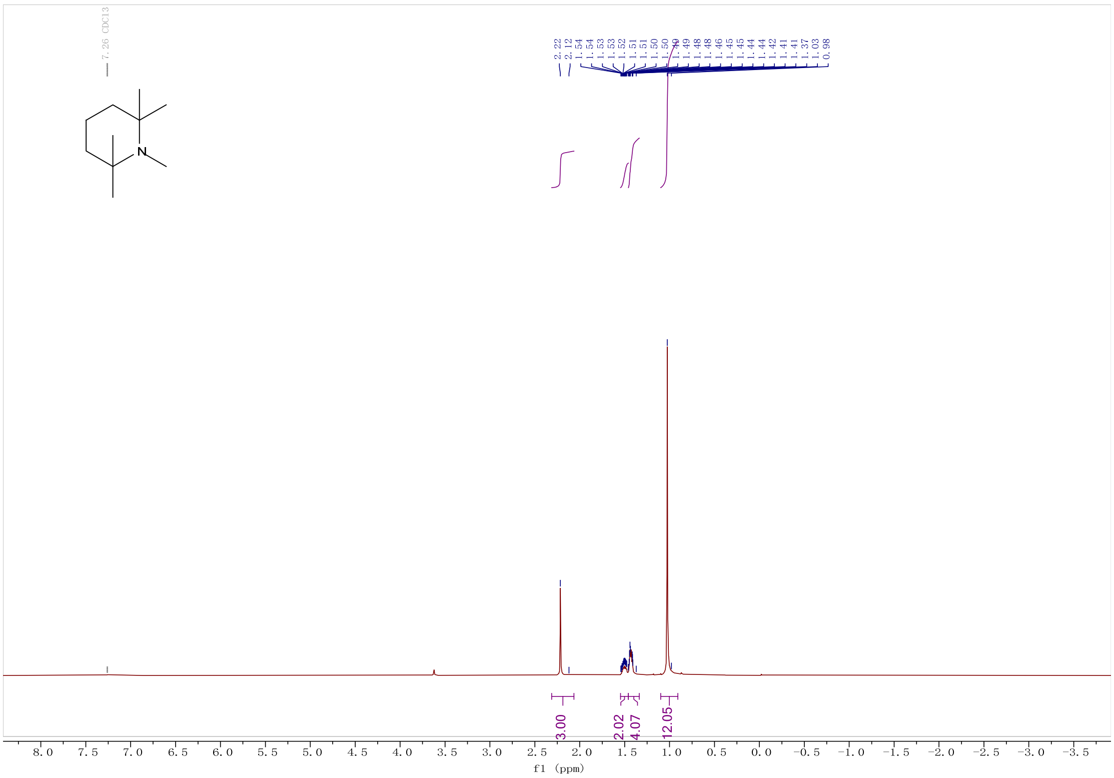

Figure S21. The ^{1}H-NMR spectrum of 1,2,2,6,6-pentamethylenepiperidine

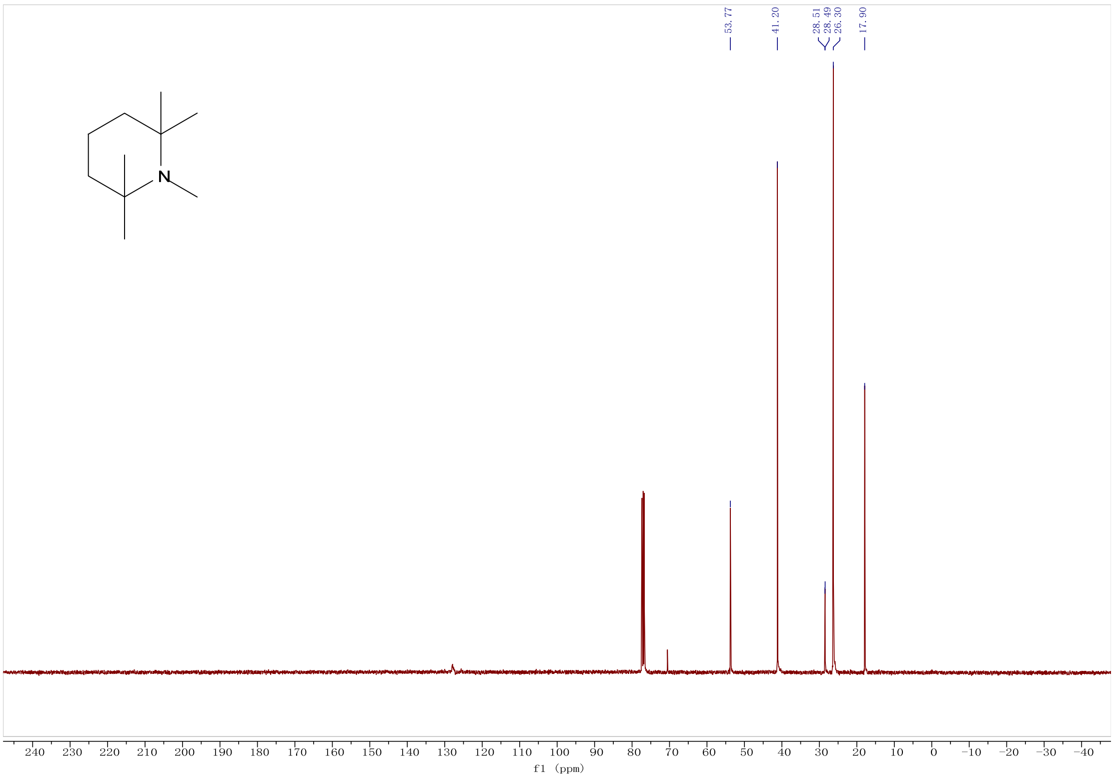

Figure S22. The ^{13}C-NMR spectrum of 1,2,2,6,6-pentamethylenepiperidine

N,N-Diisopropylmethylamine:^{1}H NMR (400 MHz, Chloroform-*d*) δ 2.93 (pd, *J* = 6.6, 1.9 Hz, 2H), 2.16 (d, *J* = 2.1 Hz, 3H), 1.03 (dd, *J* = 6.5, 1.5 Hz, 12H); ^{13}C NMR (101 MHz, Chloroform-*d*) δ 50.18, 31.21, 19.77.

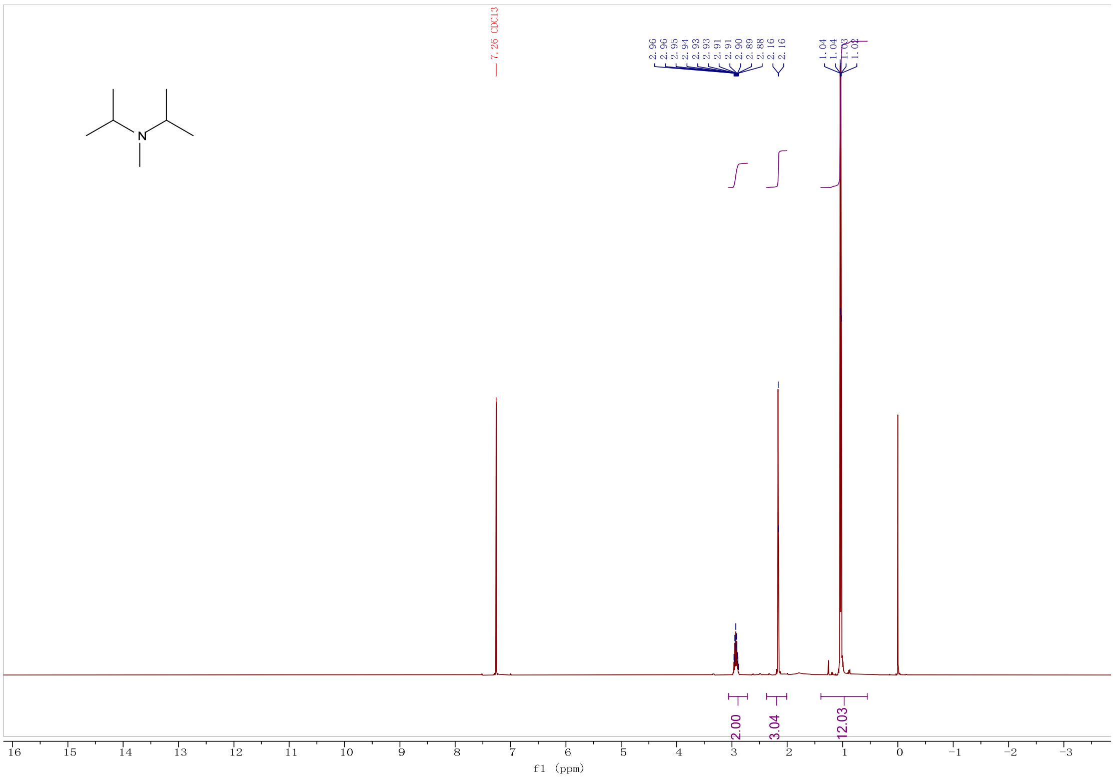

Figure S23. The ^{1}H-NMR spectrum of N,N-Diisopropylmethylamine

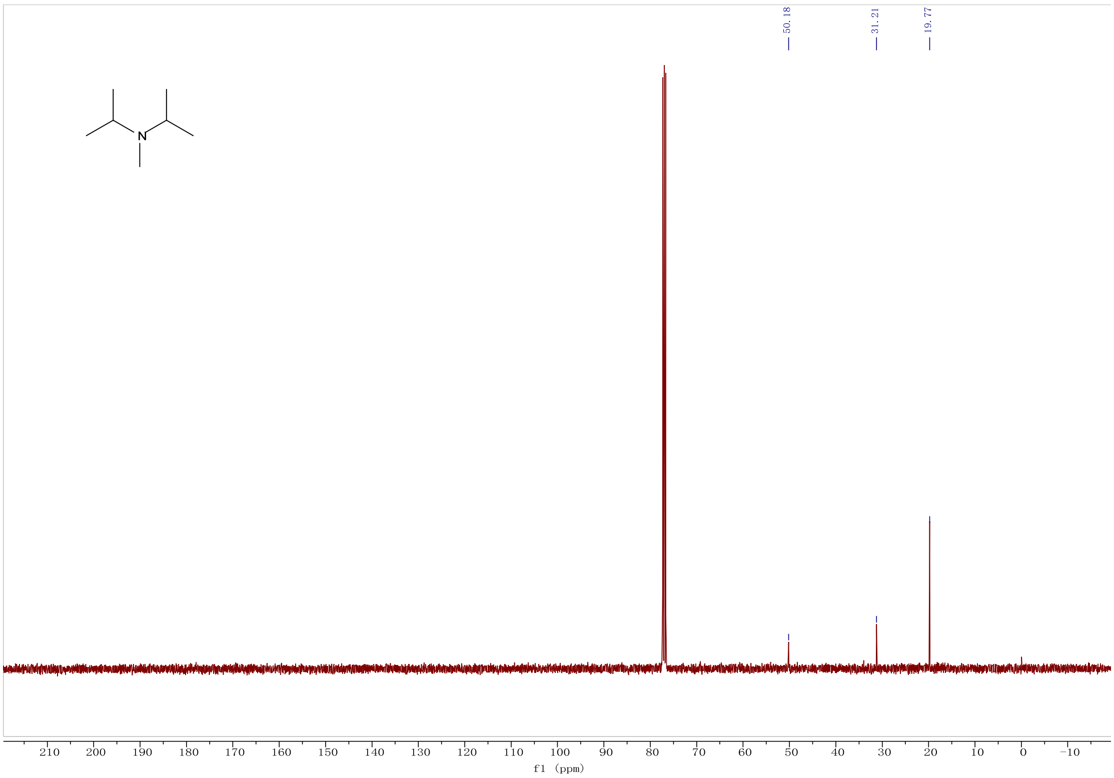

Figure S24. The ^{13}C-NMR spectrum of N,N-Diisopropylmethylamine

1-(Phenylmethyl)piperidine: ^{1}H NMR (400 MHz, Chloroform-*d*) δ 7.42 – 7.21 (m, 5H), 3.51 (s, 2H), 2.42 (t, J = 5.5 Hz, 4H), 1.61 (p, J = 5.6 Hz, 4H), 1.47 (p, J = 6.1 Hz, 2H); ^{13}C NMR (101 MHz, Chloroform-*d*) δ 138.72, 129.20, 128.08, 126.81, 63.90, 54.52, 26.03, 24.44.

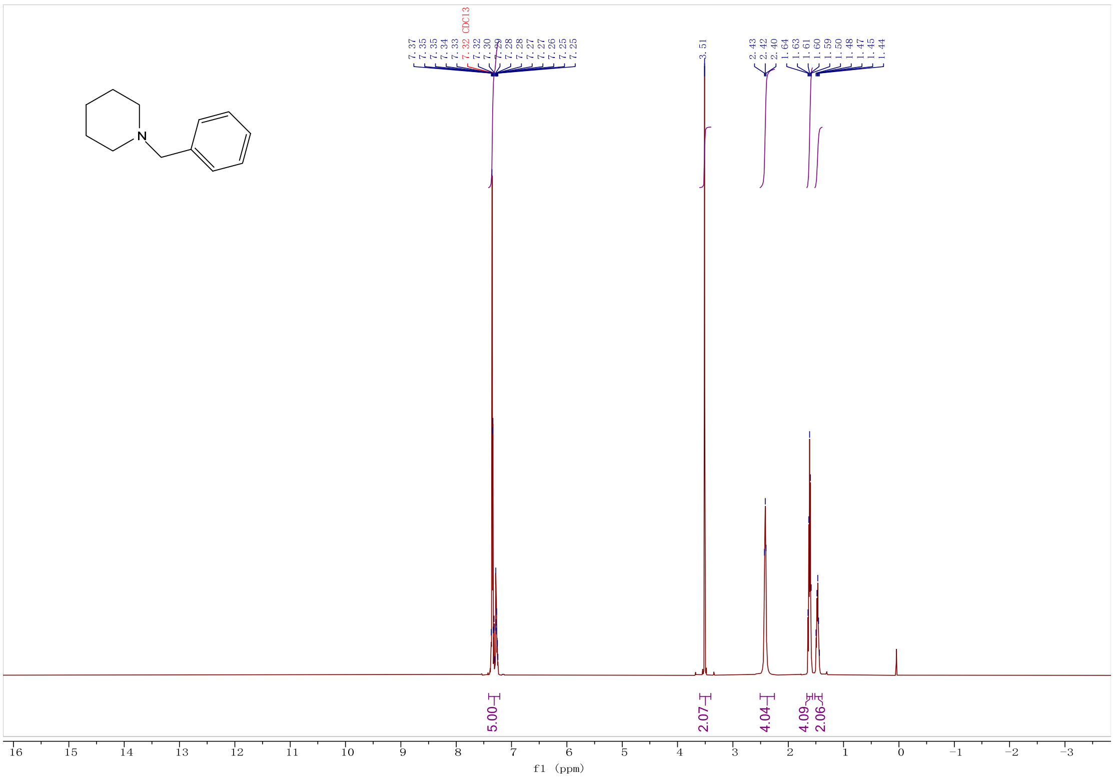

Figure S25. The ^{1}H-NMR spectrum of 1-(Phenylmethyl)piperidine

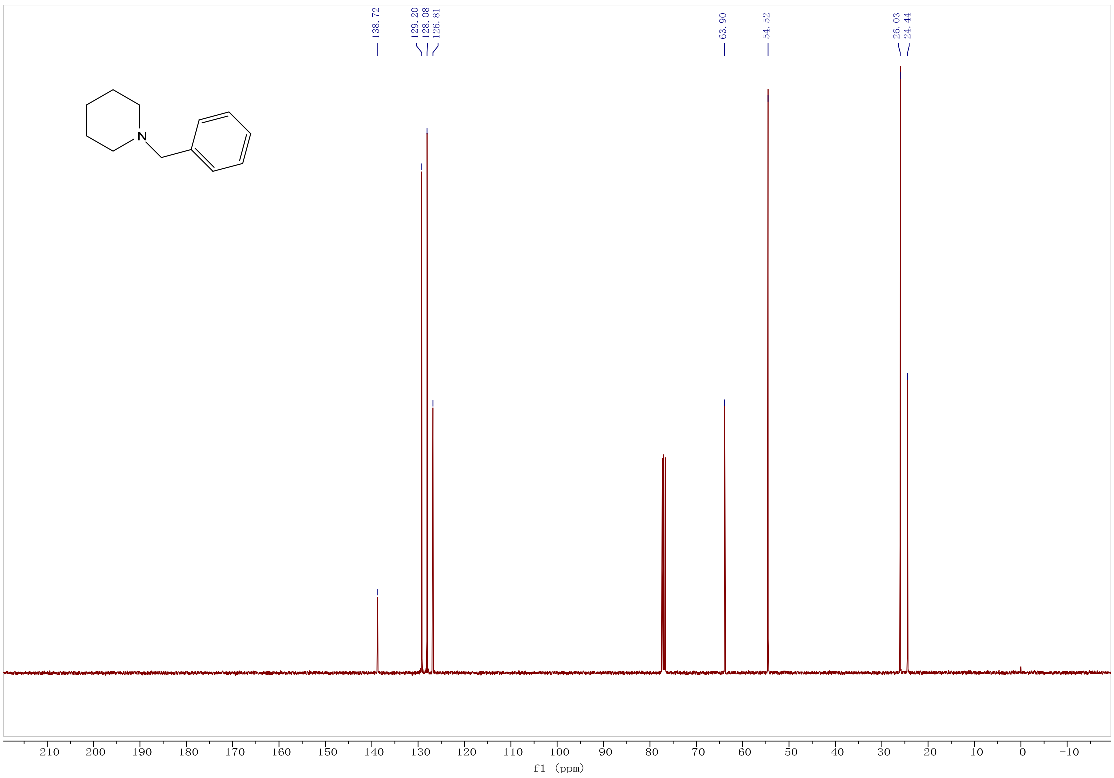

Figure S26. The ^{13}C-NMR spectrum of 1-(Phenylmethyl)piperidine

4-Methylmorpholine: ^{1}H NMR (400 MHz, Chloroform-*d*) δ 3.70 (q, *J* = 4.3 Hz, 4H), 2.39 (q, *J* = 4.3 Hz, 4H), 2.27 (d, *J* = 4.1 Hz, 3H); ^{13}C NMR (101 MHz, Chloroform-*d*) δ 66.90, 55.44, 46.41.

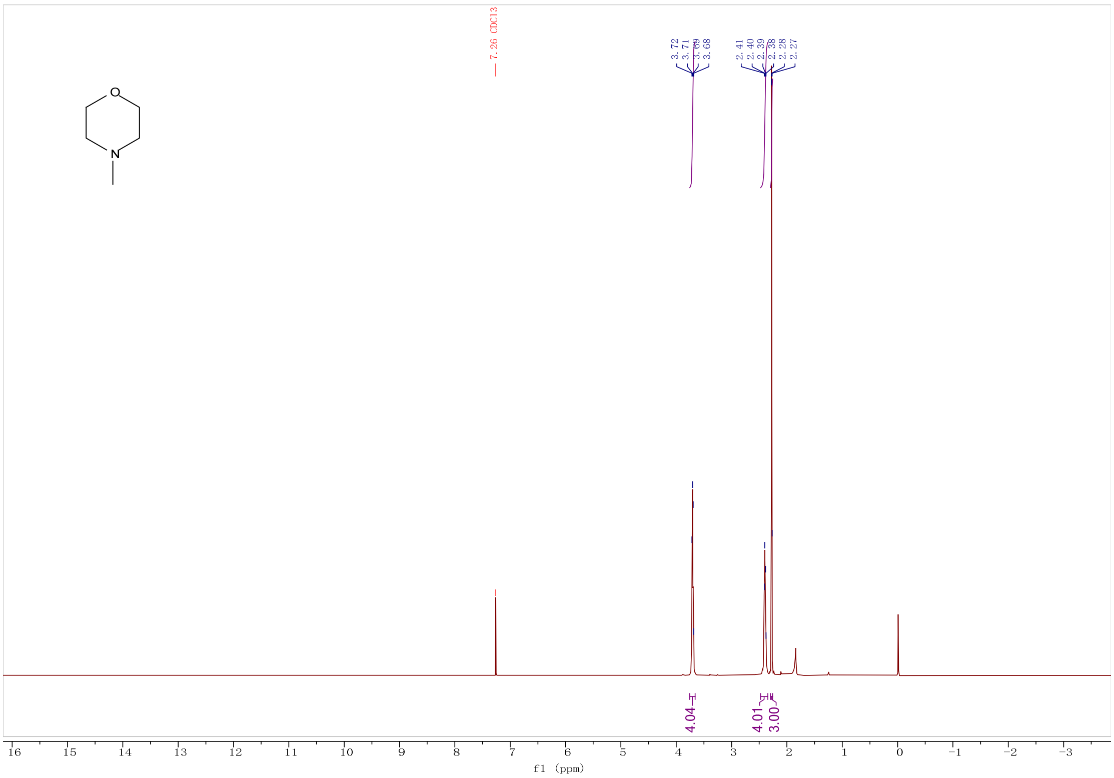

Figure S27. The ^{1}H-NMR spectrum of 4-Methylmorpholine

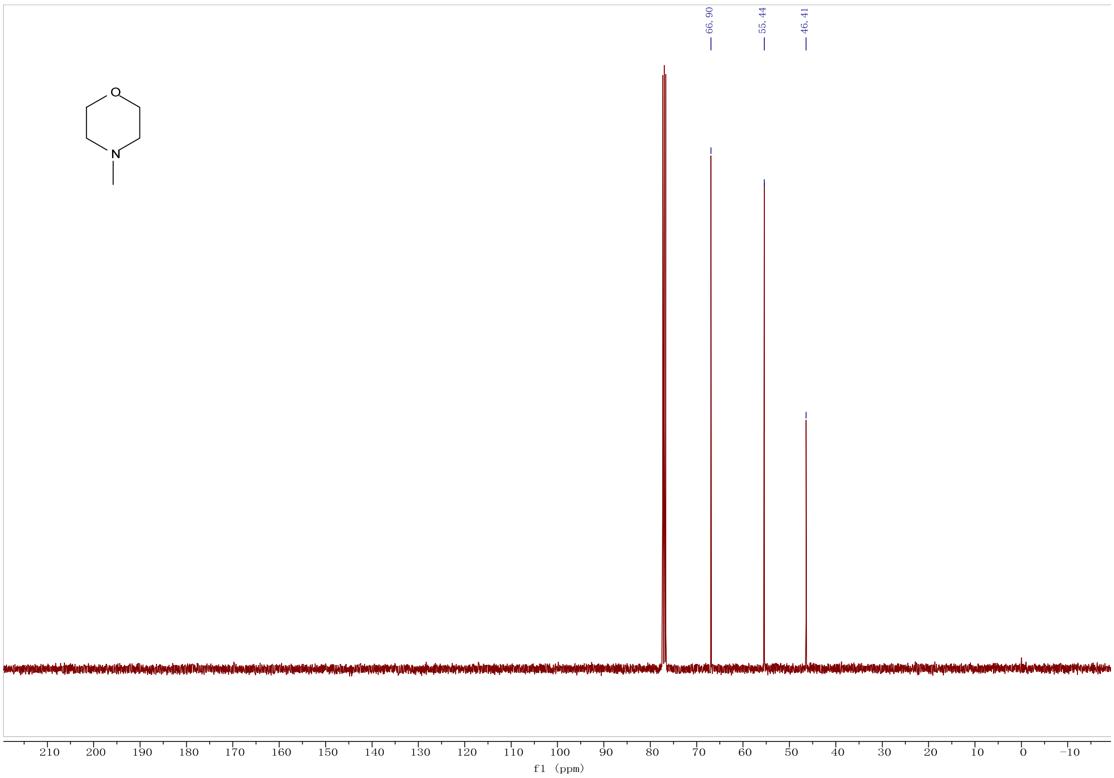

Figure S28. The ^{13}C-NMR spectrum of 4-Methylmorpholine

N-benzyl morpholine: ^{1}H NMR (400 MHz, Chloroform-*d*) δ 7.50 – 6.95 (m, 5H), 3.84 – 3.65 (m, 4H), 3.53 (s, 2H), 2.44 (t, $J = 4.4$ Hz, 4 H); ^{13}C NMR (101 MHz, Chloroform-*d*) δ 137.78, 129.19, 128.25, 127.14, 67.02, 63.46, 53.64.

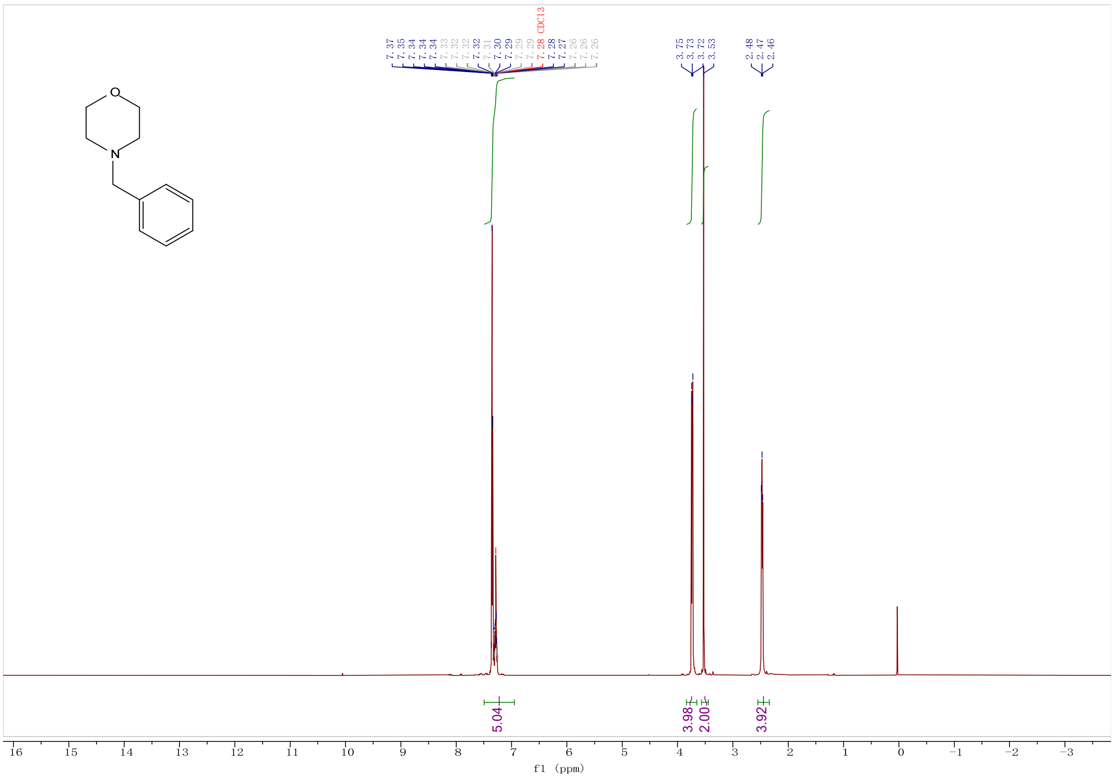

Figure S29. The ^{1}H-NMR spectrum of N-benzyl morpholine

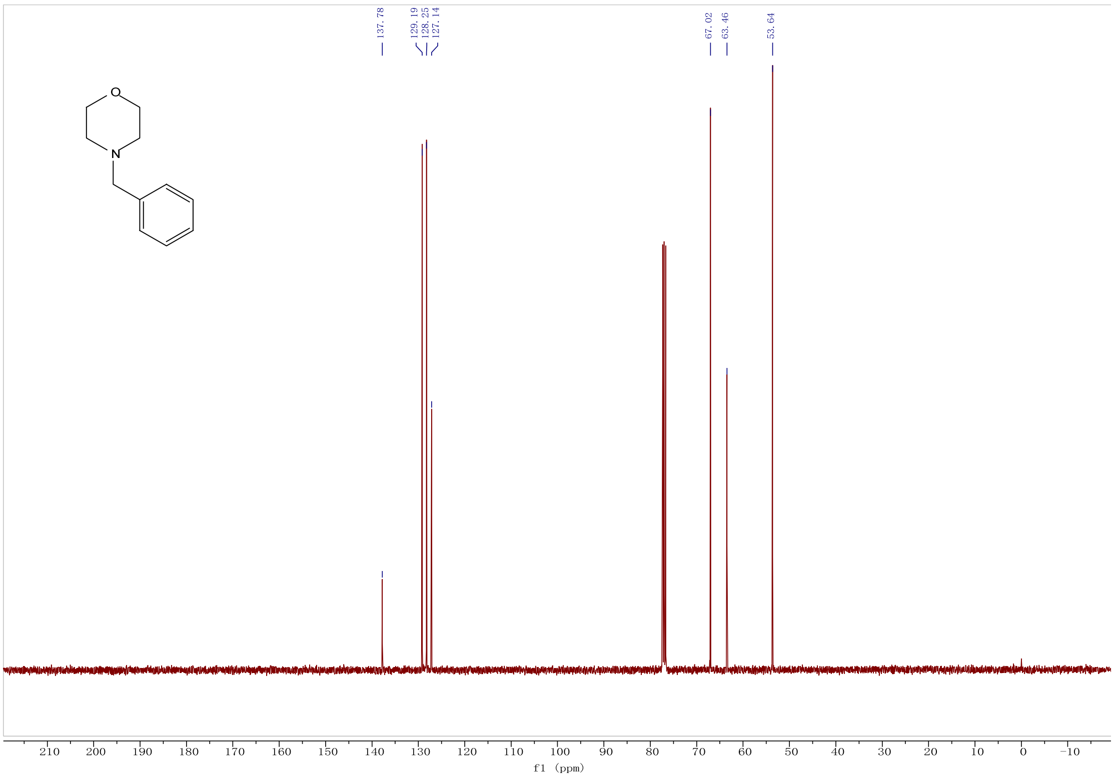

Figure S30. The ^{13}C-NMR spectrum of N-benzyl morpholine

1-Ethylpiperidine: ^{1}H NMR (400 MHz, Chloroform-*d*) δ 2.38 (q, *J* = 7.0 Hz, 6H), 1.60 (p, *J* = 5.7 Hz, 4H), 1.45 (q, *J* = 6.0 Hz, 2H), 1.08 (t, *J* = 7.2 Hz, 3H); ^{13}C NMR (101 MHz, Chloroform-*d*) δ 54.15, 52.99, 25.95, 24.51, 11.94.

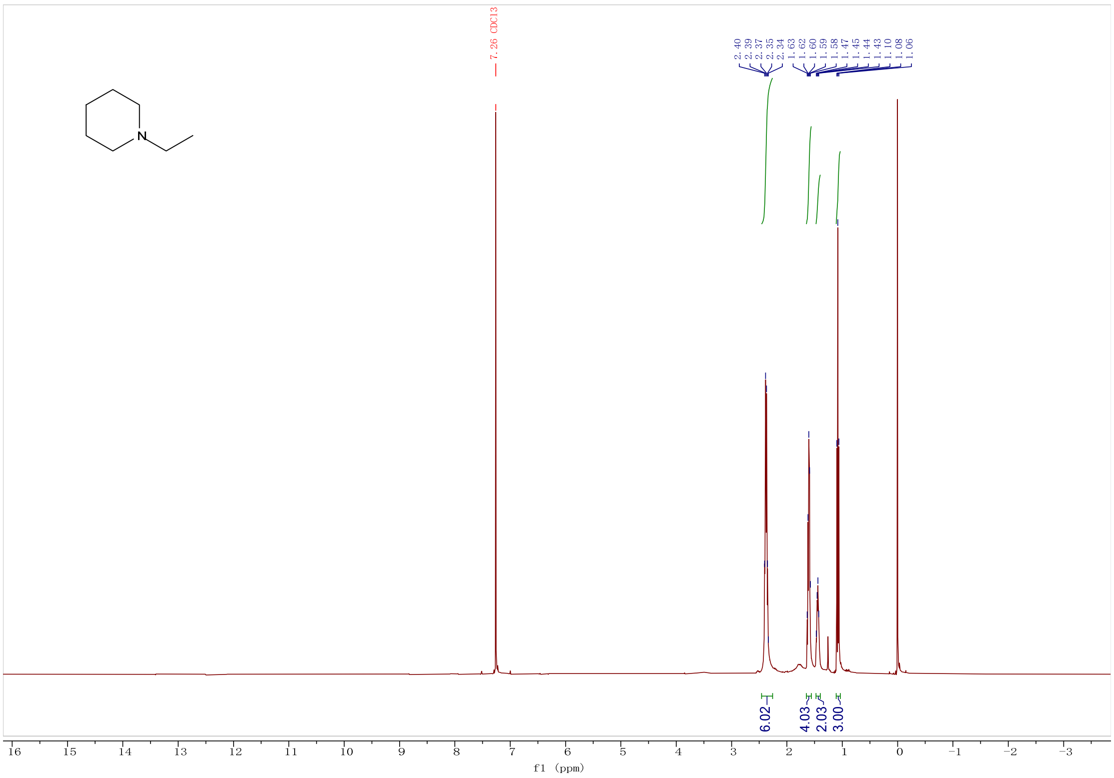

Figure S31. The ^{1}H-NMR spectrum of 1-Ethylpiperidine

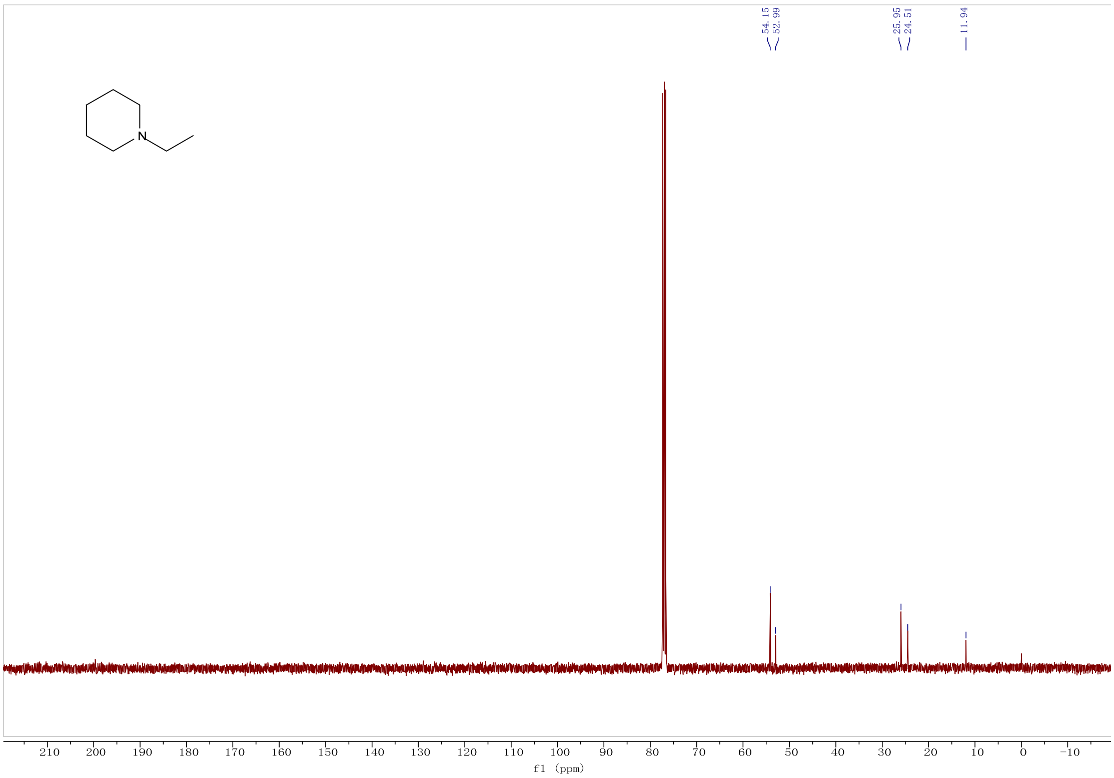

Figure S32. The ^{13}C-NMR spectrum of 1-Ethylpiperidine

1-ethylpyrrolidine: ^{1}H NMR (400 MHz, Chloroform-*d*) δ 2.54 (s, 6H), 1.81 (d, J = 3.7 Hz, 4H), 1.15 (h, J = 6.9 Hz, 3H); ^{13}C NMR (101 MHz, Chloroform-*d*) δ 54.30, 50.79, 23.42, 14.96.

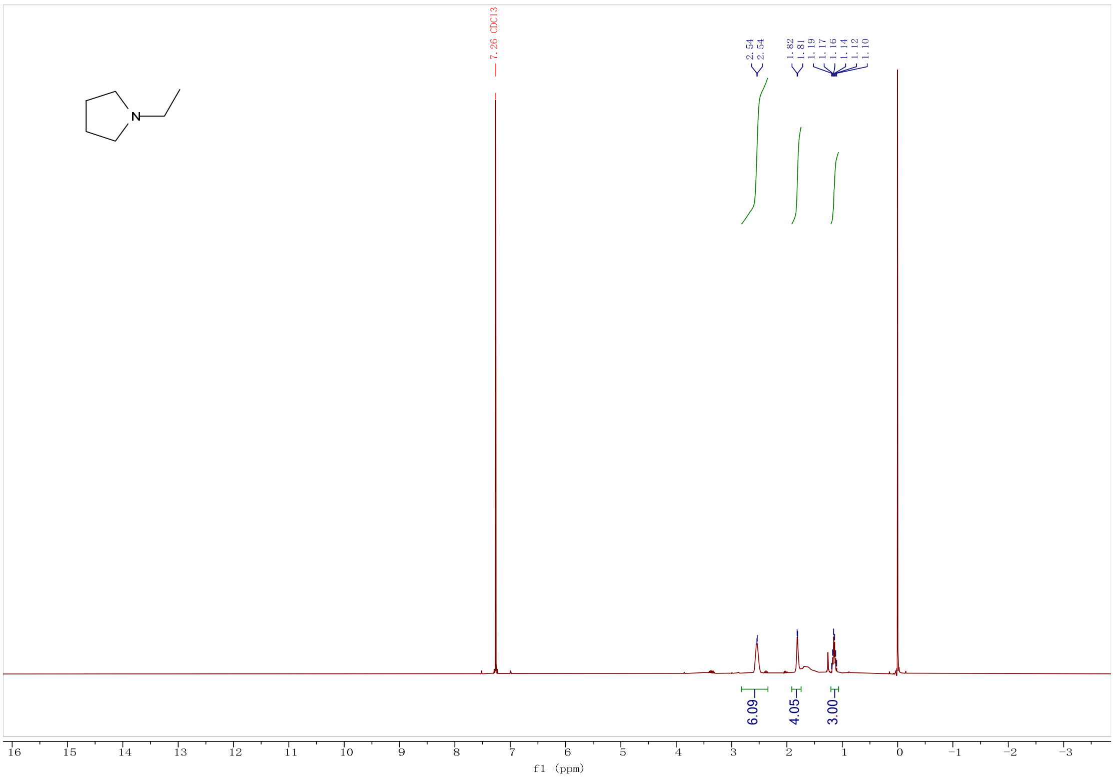

Figure S33. The ^{1}H-NMR spectrum of 1-ethylpyrrolidine

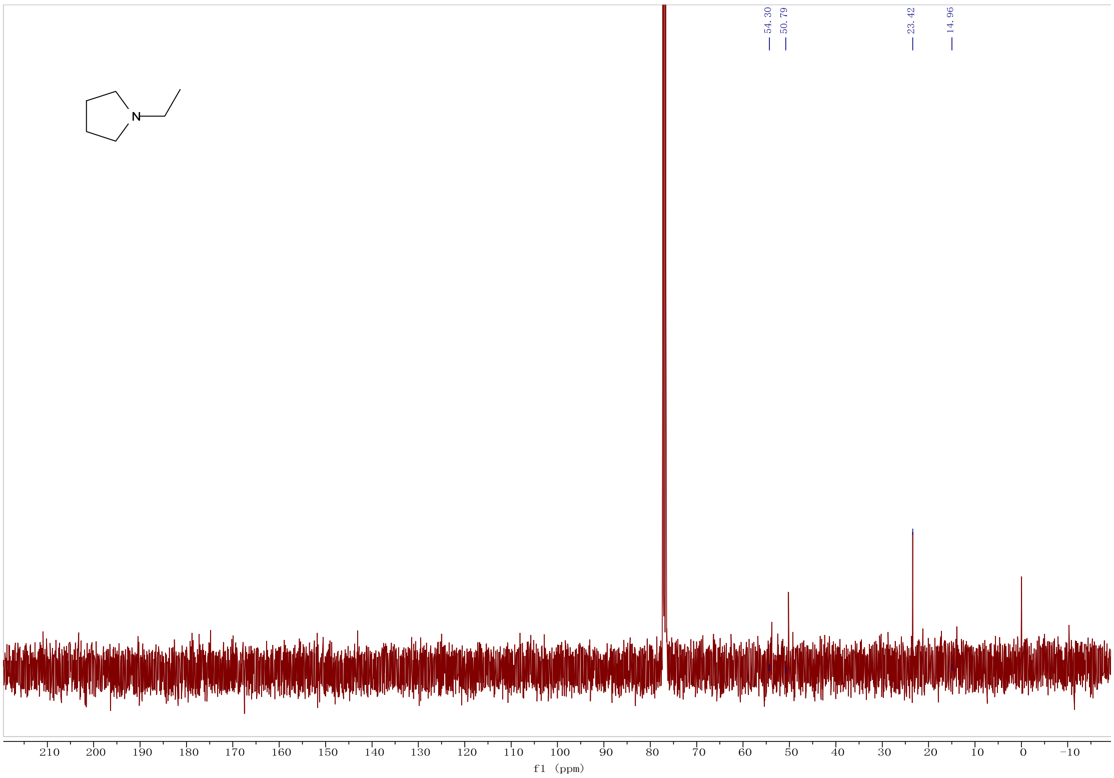

Figure S34. The ^{13}C-NMR spectrum of 1-ethylpyrrolidine

N,N-diethylaniline: ^{1}H NMR (400 MHz, Chloroform-*d*) δ 7.32 – 7.21 (m, 2H), 6.84 – 6.55 (m, 3H), 3.48 – 3.30 (m, 4H), 1.30 – 1.11 (m, 6H); ^{13}C NMR (101 MHz, Chloroform-*d*) δ 147.92, 129.25, 115.46, 112.01, 44.34, 12.60.

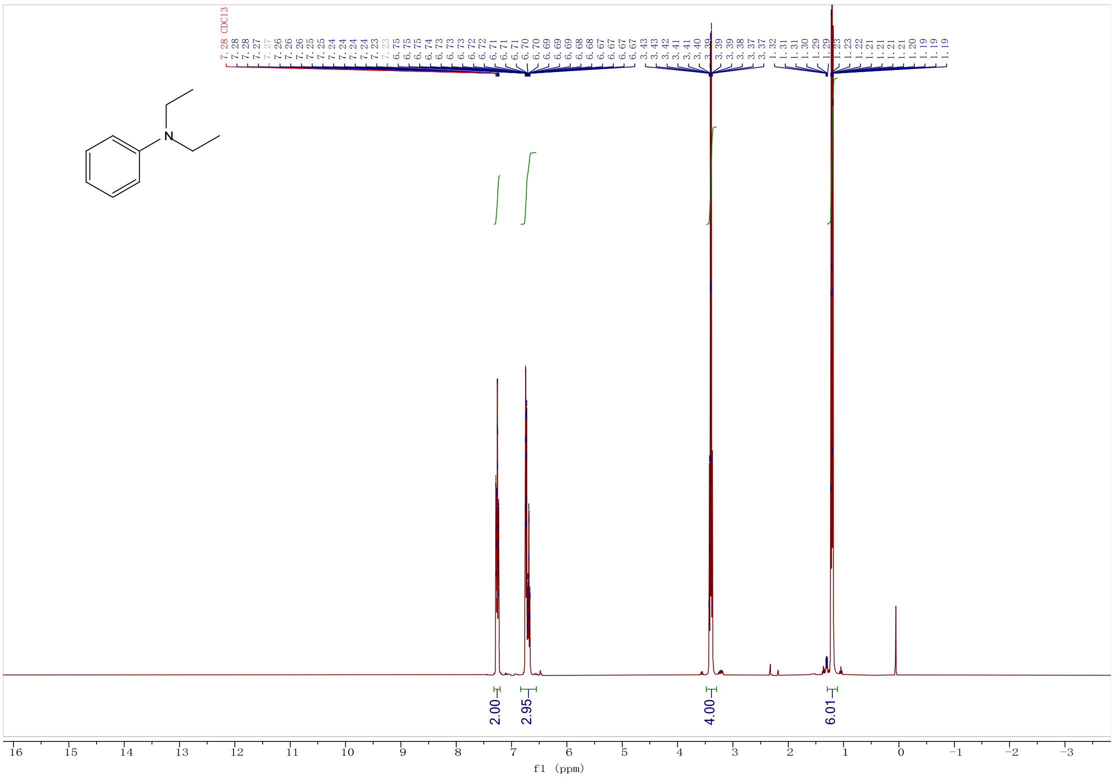

Figure S35. The ^{1}H-NMR spectrum of N,N-diethylaniline

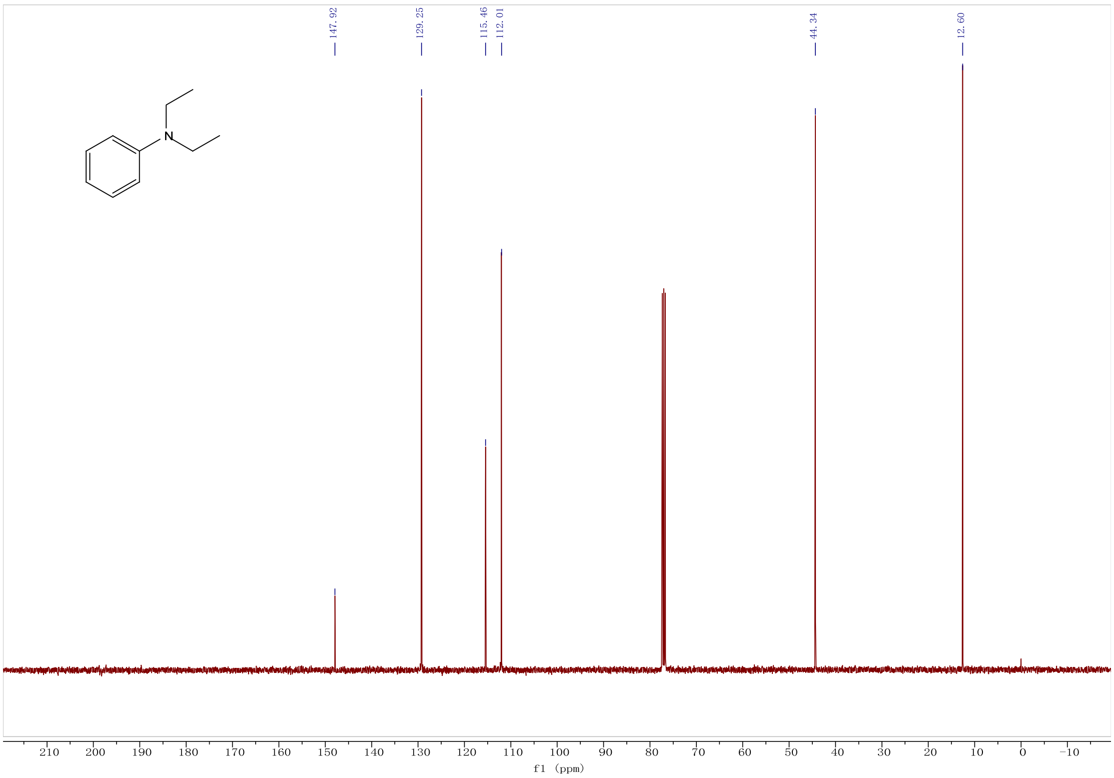

Figure S36. The ^{13}C-NMR spectrum of N,N-diethylaniline

N-methyldibutylamine: ^{1}H NMR (400 MHz, Chloroform-*d*) δ 2.35 – 2.21 (m, 4H), 2.17 (s, 3H), 1.47 – 1.37 (m, 4H), 1.28 (dq, *J* = 14.6, 7.2 Hz, 4H), 0.89 (t, *J* = 7.3 Hz, 6H); ^{13}C NMR (101 MHz, Chloroform-*d*) δ 57.63, 42.33, 29.48, 20.76, 14.05.

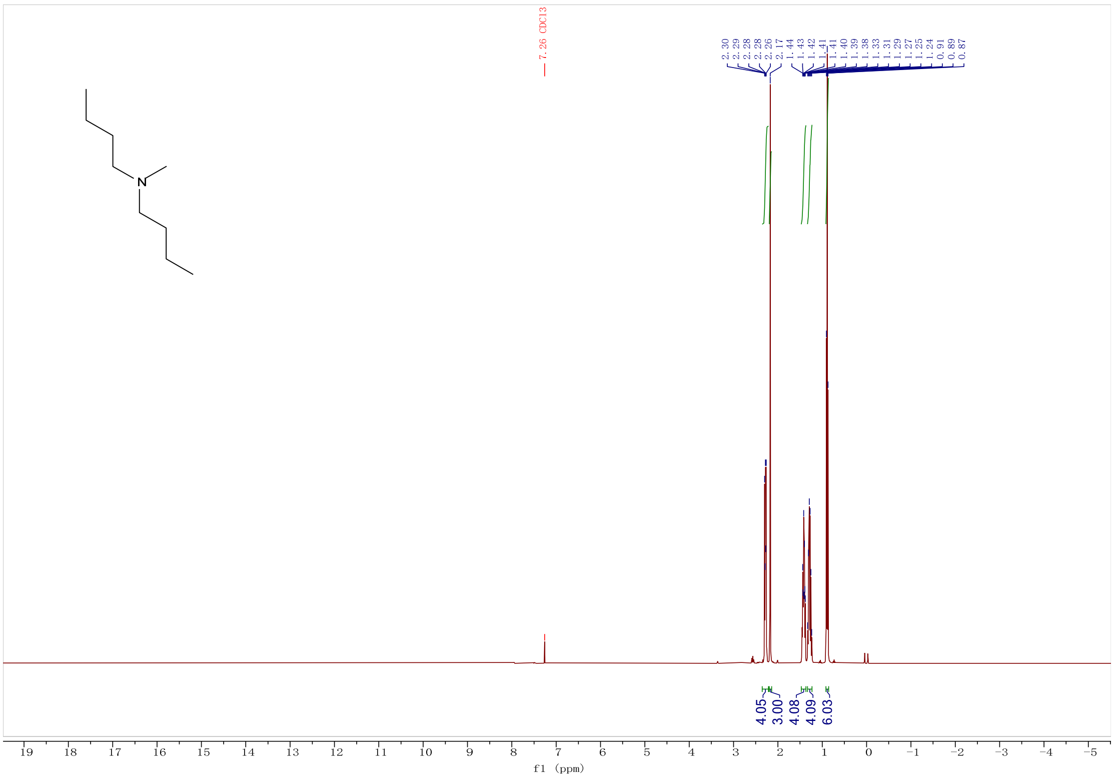

Figure S37. The ^{1}H-NMR spectrum of N-methyldibutylamine

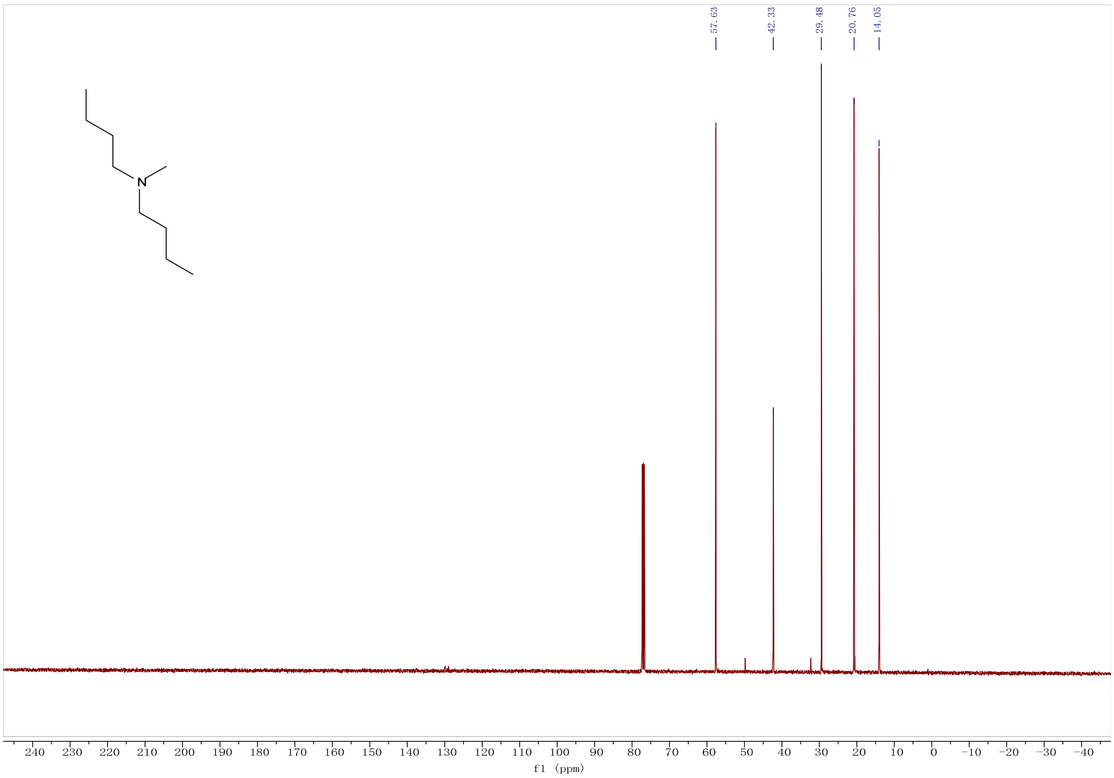

Figure S38. The ^{13}C-NMR spectrum of N-methyldibutylamine

N-butyldiisopropylamine: ^{1}H NMR (400 MHz, Chloroform-*d*) δ 2.94 – 3.03 (m, *J* = 6.6 Hz, 2H), 2.39 (t, *J* = 7.2 Hz, 2H), 1.39 – 1.33 (m, 2H), 1.31 – 1.20 (m, 2H), 0.98 (d, *J* = 6.6 Hz, 12H), 0.89 (t, *J* = 7.2 Hz, 3H); ^{13}C NMR (101 MHz, Chloroform-*d*) δ 48.56, 45.21, 33.85, 20.62, 20.59, 14.10.

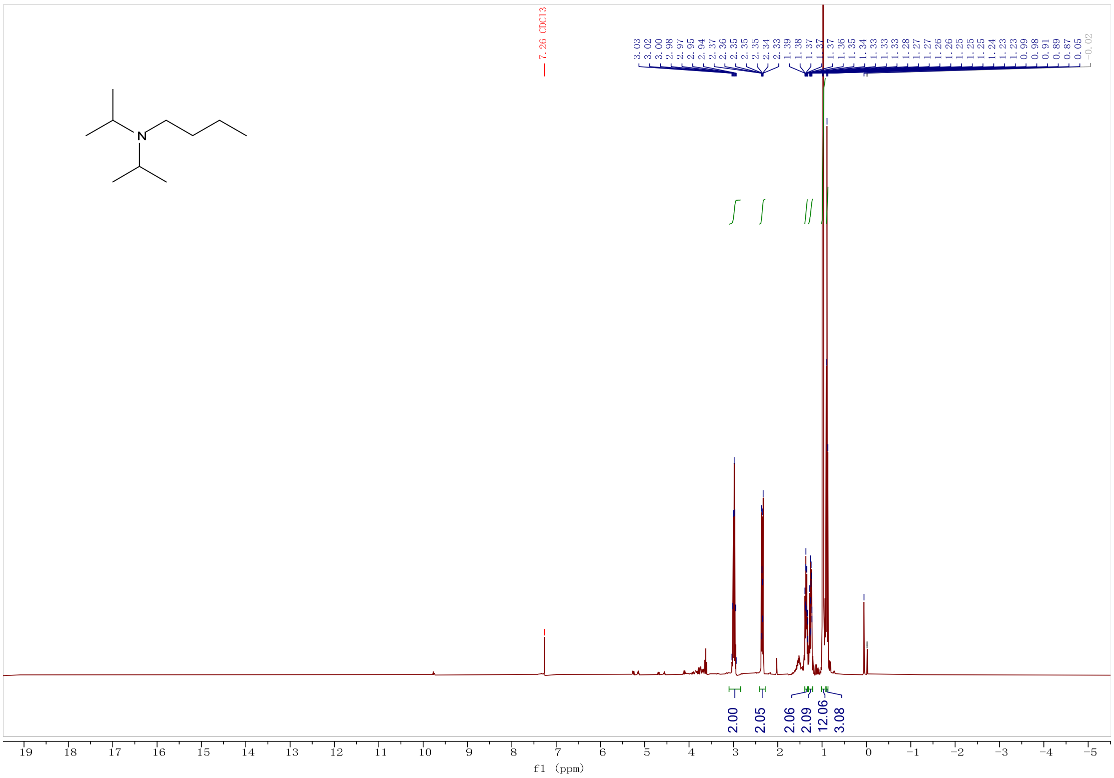

Figure S39. The ^{1}H-NMR spectrum of N-butyldiisopropylamine

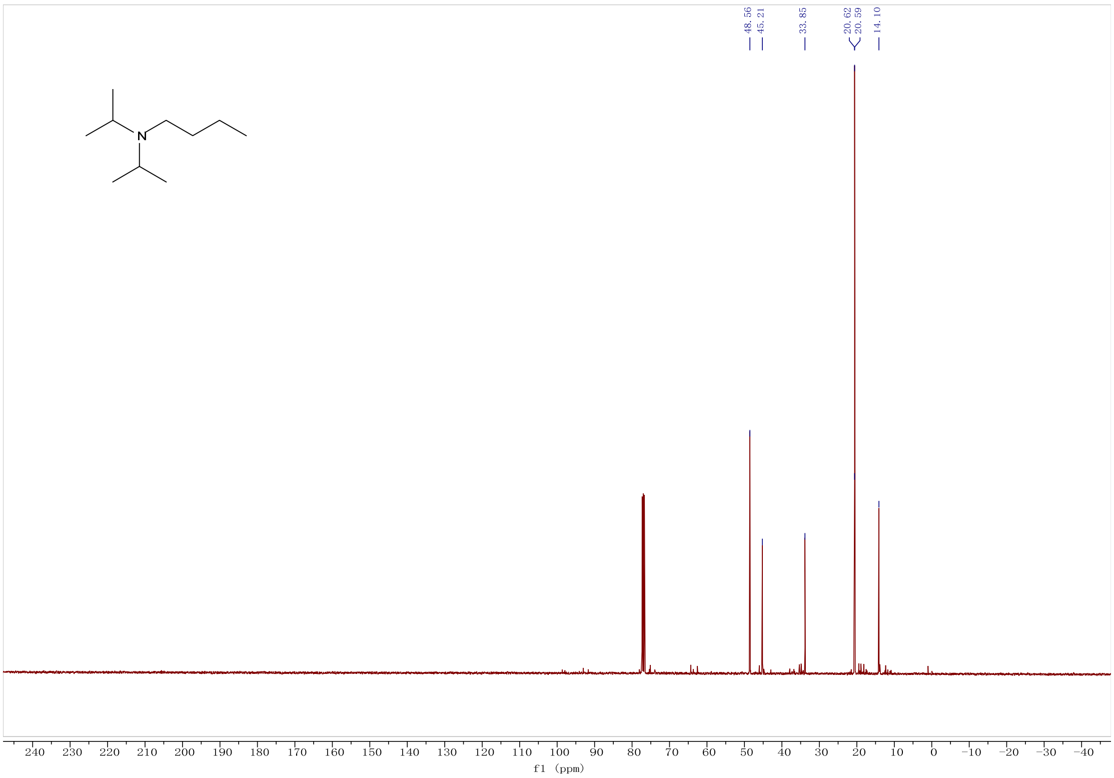

Figure S40. The ^{13}C-NMR spectrum of N-butyldiisopropylamine

N-Methyldicyclohexylamine: ^{1}H NMR (400 MHz, Chloroform-*d*) δ 2.47 (tt, *J* = 10.9, 2.8 Hz, 2H), 2.22 (s, 3H), 1.85 – 1.67 (m, 8H), 1.58 (d, 2H), 1.30 – 1.14 (m, 8H), 1.01 – 1.11 (m, 2H); ^{13}C NMR (101 MHz, Chloroform-*d*) δ 59.32, 32.87, 30.52, 26.32, 26.22.

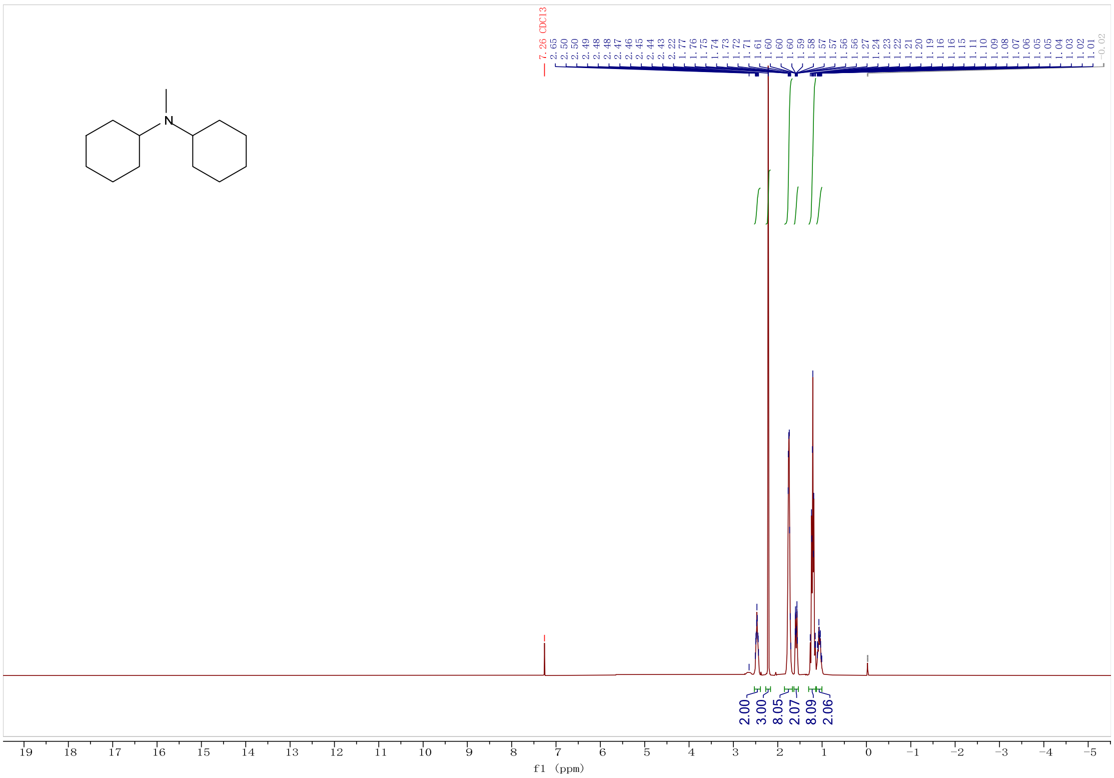

Figure S41. The ^{1}H-NMR spectrum of N-Methyldicyclohexylamine

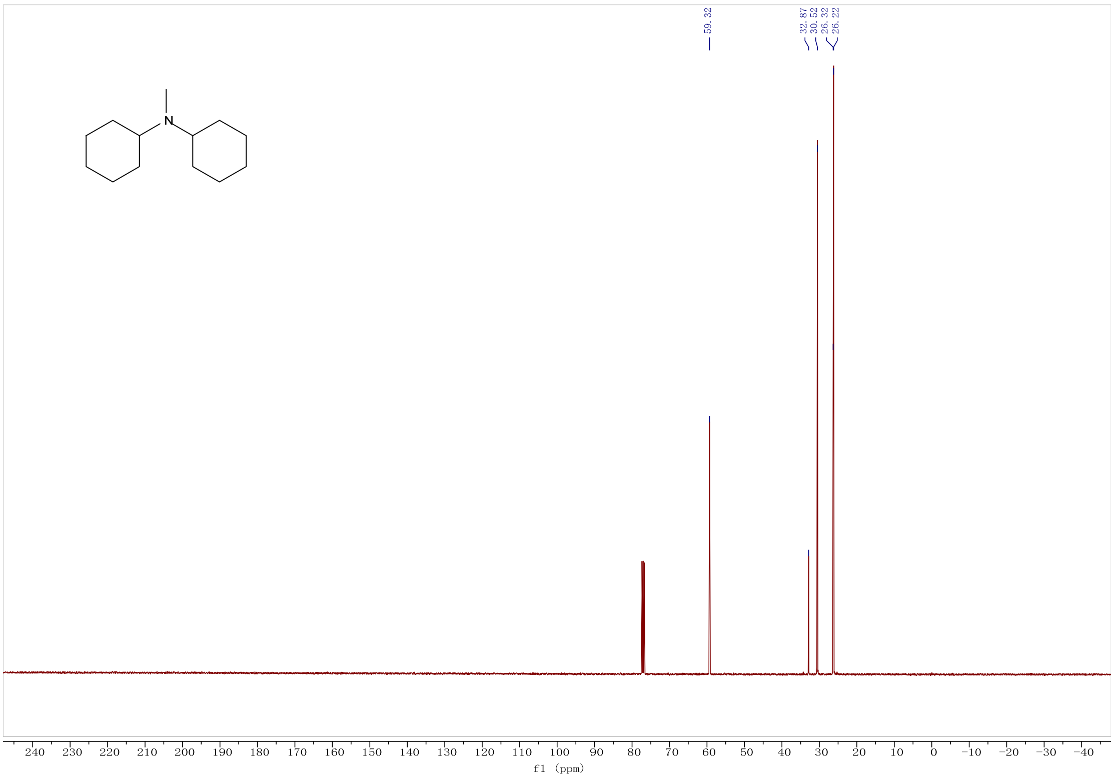

Figure S42. The ^{13}C-NMR spectrum of N-Methyldicyclohexylamine

N,N-dimethylcyclohexanamine: ^{1}H NMR (400 MHz, Chloroform-*d*) δ 2.26 (s, 6H), 2.13 (s, 1H), 1.93 – 1.68 (m, 4H), 1.67 – 1.54 (m, 1H), 1.29 – 1.04 (m, 5H); ^{13}C NMR (101 MHz, Chloroform-*d*) δ 63.68, 41.48, 28.87, 26.21, 25.68.

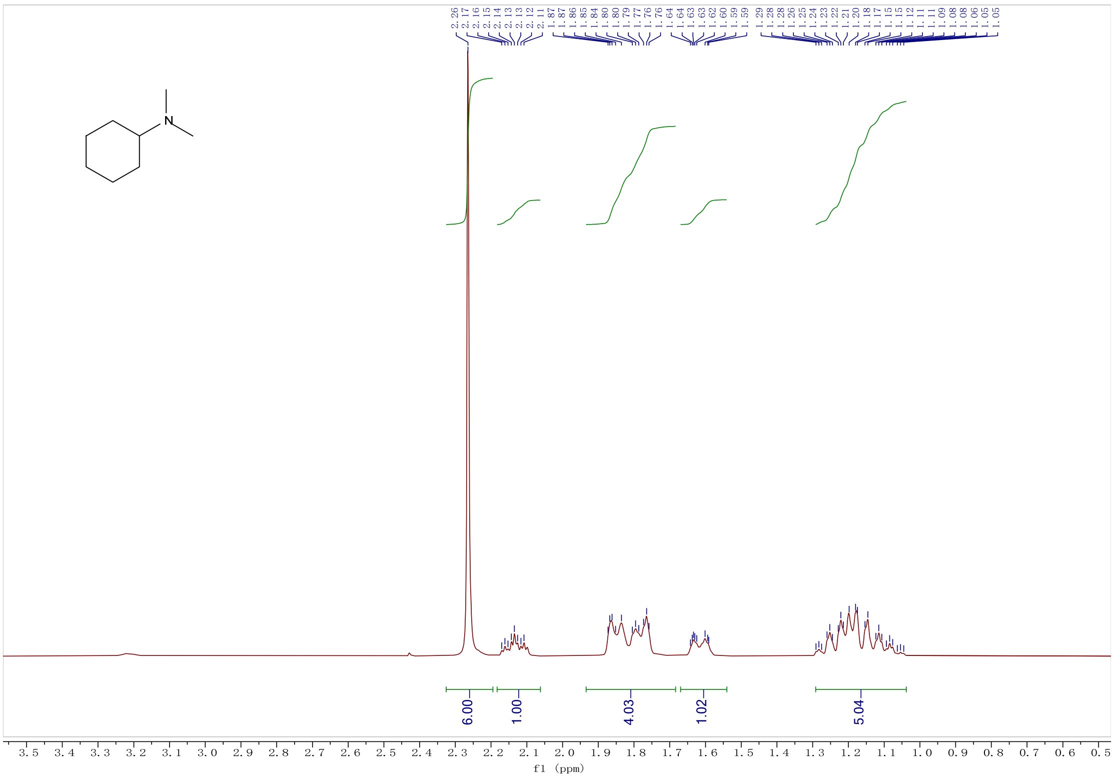

Figure S43. The ^{1}H-NMR spectrum of N,N-dimethylcyclohexanamine

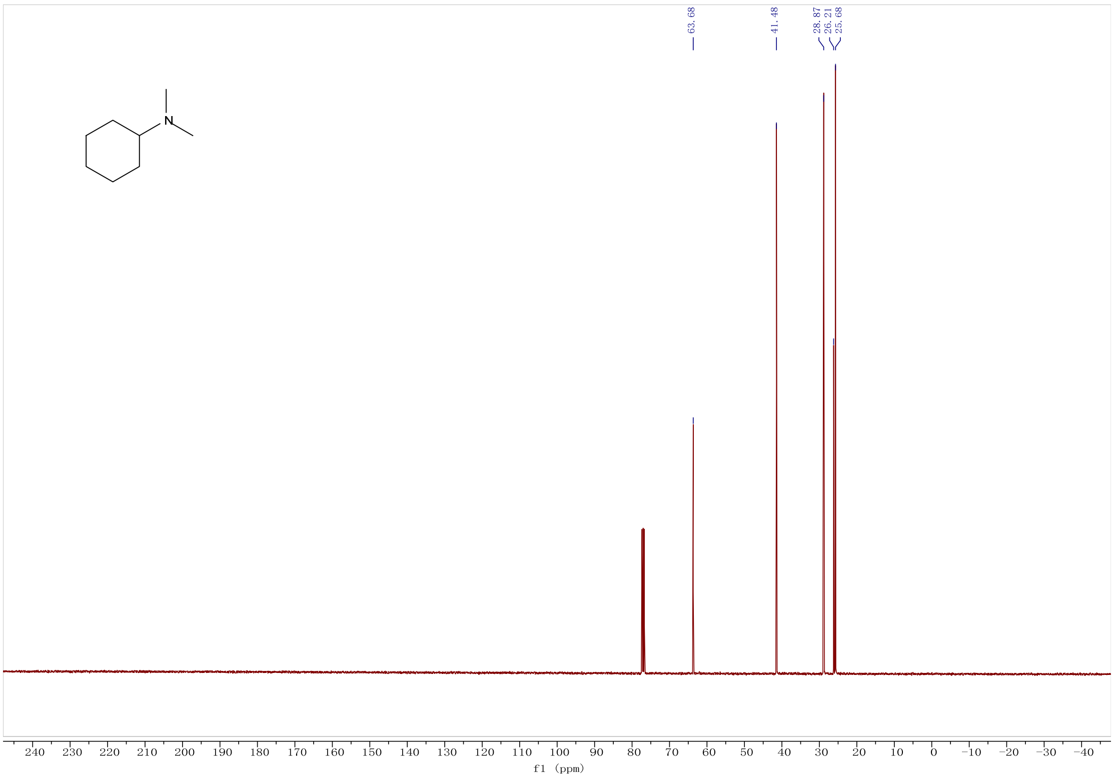

Figure S44. The ^{13}C-NMR spectrum of N,N-dimethylcyclohexanamine

N-Methyldiphenylamine: ^{1}H NMR (400 MHz, Chloroform-*d*) δ 7.32 – 7.18 (m, 4H), 7.05 – 6.96 (m, 4H), 6.93 (tt, *J* = 7.3, 1.2 Hz, 2H), 3.28 (s, 3H); ^{13}C NMR (101 MHz, Chloroform-*d*) δ 149.16, 129.32, 121.39, 120.57, 40.35.

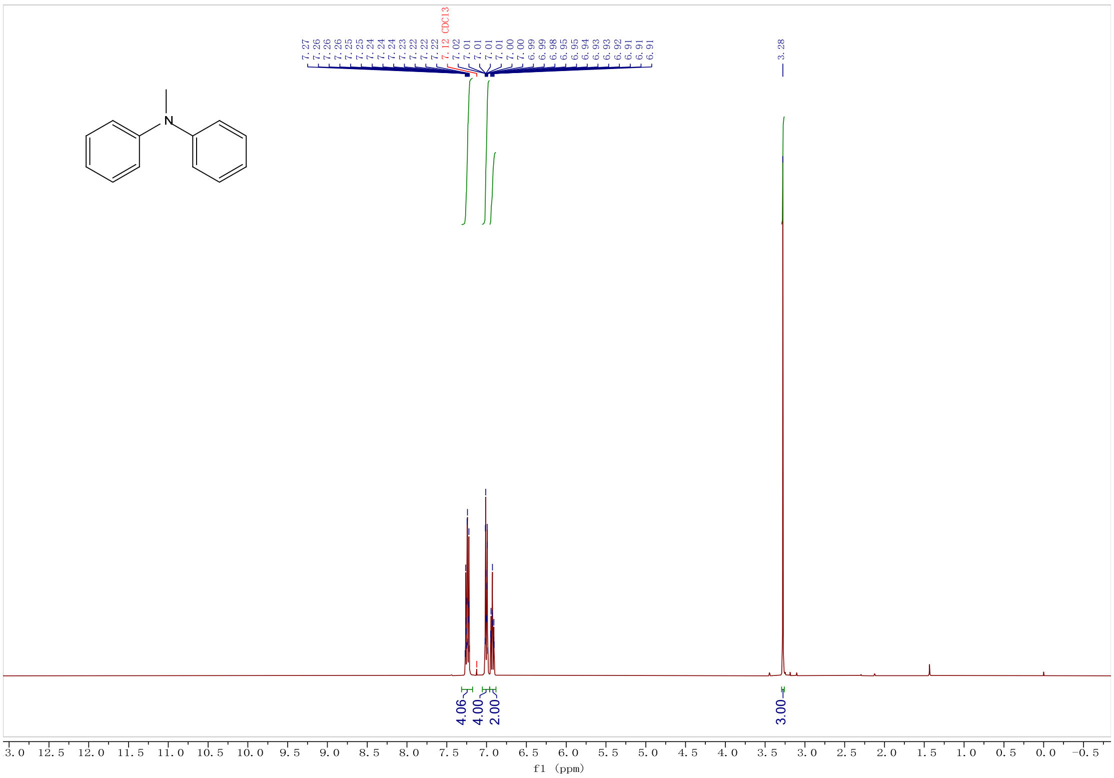

Figure S45. The ^{1}H-NMR spectrum of N-Methyldiphenylamine

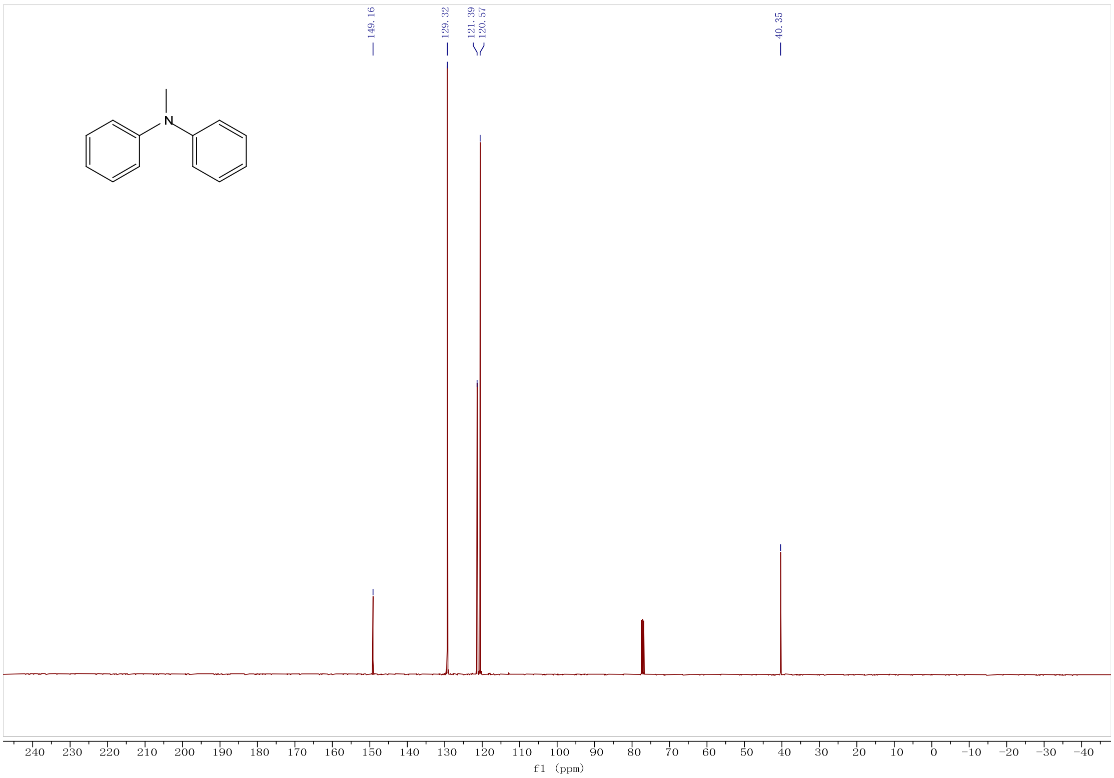

Figure S46. The ^{13}C-NMR spectrum of N-Methyldiphenylamine
